# Affective reactions differ between Chinese and American healthy young adults: a cross-cultural study using the international affective picture system

**DOI:** 10.1186/s12888-015-0442-9

**Published:** 2015-03-27

**Authors:** Jinwen Huang, Dongrong Xu, Bradley S Peterson, Jianbo Hu, Linfeng Cao, Ning Wei, Yingran Zhang, Weijuan Xu, Yi Xu, Shaohua Hu

**Affiliations:** Department of Mental Health, First Affiliated Hospital, Zhejiang University School of Medicine, Hangzhou, 310003 China; The Key Laboratory of Mental Disorder’s Management in Zhejiang Province, Hangzhou, 310003 China; Department of Psychiatry, Columbia University and New York State Psychiatric Institute, New York, 10032 USA; Institute of the Developing Mind, Children’s Hospital Los Angeles, University of Southern California, Los Angeles, California 90027 USA; Department of Clinic, First Affiliated Hospital, Zhejiang University School of Medicine, Hangzhou, 310003 China; Department of Psychiatry, Xiaoshan Hospital of Zhejiang, Hangzhou, 311201 China

**Keywords:** Affective reaction, Cross-culture, International Affective Picture System, Self-Assessment Manikin, Valence scores, Arousal scores

## Abstract

**Background:**

Several cross-cultural studies have suggested that emotions are influenced by the cultural background. Emotional reactions to International Affective Picture System (IAPS) images were compared between Chinese and American young adults.

**Methods:**

120 Chinese undergraduates (53 females, 67 males; aged 18-25 years) were enrolled at Zhejiang University, China, and the valence and arousal components of their emotional responses to IAPS images were rated using the Self-Assessment Manikin (SAM) system. Then, valence and arousal scores were compared to those of 100 American undergraduates (50 females, 50 males) of the same age group, enrolled at Florida University and surveyed by Prof. PJ Lang in 2001.

**Results:**

Valence scores assigned to 259/816 (31.74%) pictures differed significantly between Chinese and American female participants, while those assigned to 165/816 (20.22%) pictures differed significantly between Chinese and American males (P < 6 × 10^-5^). Of the 816 pictures, the arousal scores assigned to 101/816 (12.38%) pictures differed significantly between Chinese and American female participants; these scores significantly differed in 130/816 (15.93%) pictures between Chinese and American males (P < 6 × 10^-5^). Valence scores for pictures in the Erotic category differed significantly between Chinese and American females (P < 6 × 10^-5^). There were no significant differences in valence scores for the remaining eight categories studied between participants from the two countries, whether female or male.

**Conclusions:**

The IAPS norms require a modification for their appropriate application in Asian cultures.

**Electronic supplementary material:**

The online version of this article (doi:10.1186/s12888-015-0442-9) contains supplementary material, which is available to authorized users.

## Background

Several studies have demonstrated that the cultural background exerts a significant influence on emotional expression and processing [1-6]. Grossmann *et al.* found that Russians spent significantly more time looking at negative pictures than positive ones, whereas Americans do not display this inclination [2]. They also demonstrated that Russian Latvians recognize negative words after priming with Russian cultural symbols significantly faster than when Latvian cultural symbols are used [2]. Hystad *et al.* have recently identified differences between the emotional intelligence scores of Norwegian and American undergraduate students [7]. The differences between the emotional reactions of Chinese and American adults were also highlighted in a study assessing the fear of one’s employer [8]. Indeed, Liew *et al.* found that Americans, unlike Chinese participants, do not show a “boss effect” and maintain self-face advantage in the presence of their supervisor’s face. Self-face advantage is known as an individual characteristic in which human adults typically response faster to their own face than to the faces of others. However, their self-face advantage decreased as their ratings of their boss's perceived social status increased, suggesting that self-processing in Americans is influenced more by one's social status than the hierarchical position. Therefore, the very concept of social position, such as a boss, may hold markedly different meanings to the self across Western and East Asian cultures [8]. Another study demonstrated that Japanese participants tend to ignore vocal emotional expressions and instead focus on facial expressions, suggesting that multisensory integration of affective information is modulated by the perceiver's cultural background [9]. Furthermore, it was indicated that a number of primarily negative emotions have vocalizations that can be recognized across cultures, while most positive emotions are communicated with culture-specific signals [9]. The basic emotions, including anger, fear, disgust, happiness, sadness, and surprise, were shown to be reliably identified by both English and Himba listeners from vocalizations produced by individuals of both groups. In contrast, vocalizations of several positive emotions (achievement/triumph, relief, and sensual pleasure) were not recognized bi-directionally by both groups of listeners [9]. Similarly, only modest recognition rates for affection, guilt, pride, and shame were obtained across cultures [9]. Cultures vary as for the conceptualization of self, which may lead to culture-specific interpretations of situations particularly relevant to self-conscious emotions such as pride and shame [9]. However, the importance of cross-cultural differences in affective perception was demonstrated, not only of non-verbal vocalization expressing affect, but also of vocalizations expressing basic negative emotions [9]. Japanese listeners were shown to have less intense and negative ratings for angry, disgusted, and fearful vocalizations from the Montreal Affective Voices compared with Canadian listeners. Similarly, pleased vocalizations were rated as less intense and less positive by Japanese listeners [9].

The International Affective Picture System (IAPS) has been developed to provide a set of emotional stimuli to assist in research assessing human emotions and attention. IAPS includes 823 standardized, emotionally evocative, internationally accessible, color photographs spanning nine general categories: animal, facial, mutilation, erotic, landscape, violence, objects, sport and pollution [10]. The emotional response to individual pictures can be assessed in the following three dimensions: valence (happy versus unhappy), arousal (excited versus calm), and dominance (controlled versus in-control). The IAPS has been used in many functional neuroimaging studies [11] and assessment of a broad range of psychiatric disorders [12].

Based on the evidence from cross-cultural studies of emotion, we hypothesized that the emotional reactions induced by IAPS pictures may be regulated by non-western culture such as Asian life style. In the present study, young Chinese participants were shown IAPS pictures, and valence and arousal scores were obtained and compared with those of age-matched Americans at the University of Florida, surveyed by Lang, the IAPS author, in 2001. Differences between Chinese and American young adults were explored. To our knowledge, this is the first assessment of IAPS in a Chinese population. The present study may help Asian researchers use the IAPS system appropriately.

## Methods

### Chinese participants

This study was performed at Zhejiang University, Hangzhou, China, from January to October 2012. It was approved by the Human Investigations Committee of Zhejiang University. The study aims and experimental procedures were explained to all participants, who provided written informed consent before enrolment. Informed consents also ensured some monetary compensation for all participants.

Many studies have demonstrated that smoking plays a significant role in regulating emotion: ex-smokers who have stopped for a year or more, similar to people who have never smoked, are happier than current smokers [8,9,11]. Furthermore, smoking is associated with lower fear, control and drive, higher anger and unstable externalized affective temperaments. Lower control and higher anger were shown to be associated with heavy and current smoking statuses [8,9,11]. It is known that handedness may affect the detection of dichotically-presented words and emotions [8,9,11]. Based on the above evidence, only right-handed non-smokers, not using alcohol, with no history of significant psychiatric or physical diseases could participate in the study.

Students from Zhejiang University were enrolled through advertisements posted on campus. Inclusion criteria were: 18-25 years old healthy undergraduate student; right-handed; no history of mental disorder or neurological disease. Exclusion criteria were: history of mood disorder or any other psychiatric disorder, history of neurological disease or any serious physical disease, drug abuse, treatment with psychiatric drugs in the last 3 months; smoking, or alcohol drinking.

In total, 128 undergraduate participants (58 females, 70 males) were screened by self-reported questionnaire and psychiatric interview. The questionnaire included the demographic information, history of any psychiatric disorder, physical disease, drugs, smoking or alcohol, and handedness. The questionnaire is provided as Additional file 1. Two psychiatrists completed a mental status examination for each participant in an isolated room. At last, six participants (4 females, 2 males) were excluded because of depression symptoms. Two participants (1 female, 1 male) had a history of insomnia and were excluded as well. No patient had to be excluded because of left handedness. Finally, 120 undergraduate students (53 females, 67 males, 18-25 years old) took part in the present study. Female and male participants were aged 21.43 ± 1.51 and 21.26 ± 1.65 years, respectively. The educational levels of females and males were 14.38 ± 0.84 and 14.60 ± 1.02 years, respectively. Of those, 43.40% females and 38.81% males were from rural areas.

### Experimental procedures

IAPS pictures were used as visual stimuli to induce affective reactions. We excluded seven pictures that did not have proper norms. Therefore, the valence and arousal dimensions of affective responses to 816 images [8,9,11] were assessed in Chinese participants (available from: http://www.unifesp.br/dpsicobio/adap/instructions.pdf). The 816 pictures were randomized and divided into three sets. The pictures were presented using the Presentation software (Neurobehavioral Systems, Inc., Albany, CA, USA). Each participant was seated, facing a screen to which the pictures were projected; consecutive sets were separated by a 15-minute break. Each picture was presented for 4 seconds followed by a gray screen for 6 seconds.

**Figure 1:**
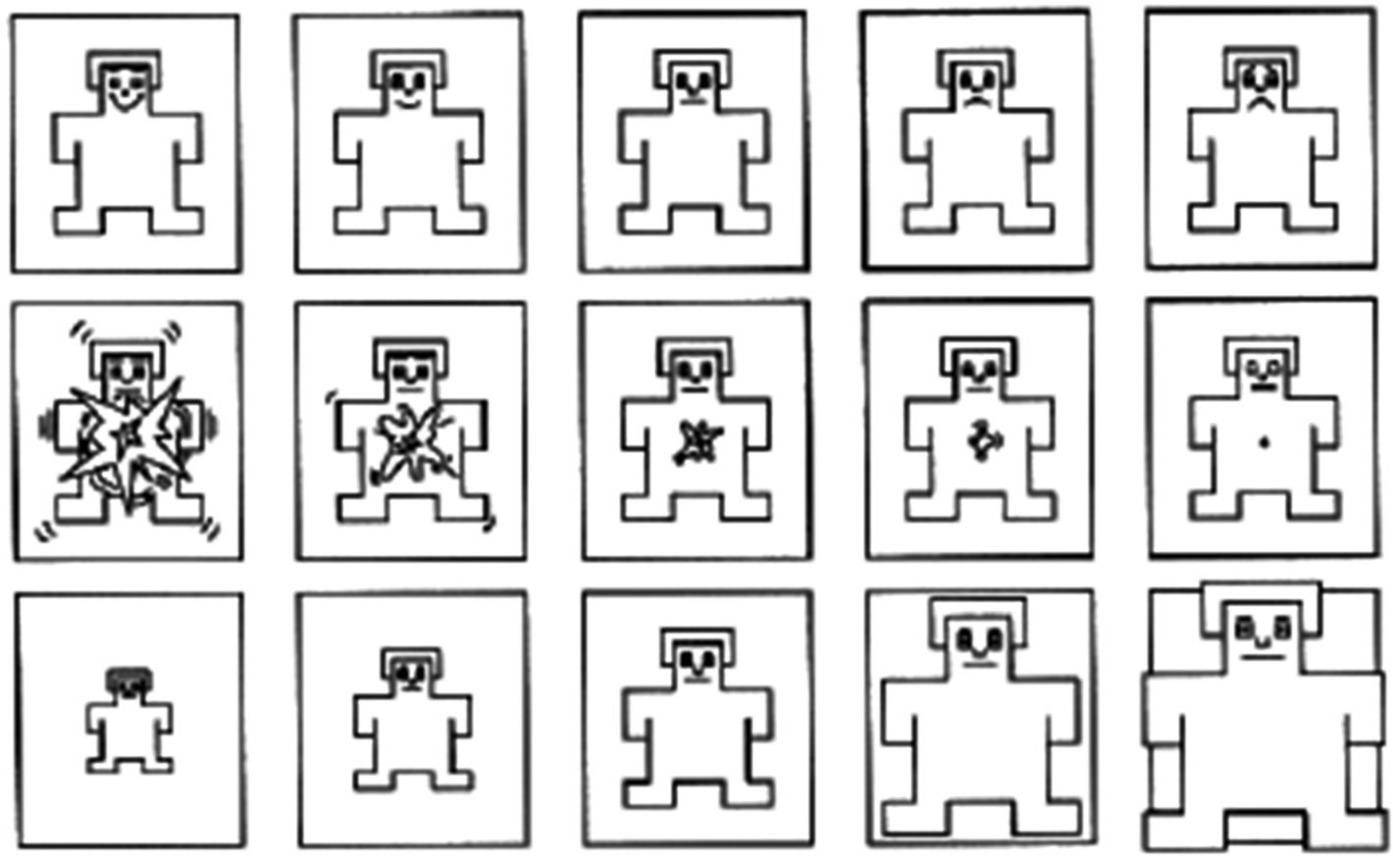
**The Self-Assessment Manikin (SAM) used to rate the affective dimensions of valence (top panel), arousal (middle panel), and dominance (bottom panel).**

The IAPS stimuli have been mainly evaluated using the "Self-Assessment Manikin" (SAM) system. SAM was developed as a non-verbal pictorial assessment technique consisting of a graphic figure depicting a 9-point scale of valence and arousal [13]. SAM ranges from a smiling, happy figure to a frowning, unhappy figure when representing the pleasure dimension; from an excited, wide-eyed figure to a relaxed, sleepy figure for the arousal dimension. The dominance dimension represents changes in control with changes in the size of SAM: a large figure indicate maximum control in the situation. Participants were asked to place an "X" over any of the five figures in each scale, or between any two figures, which resulted in a 9-point rating scale for each dimension (Figure 1).

SAM may be used to rate emotional responses to a variety of stimuli for several types of experimental subjects, since it is an easy method of reporting affective experiences. In addition, it is considered a reliable and valid instrument because ratings of pleasure and arousal using these scales are highly correlated to measures of affective evaluations [14,15]. All participants rated valence and arousal items using SAM on computers.

### Statistical analysis

Statistical analysis was performed using SPSS 17.0 (IBM, Armonk, NY, USA). Two-sample *t*-test was used to compare the differences in the scores assigned to each picture by the Chinese and American groups. The significance threshold was set at P-value of 6 × 10^-5^ (0.05/816).

One aim of the present study was to compare the differences in amounts of positive pictures rated between the two groups. We categorized valence scores ≥5 as non-negative reactions, and those <5 as negative. The chi-square test was applied to compare the overall valence subtypes assigned by Chinese and American participants. The significance was set at P-value of 6 × 10^-5^ (0.05/816).

## Results and discussion

### Results

#### Valence scores

The valence scores assigned by Chinese female participants to 31.74% (259/816) of the pictures differed from those assigned by their American counterparts (P < 6 × 10^-5^). Chinese females showed significantly lower valence scores in 194 pictures than American females; in contrast, 65 pictures were scored significantly higher in Chinese females compared with American female participants.

The valence scores assigned by Chinese male participants to 20.22% (165/816) of the pictures differed from those assigned by American males (P < 6 × 10^-5^). Chinese males showed significantly lower and higher valence scores, respectively, in 130 and 35 pictures than the American male participants.

The valence scores assigned to erotic pictures differed significantly between Chinese female and American female participants (χ^2^ = 25.93, P < 6 × 10^-5^). No significant differences were observed in the remaining categorical pictures between the two countries for both genders (P > 6 × 10^-5^), as shown in Table 1.

**Table 1:** **Distribution of valence scores in male and female participants from China and America** ^**#**^

	**Female**					**Male**			
		**Valence < 5**	**Valence ≥ 5**	**χ** ^**2**^	**P**	**Valence < 5**	**Valence ≥ 5**	**χ** ^**2**^	**P**
Animal	American	42	43	0.590	0.443	35	50	1.516	0.218
	Chinese	47	38			43	42		
Facial	American	70	95	1.222	0.269	69	96	4.857	0.028
	Chinese	80	85			89	76		
Mutilation	American	46	0		1.000	45	1		1.000
	Chinese	46	0			46	0		
Erotic	American	31	69	25.930	<6 × 10^-5*^	17	83	1.149	0.700
	Chinese	67	33			15	85		
Landscape	American	10	65	3.463	0.063	8	67	1.918	0.166
	Chinese	19	56			14	61		
Violence	American	50	2	0.707	0.400	48	4	0.915	0.329
	Chinese	48	4			45	7		
Object	American	34	87	3.615	0.057	46	75	0.838	0.36
	Chinese	48	73			53	68		
Sport	American	9	62	0.233	0.629	5	66	0.364	0.546
	Chinese	11	60			7	64		
Pollution	American	99	2	0.205	0.651	99	2	0.687	0.407
	Chinese	98	3			97	4		

^#^The data about American participants were collected by Lang.

*P < 6 × 10^-5^.

#### Arousal scores

The arousal scores assigned by female Chinese participants to 12.38% (101/816) of the pictures differed from those attributed by American females (P < 6 × 10^-5^). Chinese female participants showed significantly lower and higher arousal scores, respectively, in 46 and 55 pictures, compared with American females.

The arousal scores attributed by male Chinese participants to 15.93% (130/816) of the pictures differed from those assigned by their American counterparts (P < 6 × 10^-5^). Chinese males showed significantly lower and higher arousal scores, respectively, in 23 and 107 pictures, compared with American male participants.

Additional file 2: Table S1, Additional file 3: Table S2, Additional file 4: Table S3, Additional file 5: Table S4 summarize the detailed Chinese IAPS data.

## Discussion

In order to assess the differences in affective reactions between young Chinese and American adults, undergraduate students of these countries underwent an evaluation using IAPS pictures. Self-reported responses of participants were assessed individually, and valence and arousal scores assigned to a large proportion of pictures differed between American and Chinese, both in male and female participants. In addition, the valence scores assigned to erotic pictures differed significantly between Chinese and American female participants.

Chinese females demonstrated higher arousal reactions corresponding to facial pictures in IAPS. They more frequently reported negative reactions to erotic pictures, which induced positive reactions in American females. This difference in attitude to sexuality may be associated with differences in sex culture and education between both countries. Indeed, research suggests that sex education in China is lagging behind and compares to the situation in western countries in the 1970s; indeed, nudity was then more likely to be regarded as offensive [16]. In the Erotic category, the pictures that received the highest valence scores by Chinese female participants were weddings and non-nude romantic pictures. However, American females commonly rated pictures featuring nudity or male genitals with highest valence scores. These pictures were more likely to induce negative affective reactions in Chinese females. These results corroborate several previous cross-cultural studies. A study assessing 48 nations indicated that human mating strategies vary geographically [17]. In addition, a 37-country study reported that non-Western societies (China, India, Indonesia, Iran, Taiwan and Palestinian Arabs) attribute a higher value to chastity in a potential mate compared with Western European societies (Sweden, Norway, Finland, the Netherlands, Germany and France), attaching little importance to prior sexual experience [18]. We conclude that the cultural background, therefore, directly influences the affective reaction generated in response to sex stimulus. The IAPS images categorized as Erotic have been widely applied in neuro-imaging studies of positive emotions in American participants [19-22], but our findings indicate that these pictures could not be applied in the same way in Chinese women.

Own-race bias, also known as the cross-race effect, describes the tendency for people of one race to have difficulty recognizing and processing face and facial expressions of members of a race or ethnic group other than theirs [23]. A recent study have reported a cross-race effect in which Caucasian and Asian participants appeared to more quickly recognize faces of their own race than those of another [24]. Here, both female and male Chinese participants reported different arousal reactions compared with their American counterparts. The facial pictures included in the IAPS, mostly Western or non-Eastern faces, were assigned higher arousal scores by Chinese participants than American participants, regardless of their gender. These findings are consistent with a previous study [24], indicating the impact of the cross-race effect. It has been hypothesized that the cross-race effect is associated with limited experience of other races. However, attempts to establish this have reported contradictory results [25-27].

There were some differences between males and females in our study, although we did not directly compared gender data. Accordingly, women were shown to have a broad disposition to respond with greater defensive reactivity to aversive pictures, whereas increased appetitive activation was apparent for men only when viewing erotica.

The general pattern in the data of the present study may suggest that the differences we observed between Chinese and American participants in valence were in fact due to the Chinese indicating a lesser valence. However, Chinese tended to rate the IAPS images with greater arousal, especially men. The variations observed here may be attributed to cultural differences between China and USA. However, our study was conducted only in China and only in a single university, not including young adults enrolled at universities in other cities, those not attending university or those of older age, whose data may differ significantly from these values. Indeed, a previous Finnish study showed that age and gender influenced valence and arousal ratings [28]. In addition, geographic/cultural differences have been observed by previous studies [1-9,29]. A study using music as stimuli have suggested that emotional valence might be associated with the cultural background, while arousal might involve more basic and universal response [30]. More detailed demographic information may provide a deeper understanding of emotional differences in various cultural backgrounds. Nevertheless, our findings indicate that the IAPS norms require modifications for their appropriate application in Asian cultures.

Limitations of this study include the statistical approach. Hundreds of one-sample *t*-test could increase Type I errors, but the P-value for significance was adjusted to 6 × 10^-5^. Secondly, using simple self-reported measures of valence and arousal without psycho-physiological measures to provide additional information might still induce potential measurement error, although participants were well trained to use SAM. Thirdly, the dichotomization of valence scores may not reflect the information concerning neutral pictures. Finally, compared with Lang's study, we applied more strict inclusion or exclusion criteria, which could affect the assessment results. We acknowledge that the different exclusion criteria between the present study and the one by Lang may affect the results, and that the results may overestimate the real cultural differences between the participants of the two countries. In regard with the present study's results, we cannot ignore the effects of excluding smokers and those with psychiatric disorders. Further studies might be warranted to elucidate this issue.

## Conclusions

In summary, Chinese young adults display different affective reactions compared with their American counterparts in response to a large proportion of pictures in the IAPS. In particular, Chinese women responded significantly less positively to erotic images than American women. Therefore, IAPS requires modifications for its appropriate application in Asian cultures.

## Additional files

Additional file 1:
**Questionnaire.**
Additional file 2: Table S1. Valence scores of Chinese and American female participants. Additional file 3: Table S2. Arousal scores of Chinese and American female participants. Additional file 4: Table S3. Valence scores of Chinese and American male participants. Additional file 5: Table S4. Arousal scores of Chinese and American male participants.

## Abbreviations

IAPS: International Affective Picture System
SAM: Self-Assessment Manikin
