# Supplementary material for: Affective reactions differ between Chinese and American healthy young adults: a cross-cultural study using the international affective picture system
Source: BMC Psychiatry. 2015 Mar 27;15:60. doi: 10.1186/s12888-015-0442-9 (PMC4378560; doi:10.1186/s12888-015-0442-9)
Supplement: Additional file 1: — Questionnaire. [file 12888_2015_442_MOESM1_ESM.doc]

**Questionnaire**

1. Are you willing to participate in this study and complete the following questionnaire?

Yes ­­­­­­­__________ No­­­­­­­­­­­___________

2. Initials:­­­­­­­____________ 3. Date of birth: _______________

4. Gender: ______________ 5. Birth place: _______________

6. Years of education: ____________

7. Have you been diagnosed with affective disorders or other mental illnesses by professional institutions within the past 3 months? Yes ­­­­­­­__________ No­­­­­­­­­­­___________

8. If yes, the diseases are ________________, and the treatments are ______________________.

9. Did you have sleep problems in the last 3 months? Yes ­­­­­­­__________ No­­­­­­­­­­­___________

10. If yes, your sleep problems mainly manifested as ______________________________.

11. When you are doing the following tasks, which hand (left or right) you prefer to use?

Writing ______ Holding chopsticks _______ Throwing things ________ Brushing teeth _________ Holding scissors _________ Striking a match _______ Threading a needle _______ Holding a hammer ________ Holding a racket ________ Washing face________

12. Do you smoke? Yes ­­­­­­­__________ No­­­­­­­­­­­___________

13. If yes, your smoking frequency is ­­______ cigarettes /day

14. Do you drink alcohol? Yes ­­­­­­­__________ No­­­­­­­­­­­___________

15. If yes, your daily ­­­_________ (type of alcohol) consumption is _______ g (kg)

16. Do you have a history of long-term drug treatment? Yes ­­­­­­­__________ No­­­­­­­­­___________

17. If yes, you take __________ (names of drugs) to treat ­­­­­­­­____________ (names of diseases).

18. Have you ever been diagnosed with physical diseases? Yes ­­­­­­­__________ No­­­­­­­­­­­___________

19. If yes, the names of physical diseases are ________, and the disease duration was ________.

First Affiliated Hospital, College of Medicine, Zhejiang University
