# Supplementary material for: Affective reactions differ between Chinese and American healthy young adults: a cross-cultural study using the international affective picture system
Source: BMC Psychiatry. 2015 Mar 27;15:60. doi: 10.1186/s12888-015-0442-9 (PMC4378560; doi:10.1186/s12888-015-0442-9)
Supplement: Additional file 2: Table S1. — Valence scores of Chinese and American female participants. [file 12888_2015_442_MOESM2_ESM.doc]

Table S1. Valence scores of Chinese and American female participants

| No. | description | China | | America^#^ | | t | p |
| --- | --- | --- | --- | --- | --- | --- | --- |
|  |  | mean | SD | mean | SD |  |  |
| 1019 | Snake | 2.91 | 1.49 | 3.46 | 2.03 | -1.56 | 1.21E-01 |
| 1022 | Snake | 3.17 | 1.37 | 4.12 | 2.28 | -2.57 | 1.17E-02 |
| 1030 | Snake | 2.89 | 1.24 | 3.9 | 2.08 | -3.02 | 3.25E-03 |
| 1040 | Snake | 2.62 | 1.17 | 3.59 | 2.12 | -2.9 | 4.51E-03 |
| 1050 | Snake | 2.94 | 1.64 | 3.02 | 1.93 | -0.23 | 8.15E-01 |
| 1051 | Snake | 2.81 | 1.15 | 3.28 | 1.66 | -1.68 | 9.57E-02 |
| 1052 | Snake | 3.04 | 1.37 | 2.99 | 1.85 | 0.16 | 8.71E-01 |
| 1070 | Snake | 2.77 | 1.18 | 3.49 | 2.09 | -2.18 | 3.17E-02 |
| 1080 | Snake | 2.66 | 1.09 | 3.59 | 1.59 | -3.48 | 7.37E-04 |
| 1090 | Snake | 3 | 1.25 | 3.29 | 1.91 | -0.92 | 3.61E-01 |
| 1101 | Snake | 2.68 | 1.14 | 3.52 | 1.85 | -2.79 | 6.38E-03 |
| 1110 | Snake | 3.13 | 1.26 | 3.65 | 1.76 | -1.74 | 8.51E-02 |
| 1111 | Snake | 1.85 | 0.86 | 2.81 | 1.64 | -3.75 | 2.98E-04 |
| 1112 | Snake | 4.6 | 1.23 | 4.6 | 1.61 | -0.02 | 9.88E-01 |
| 1113 | Snake | 2.6 | 1.94 | 3.26 | 1.63 | -1.86 | 6.53E-02 |
| 1114 | Snake | 3 | 1.29 | 3.43 | 2.16 | -1.24 | 2.19E-01 |
| 1120 | Snake | 3.17 | 1.58 | 3.03 | 1.74 | 0.43 | 6.70E-01 |
| **1121** | **Lizard** | **4.83** | **1.56** | **2.65** | **1.79** | **6.59** | **2.03E-09^*^** |
| 1200 | Spider | 3.45 | 1.28 | 3.44 | 2.21 | 0.03 | 9.77E-01 |
| 1201 | Spider | 2.7 | 1.27 | 2.93 | 1.81 | -0.74 | 4.59E-01 |
| 1205 | Spider | 3.6 | 1.83 | 3.22 | 1.62 | 1.1 | 2.73E-01 |
| 1220 | Spider | 3.43 | 1.23 | 3.05 | 1.81 | 1.24 | 2.19E-01 |
| 1230 | Spider | 4.96 | 1.49 | 4.31 | 1.83 | 1.98 | 5.07E-02 |
| 1240 | Spider | 3.91 | 1.27 | 3.72 | 1.96 | 0.59 | 5.58E-01 |
| 1270 | Roach | 2.87 | 1.3 | 3.06 | 1.74 | -0.62 | 5.34E-01 |
| 1274 | Roaches | 2.83 | 1.4 | 2.86 | 1.55 | -0.1 | 9.18E-01 |
| 1275 | Roaches | 3.15 | 1.35 | 3.06 | 1.68 | 0.29 | 7.70E-01 |
| 1280 | Rat | 2.68 | 1.25 | 3.16 | 1.6 | -1.7 | 9.27E-02 |
| 1300 | PitBull | 2.94 | 1.47 | 3.41 | 1.63 | -1.55 | 1.23E-01 |
| 1301 | Dog | 3.52 | 1.68 | 3.32 | 1.53 | 0.63 | 5.30E-01 |
| 1302 | Dog | 4.6 | 1.38 | 4.11 | 1.88 | 1.5 | 1.37E-01 |
| 1303 | Dog | 5.06 | 1.41 | 4.66 | 2.22 | 1.11 | 2.71E-01 |
| 1310 | Leopard | 3.53 | 1.63 | 4.05 | 1.49 | -1.69 | 9.49E-02 |
| **1313** | **Frog** | **3.43** | **1.07** | **5.76** | **1.58** | **-8.8** | **4.00E-14^*^** |
| 1321 | Bear | 4.15 | 1.59 | 3.9 | 1.86 | 0.73 | 4.64E-01 |
| 1333 | Parrots | 5.43 | 1.73 | 6.41 | 1.68 | -2.93 | 4.21E-03 |
| **1340** | **Women** | **6.02** | **1.03** | **7.63** | **1.52** | **-6.32** | **7.12E-09^*^** |
| 1390 | Bees | 4.09 | 1.35 | 4.09 | 1.56 | -0.02 | 9.86E-01 |
| **1419** | **Bird** | **5.72** | **1.26** | **6.96** | **1.58** | **-4.4** | **2.70E-05^*^** |
| **1440** | **Seal** | **6.51** | **1.43** | **8.43** | **1.44** | **-6.79** | **7.95E-10^*^** |
| 1450 | Gannet | 6.66 | 1.15 | 6.87 | 1.54 | -0.79 | 4.31E-01 |
| **1460** | **Kitten** | **6.17** | **1.03** | **8.58** | **0.76** | **-13.45** | **3.07E-24^*^** |
| 1463 | Kittens | 7.19 | 1.24 | 7.81 | 1.96 | -1.92 | 5.71E-02 |
| **1500** | **Dog** | **5.38** | **1.07** | **7.72** | **1.7** | **-8.41** | **2.77E-13^*^** |
| **1510** | **Dog** | **4.28** | **1.58** | **7.15** | **2.33** | **-7.35** | **5.20E-11^*^** |
| 1525 | AttackDog | 3.23 | 1.03 | 2.67 | 1.74 | 2.02 | 4.63E-02 |
| **1540** | **Cat** | **5.57** | **1.39** | **7.49** | **2.02** | **-5.65** | **1.51E-07^*^** |
| 1560 | Hawk | 4.77 | 1.13 | 5.41 | 2.21 | -1.88 | 6.32E-02 |
| **1590** | **Horse** | **5.38** | **1.11** | **7.6** | **1.43** | **-8.81** | **3.81E-14^*^** |
| **1600** | **Horse** | **5.38** | **1.03** | **7.8** | **1.44** | **-9.83** | **2.12E-16^*^** |
| 1601 | Giraffes | 6.17 | 1.01 | 7.17 | 1.5 | -3.99 | 1.23E-04 |
| 1602 | Butterfly | 6.57 | 1.31 | 7.08 | 1.67 | -1.71 | 9.01E-02 |
| **1603** | **Butterfly** | **6.13** | **1.26** | **7.41** | **1.2** | **-5.28** | **7.51E-07^*^** |
| **1604** | **Butterfly** | **6.43** | **1.28** | **7.62** | **1.25** | **-4.79** | **5.84E-06^*^** |
| **1610** | **Rabbit** | **7.02** | **0.97** | **8.39** | **0.91** | **-7.39** | **4.32E-11^*^** |
| 1616 | Bird | 4.15 | 1.52 | 5.14 | 1.22 | -3.64 | 4.26E-04 |
| **1620** | **Sprgbok** | **6.06** | **1.19** | **7.95** | **1.19** | **-8.06** | **1.63E-12^*^** |
| 1640 | Coyote | 5.34 | 1.07 | 6.32 | 2.45 | -2.66 | 9.19E-03 |
| **1650** | **Jaguar** | **4.7** | **1.14** | **6.49** | **2.37** | **-4.93** | **3.26E-06^*^** |
| **1660** | **Gorilla** | **4.02** | **1.45** | **6.33** | **1.84** | **-7.09** | **1.86E-10^*^** |
| 1661 | Orangutan | 5.28 | 1.05 | 6.33 | 1.68 | -3.82 | 2.28E-04 |
| **1670** | **Cow** | **5.34** | **0.94** | **7.11** | **1.72** | **-6.53** | **2.68E-09^*^** |
| **1710** | **Puppies** | **7.21** | **1.14** | **8.59** | **0.99** | **-6.54** | **2.53E-09^*^** |
| **1720** | **Lion** | **5.28** | **1.26** | **6.78** | **1.59** | **-5.33** | **6.04E-07^*^** |
| **1721** | **Lion** | **5.55** | **1.38** | **7.7** | **1.28** | **-8.17** | **9.12E-13^*^** |
| **1722** | **Jaguars** | **5.57** | **1.46** | **7.18** | **2.3** | **-4.26** | **4.62E-05^*^** |
| 1726 | Tiger | 4.22 | 1.26 | 4.34 | 2.13 | -0.36 | 7.20E-01 |
| 1731 | Lion | 6.74 | 1.31 | 7.27 | 1.65 | -1.8 | 7.52E-02 |
| **1740** | **Owl** | **5.17** | **1.24** | **7** | **1.32** | **-7.26** | **8.28E-11^*^** |
| **1750** | **Bunnies** | **6.47** | **1** | **8.59** | **0.75** | **-12.16** | **1.71E-21^*^** |
| **1810** | **Hippo** | **5.28** | **1.81** | **6.68** | **1.56** | **-4.2** | **5.80E-05^*^** |
| **1811** | **Monkies** | **6.04** | **2.28** | **7.95** | **1.51** | **-4.97** | **2.69E-06^*^** |
| **1812** | **Elephants** | **5.85** | **1.12** | **7.22** | **1.25** | **-5.86** | **5.96E-08^*^** |
| 1850 | Camels | 5.62 | 0.92 | 6.17 | 1.61 | -2.15 | 3.36E-02 |
| 1900 | Fish | 6.47 | 1.16 | 6.83 | 1.88 | -1.18 | 2.39E-01 |
| **1910** | **Grouper** | **5.51** | **1.33** | **6.89** | **1.75** | **-4.52** | **1.67E-05^*^** |
| **1920** | **Porpoise** | **6.83** | **0.99** | **7.94** | **1.61** | **-4.25** | **4.80E-05^*^** |
| 1930 | Shark | 4 | 1.1 | 3.56 | 1.9 | 1.45 | 1.51E-01 |
| 1931 | Shark | 4.45 | 1.7 | 3.57 | 2.13 | 2.32 | 2.21E-02 |
| 1932 | Shark | 3.53 | 1.23 | 2.92 | 1.87 | 1.97 | 5.20E-02 |
| 1935 | HermitCrab | 4.51 | 1.25 | 4.63 | 1.5 | -0.44 | 6.59E-01 |
| 1942 | Tuetle | 5.11 | 1.75 | 6.34 | 1.93 | -3.41 | 9.49E-04 |
| **1945** | **Tuetles** | **2.85** | **1.33** | **4.22** | **1.67** | **-4.61** | **1.19E-05^*^** |
| 1947 | Octopus | 5.67 | 1.52 | 5.68 | 2.1 | -0.02 | 9.87E-01 |
| **1999** | **Mickey** | **6.11** | **1.7** | **7.68** | **1.52** | **-4.94** | **3.14E-06^*^** |
| 2000 | Adult | 6.67 | 1.17 | 7.1 | 1.62 | -1.55 | 1.24E-01 |
| 2005 | AttractiveMan | 6.15 | 0.87 | 6.76 | 2.01 | -2.02 | 4.61E-02 |
| **2010** | **Adult** | **5.31** | **1.29** | **6.67** | **1.86** | **-4.33** | **3.57E-05^*^** |
| 2020 | Adult | 5.02 | 1.39 | 5.97 | 2.13 | -2.69 | 8.31E-03 |
| 2025 | Women | 5.28 | 1.36 | 5.44 | 1.22 | -0.64 | 5.24E-01 |
| 2030 | Women | 6.23 | 1.35 | 6.02 | 1.48 | 0.75 | 4.53E-01 |
| **2040** | **Baby** | **6.89** | **1.24** | **8.74** | **0.64** | **-9.43** | **1.60E-15^*^** |
| **2050** | **Baby** | **7.13** | **2.32** | **8.65** | **0.85** | **-4.38** | **2.95E-05^*^** |
| 2053 | Baby | 2.81 | 1.78 | 2.17 | 1.9 | 1.77 | 8.06E-02 |
| 2055.1 | ManInPool | 2.77 | 1.24 | 2.84 | 1.89 | -0.24 | 8.14E-01 |
| 2057 | Father | 7.81 | 1.32 | 8.39 | 0.94 | -2.55 | 1.22E-02 |
| **2058** | **Baby** | **6.66** | **1.03** | **8.24** | **1.07** | **-7.65** | **1.23E-11^*^** |
| 2070 | Baby | 7.55 | 1.19 | 8.5 | 1.28 | -3.88 | 1.84E-04 |
| 2071 | Baby | 7.47 | 1.1 | 8.21 | 1.29 | -3.14 | 2.23E-03 |
| **2080** | **Babies** | **7.04** | **1.12** | **8.46** | **1.2** | **-6.2** | **1.26E-08^*^** |
| **2091** | **Girls** | **7.06** | **1.24** | **8.26** | **1.17** | **-5.03** | **2.16E-06^*^** |
| 2092 | Clowns | 6.15 | 0.93 | 6.7 | 2.05 | -1.77 | 7.99E-02 |
| **2095** | **Toddler** | **2.91** | **1.7** | **1.48** | **0.97** | **5.21** | **1.01E-06^*^** |
| 2100 | AngryFace | 2.6 | 1.36 | 3.37 | 1.94 | -2.34 | 2.12E-02 |
| 2110 | AngryFace | 2.94 | 1.36 | 3.44 | 1.97 | -1.51 | 1.35E-01 |
| 2120 | AngryFace | 2.63 | 1.59 | 3.03 | 1.74 | -1.23 | 2.20E-01 |
| 2130 | Women | 3.1 | 1.39 | 3.98 | 1.23 | -3.38 | 1.02E-03 |
| 2141 | GrievingFem | 3.13 | 1.48 | 2.27 | 1.76 | 2.68 | 8.65E-03 |
| **2150** | **Baby** | **6.28** | **1.17** | **8.31** | **1.49** | **-7.72** | **8.68E-12^*^** |
| **2160** | **Father** | **6.77** | **1.53** | **8.16** | **1.28** | **-4.98** | **2.64E-06^*^** |
| **2165** | **Father** | **7.26** | **1.17** | **8.29** | **1.17** | **-4.49** | **1.91E-05^*^** |
| **2170** | **Mother** | **6.32** | **1.16** | **7.69** | **1.39** | **-5.44** | **3.69E-07^*^** |
| 2190 | Man | 4.96 | 0.51 | 4.9 | 1.31 | 0.29 | 7.69E-01 |
| 2191 | Farmer | 5.11 | 1.55 | 5.14 | 1.71 | -0.11 | 9.16E-01 |
| 2200 | NeutFace | 5.55 | 1.47 | 4.88 | 1.42 | 2.36 | 2.02E-02 |
| **2205** | **Hospital** | **3.66** | **1.11** | **1.65** | **1.05** | **9.43** | **1.59E-15^*^** |
| 2206 | Fingerprint | 4.51 | 0.86 | 4.2 | 1.3 | 1.44 | 1.54E-01 |
| **2208** | **Bride** | **6.28** | **1.06** | **7.55** | **1.64** | **-4.71** | **7.90E-06^*^** |
| **2209** | **Bride** | **6.19** | **1.23** | **7.95** | **1.46** | **-6.63** | **1.67E-09^*^** |
| 2210 | NeutFace | 4.48 | 1.43 | 4.36 | 1.91 | 0.36 | 7.20E-01 |
| 2214 | NeutFace | 5.51 | 1.78 | 5.11 | 1.19 | 1.33 | 1.86E-01 |
| 2215 | NeutFace | 5 | 1 | 4.53 | 1.37 | 2 | 4.85E-02 |
| **2216** | **Chidren** | **6.3** | **1.1** | **7.85** | **1.18** | **-6.91** | **4.43E-10^*^** |
| **2220** | **MaleFace** | **3.68** | **1.37** | **4.95** | **1.56** | **-4.39** | **2.74E-05^*^** |
| 2221 | Judge | 4.67 | 1.15 | 4.33 | 1.24 | 1.44 | 1.53E-01 |
| **2222** | **BoysReading** | **6.19** | **1.06** | **7.59** | **1.54** | **-5.41** | **4.27E-07^*^** |
| **2224** | **Boys** | **6.3** | **0.88** | **7.63** | **1.65** | **-5.15** | **1.28E-06^*^** |
| 2230 | SadFace | 4.64 | 1.01 | 4.41 | 1.33 | 0.98 | 3.28E-01 |
| 2235 | Butcher | 5.32 | 0.93 | 5.79 | 1.35 | -2.06 | 4.15E-02 |
| 2240 | NeutChild | 6.53 | 1.33 | 7.08 | 1.35 | -2.08 | 4.03E-02 |
| **2250** | **NeutBaby** | **5.89** | **1.05** | **7.53** | **1.78** | **-5.73** | **1.06E-07^*^** |
| **2260** | **NeutBaby** | **5.98** | **1.5** | **8.51** | **1.05** | **-9.88** | **1.62E-16^*^** |
| **2270** | **NeutChild** | **5.27** | **1.45** | **6.95** | **1.66** | **-5.47** | **3.25E-07^*^** |
| 2271 | Women | 3.26 | 1.19 | 4.11 | 1.37 | -3.39 | 1.00E-03 |
| 2272 | LonelyBoy | 4.98 | 1.22 | 4.49 | 2.06 | 1.47 | 1.44E-01 |
| 2276 | Girl | 3.55 | 1.53 | 3.34 | 1.75 | 0.66 | 5.12E-01 |
| 2278 | Kids | 4.02 | 1.41 | 3.34 | 1.75 | 2.18 | 3.18E-02 |
| 2280 | Boy | 5.13 | 1.13 | 3.97 | 1.73 | 4.04 | 1.05E-04 |
| **2299** | **Family** | **5.85** | **1.08** | **7.76** | **1.51** | **-7.4** | **4.06E-11^*^** |
| 2303 | Children | 6.09 | 1.06 | 7.03 | 2.2 | -2.8 | 6.09E-03 |
| **2304** | **Girl** | **5.96** | **1.44** | **7.7** | **1.11** | **-6.84** | **6.21E-10^*^** |
| 2310 | Mother | 6.53 | 0.95 | 7.37 | 1.63 | -3.22 | 1.74E-03 |
| **2311** | **Mother** | **6.81** | **0.95** | **7.82** | **1.21** | **-4.74** | **6.98E-06^*^** |
| 2312 | Mother | 4.17 | 1.35 | 3.51 | 1.8 | 2.12 | 3.62E-02 |
| 2320 | Girl | 6.33 | 1.33 | 6.82 | 1.37 | -1.84 | 6.81E-02 |
| 2331 | Chef | 6.77 | 1.29 | 7.69 | 1.58 | -3.26 | 1.51E-03 |
| **2340** | **Family** | **6.34** | **1.4** | **8.34** | **1.1** | **-8.02** | **1.98E-12^*^** |
| 2341 | Children | 7.15 | 1 | 7.82 | 1.57 | -2.61 | 1.05E-02 |
| **2344** | **Children** | **5.7** | **1.2** | **7.18** | **1.97** | **-4.63** | **1.08E-05^*^** |
| **2345** | **Children** | **5.7** | **2.27** | **7.75** | **1.73** | **-5.15** | **1.31E-06^*^** |
| **2346** | **Kids** | **5.62** | **1.41** | **7.01** | **1.69** | **-4.56** | **1.46E-05^*^** |
| 2351 | NursingBaby | 4.77 | 1.49 | 5.49 | 2.17 | -1.97 | 5.13E-02 |
| **2352** | **Kiss** | **5.32** | **1.71** | **7.38** | **1.93** | **-5.75** | **9.68E-08^*^** |
| **2352.1** | **Kiss** | **5.68** | **1.42** | **7.57** | **1.72** | **-6.1** | **1.93E-08^*^** |
| 2352.2 | BloodyKiss | 1.7 | 1.2 | 1.87 | 1.41 | -0.65 | 5.15E-01 |
| **2357** | **Man** | **4.19** | **1.24** | **5.32** | **1.14** | **-4.79** | **5.74E-06^*^** |
| **2360** | **Family** | **6.6** | **1.06** | **8.2** | **1.59** | **-6.06** | **2.32E-08^*^** |
| **2370** | **ThreeMan** | **6.15** | **0.88** | **7.43** | **1.49** | **-5.35** | **5.60E-07^*^** |
| 2372 | Woman | 4.63 | 1.16 | 5.55 | 1.71 | -3.21 | 1.78E-03 |
| **2375.1** | **Woman** | **3.38** | **1.07** | **1.91** | **1.19** | **6.59** | **2.04E-09^*^** |
| 2381 | Girl | 4.31 | 1.87 | 5.03 | 1.34 | -2.23 | 2.80E-02 |
| 2383 | Secretary | 4.51 | 1.12 | 4.79 | 1.44 | -1.11 | 2.72E-01 |
| **2385** | **Girl** | **3.81** | **1.5** | **5.15** | **1.24** | **-4.93** | **3.17E-06^*^** |
| **2387** | **Kids** | **6.53** | **1.14** | **7.68** | **1.59** | **-4.23** | **5.13E-05^*^** |
| **2388** | **Kids** | **6.13** | **1.54** | **8.1** | **1.15** | **-7.32** | **6.02E-11^*^** |
| **2389** | **Teens** | **5.17** | **1.07** | **6.76** | **1.92** | **-5.23** | **9.15E-07^*^** |
| 2391 | Boy | 6.62 | 1.01 | 6.9 | 1.66 | -1.05 | 2.95E-01 |
| 2393 | Factoryworker | 4.79 | 0.93 | 4.92 | 1.05 | -0.68 | 4.98E-01 |
| 2394 | Medicalworker | 5.04 | 1.16 | 6.14 | 2 | -3.43 | 8.75E-04 |
| **2395** | **Family** | **6.17** | **0.96** | **8.31** | **1.12** | **-10.42** | **1.06E-17^*^** |
| 2399 | Woman | 3.51 | 1.08 | 3.5 | 1.56 | 0.04 | 9.68E-01 |
| 2410 | Boy | 5.39 | 1.39 | 4.54 | 1.94 | 2.57 | 1.16E-02 |
| **2435** | **Mom/Son** | **4.87** | **1.13** | **5.96** | **1.3** | **-4.53** | **1.62E-05^*^** |
| 2440 | NeutGirl | 4.67 | 1.37 | 4.54 | 0.99 | 0.53 | 5.96E-01 |
| 2441 | NeutralGirl | 4.47 | 1.21 | 4.35 | 1.51 | 0.44 | 6.62E-01 |
| 2442 | DryingHair | 6.26 | 1.19 | 6.51 | 1.55 | -0.94 | 3.50E-01 |
| **2455** | **SadGirls** | **4.07** | **1.29** | **2.63** | **1.95** | **4.44** | **2.27E-05^*^** |
| 2480 | ElderlyMan | 5.75 | 1.59 | 4.77 | 1.49 | 3.22 | 1.71E-03 |
| **2485** | **Man** | **4.64** | **1.15** | **5.91** | **1.3** | **-5.27** | **7.91E-07^*^** |
| 2487 | Musician | 5.47 | 0.8 | 5.28 | 2.03 | 0.63 | 5.29E-01 |
| **2490** | **Man** | **4** | **1.02** | **2.74** | **1.51** | **4.99** | **2.57E-06^*^** |
| 2491 | SickMan | 4.64 | 1.09 | 3.96 | 1.54 | 2.6 | 1.08E-02 |
| 2493 | NeutralMale | 5.66 | 1.03 | 5.1 | 1.37 | 2.35 | 2.05E-02 |
| 2495 | Man | 4.91 | 0.97 | 5.14 | 1.22 | -1.04 | 3.02E-01 |
| 2499 | NeutralMale | 4.21 | 1.32 | 5.35 | 1.73 | -3.77 | 2.76E-04 |
| 2500 | Man | 6.66 | 1.11 | 6.44 | 1.49 | 0.85 | 3.98E-01 |
| 2501 | Couple | 6.61 | 1.08 | 7.45 | 1.52 | -3.25 | 1.58E-03 |
| 2510 | ElderlyWoman | 6.21 | 1.18 | 7.13 | 1.9 | -2.97 | 3.69E-03 |
| 2514 | Woman | 5.06 | 1.11 | 5.21 | 1.22 | -0.64 | 5.26E-01 |
| 2515 | Harvest | 5.51 | 1.44 | 6.31 | 1.56 | -2.7 | 8.03E-03 |
| 2516 | ElderlyWoman | 5.38 | 1.13 | 4.76 | 1.66 | 2.22 | 2.83E-02 |
| 2518 | Quilting | 5.43 | 1.04 | 5.85 | 1.71 | -1.54 | 1.28E-01 |
| 2520 | ElderlyMan | 4.68 | 1.24 | 4.14 | 1.85 | 1.75 | 8.29E-02 |
| **2530** | **Couple** | **6.53** | **0.91** | **8.25** | **1.1** | **-8.68** | **7.27E-14^*^** |
| 2540 | Mother | 7.32 | 1.42 | 7.95 | 1.39 | -2.28 | 2.46E-02 |
| **2550** | **Couple** | **6.72** | **1.21** | **8.14** | **1.53** | **-5.24** | **8.82E-07^*^** |
| **2560** | **Picnic** | **5.49** | **0.91** | **6.64** | **1.46** | **-4.84** | **4.76E-06^*^** |
| 2570 | Man | 4.56 | 1.35 | 4.95 | 1.09 | -1.6 | 1.14E-01 |
| 2575 | Propeller | 4.64 | 0.94 | 5.32 | 1.14 | -3.32 | 1.27E-03 |
| 2579 | Bakers | 5.45 | 1.06 | 5.39 | 1.46 | 0.22 | 8.23E-01 |
| 2580 | Chess | 5.35 | 1.38 | 5.9 | 1.44 | -1.98 | 5.01E-02 |
| 2590 | ElderlyWoman | 4.43 | 1.3 | 3.46 | 2.24 | 2.71 | 7.94E-03 |
| 2595 | Women | 4.62 | 0.85 | 4.92 | 1.31 | -1.4 | 1.64E-01 |
| 2600 | Beer | 4.98 | 1.06 | 5.77 | 1.62 | -2.95 | 3.99E-03 |
| **2616** | **Dancer** | **4.09** | **1.56** | **5.85** | **2.02** | **-4.98** | **2.61E-06^*^** |
| 2620 | Woman | 6.62 | 1.24 | 6.07 | 1.8 | 1.8 | 7.44E-02 |
| **2630** | **Male** | **5.58** | **1.25** | **7.36** | **1.57** | **-6.38** | **5.31E-09^*^** |
| 2635 | Cowboy | 4.34 | 1.13 | 5.19 | 1.68 | -3.03 | 3.10E-03 |
| 2650 | Boy | 7.04 | 1.22 | 7.71 | 1.26 | -2.74 | 7.29E-03 |
| 2655 | Child | 6.81 | 1.44 | 7.06 | 2.42 | -0.65 | 5.20E-01 |
| **2660** | **Baby** | **6.28** | **0.83** | **8.18** | **1.24** | **-9.22** | **4.71E-15^*^** |
| 2661 | Baby | 2.91 | 1.49 | 4.46 | 2.72 | -3.6 | 4.90E-04 |
| 2681 | Police | 4.63 | 1.48 | 4.26 | 1.73 | 1.15 | 2.52E-01 |
| 2682 | Police | 3.69 | 1.42 | 3.48 | 1.69 | 0.68 | 5.01E-01 |
| **2683** | **War** | **3.19** | **1.06** | **1.97** | **1.64** | **4.52** | **1.68E-05^*^** |
| **2688** | **Hunters** | **4.7** | **1.82** | **2.36** | **2.06** | **6.13** | **1.72E-08^*^** |
| 2690 | Terrorist | 3.43 | 1.54 | 4.57 | 1.5 | -3.82 | 2.34E-04 |
| **2691** | **Riot** | **3.53** | **1.02** | **2.3** | **1.27** | **5.45** | **3.63E-07^*^** |
| **2692** | **Bomb** | **4.17** | **1.32** | **2.77** | **1.44** | **5.15** | **1.30E-06^*^** |
| 2694 | Police | 3.45 | 1.23 | 2.97 | 1.54 | 1.75 | 8.27E-02 |
| 2695 | Refugees | 3.47 | 1.16 | 3.55 | 1.73 | -0.28 | 7.77E-01 |
| 2700 | Woman | 3.36 | 1.17 | 3.1 | 1.31 | 1.07 | 2.87E-01 |
| 2702 | BingeEating | 5.79 | 1.73 | 4.77 | 2.03 | 2.75 | 7.01E-03 |
| 2710 | DrugAddict | 3.11 | 1.26 | 2.16 | 1.49 | 3.49 | 7.09E-04 |
| 2715 | Smoking | 3.53 | 0.93 | 2.99 | 2.13 | 1.69 | 9.41E-02 |
| **2720** | **Urinating** | **3.4** | **1.73** | **5.24** | **1.62** | **-5.56** | **2.24E-07^*^** |
| 2722 | Jail | 3.28 | 1.04 | 2.94 | 1.61 | 1.27 | 2.08E-01 |
| **2730** | **NativeBoy** | **3.23** | **1.63** | **1.8** | **1.31** | **4.9** | **3.64E-06^*^** |
| 2745.1 | Shopping | 4.81 | 0.99 | 5.38 | 1.22 | -2.62 | 1.03E-02 |
| 2749 | Smoking | 4.74 | 0.92 | 4.97 | 1.53 | -0.91 | 3.64E-01 |
| 2750 | Bum | 2.79 | 1.2 | 2.55 | 1.19 | 1.03 | 3.06E-01 |
| 2751 | DrunkDriving | 3.15 | 1.3 | 2.06 | 1.47 | 3.97 | 1.36E-04 |
| 2752 | Alcoholic | 3.3 | 1.35 | 3.66 | 1.72 | -1.2 | 2.35E-01 |
| 2753 | Alcoholic | 3.58 | 1.22 | 2.67 | 1.7 | 3.15 | 2.16E-03 |
| **2780** | **Actor** | **2.38** | **1.24** | **4.78** | **2.1** | **-7.11** | **1.69E-10^*^** |
| 2791 | Balloons | 6.38 | 1.19 | 7.25 | 1.36 | -3.46 | 7.92E-04 |
| 2795 | Boy | 4.15 | 1.1 | 3.77 | 1.91 | 1.24 | 2.17E-01 |
| 2800 | SadChild | 2.31 | 1.37 | 1.41 | 0.79 | 4.06 | 9.65E-05 |
| 2810 | Boy | 4.36 | 1.41 | 4.14 | 1.58 | 0.75 | 4.53E-01 |
| 2830 | Woman | 4.02 | 1.48 | 4.48 | 1.68 | -1.48 | 1.43E-01 |
| 2840 | Chess | 5.73 | 1.42 | 4.9 | 1.23 | 3.16 | 2.07E-03 |
| 2850 | Tourist | 5.04 | 1.14 | 5.96 | 1.22 | -3.94 | 1.48E-04 |
| 2870 | Teenager | 5.45 | 1.32 | 5.41 | 1.68 | 0.12 | 9.04E-01 |
| 2880 | Shadow | 4.55 | 1.23 | 5.22 | 1.79 | -2.22 | 2.83E-02 |
| 2890 | Twins | 4.13 | 1.41 | 5.02 | 1.1 | -3.58 | 5.29E-04 |
| **2900** | **CryingBoy** | **4.22** | **1.59** | **2.16** | **1.52** | **6.71** | **1.13E-09^*^** |
| **2900.1** | **FoodBasket** | **4.13** | **1.36** | **2.14** | **1.3** | **7.57** | **1.79E-11^*^** |
| 2900.2 | DeerHead | 6.3 | 1.23 | 6.91 | 2.19 | -1.76 | 8.13E-02 |
| 2980 | Mutilation | 4.98 | 1.17 | 5.77 | 1.79 | -2.67 | 8.83E-03 |
| 2981 | Mutilation | 3 | 1.63 | 2.09 | 1.73 | 2.75 | 7.06E-03 |
| 3000 | Mutilation | 1.28 | 0.74 | 1.17 | 0.54 | 0.82 | 4.12E-01 |
| 3005.1 | OpenGrave | 1.72 | 0.88 | 1.35 | 0.85 | 2.19 | 3.07E-02 |
| 3010 | Mutilation | 1.47 | 0.95 | 1.29 | 0.82 | 1.03 | 3.07E-01 |
| 3015 | Accident | 2.26 | 1.61 | 1.34 | 0.71 | 3.7 | 3.52E-04 |
| 3022 | Scream | 3.45 | 1.41 | 3.29 | 2.02 | 0.46 | 6.49E-01 |
| 3030 | Mutilation | 2.23 | 0.94 | 1.51 | 1.07 | 3.63 | 4.42E-04 |
| 3051 | Mutilation | 1.89 | 0.94 | 2.06 | 1.96 | -0.56 | 5.79E-01 |
| 3053 | BurnVictim | 1.38 | 0.8 | 1.51 | 0.73 | -0.86 | 3.90E-01 |
| 3060 | Mutilation | 1.83 | 1.69 | 1.66 | 1.71 | 0.51 | 6.15E-01 |
| 3061 | Mutilation | 1.28 | 0.68 | 1.97 | 1.49 | -3.07 | 2.79E-03 |
| 3062 | Mutilation | 1.38 | 0.98 | 1.62 | 1.18 | -1.15 | 2.54E-01 |
| 3063 | Mutilation | 1.55 | 1.18 | 1.18 | 0.65 | 1.98 | 5.09E-02 |
| 3064 | Mutilation | 1.43 | 0.9 | 1.15 | 0.44 | 1.95 | 5.39E-02 |
| 3068 | Mutilation | 1.47 | 0.88 | 1.18 | 0.7 | 1.83 | 7.01E-02 |
| 3069 | Mutilation | 1.47 | 0.72 | 1.32 | 1.01 | 0.86 | 3.91E-01 |
| 3071 | Mutilation | 1.45 | 0.85 | 1.69 | 1.14 | -1.23 | 2.20E-01 |
| 3080 | Mutilation | 2.11 | 1.63 | 1.33 | 0.75 | 3.09 | 2.60E-03 |
| 3100 | BurnVictim | 1.51 | 0.8 | 1.35 | 0.96 | 0.92 | 3.60E-01 |
| 3102 | BurnVictim | 1.66 | 1.22 | 1.22 | 0.85 | 2.11 | 3.73E-02 |
| 3110 | BurnVictim | 1.23 | 0.52 | 1.47 | 0.89 | -1.68 | 9.56E-02 |
| 3120 | DeadBoy | 1.6 | 0.68 | 1.33 | 0.74 | 1.89 | 6.11E-02 |
| 3130 | Mutilation | 1.74 | 0.9 | 1.26 | 0.68 | 3.05 | 2.93E-03 |
| 3140 | DeadBoy | 1.81 | 1.45 | 1.5 | 0.97 | 1.26 | 2.11E-01 |
| 3150 | Mutilation | 1.7 | 1.1 | 1.98 | 1.54 | -1.07 | 2.89E-01 |
| 3160 | EyeDisease | 2.49 | 1.28 | 2.55 | 1.32 | -0.24 | 8.14E-01 |
| 3168 | Mutilation | 1.4 | 0.74 | 1.33 | 0.83 | 0.48 | 6.33E-01 |
| 3170 | BabyTumor | 1.72 | 1.54 | 1.2 | 0.57 | 2.25 | 2.69E-02 |
| **3180** | **BatteredFem** | **2.63** | **0.98** | **1.67** | **0.9** | **5.14** | **1.36E-06^*^** |
| 3181 | BatteredFem | 2.38 | 1.01 | 2.01 | 1.29 | 1.63 | 1.07E-01 |
| 3190 | Scar | 2.83 | 1.24 | 3.33 | 1.49 | -1.86 | 6.65E-02 |
| 3210 | Surgery | 4.55 | 1.04 | 4.21 | 1.97 | 1.1 | 2.72E-01 |
| 3220 | Hospital | 2.25 | 1.14 | 2.41 | 1.31 | -0.66 | 5.09E-01 |
| **3230** | **DyingMan** | **3.11** | **1.42** | **1.67** | **0.99** | **5.95** | **3.97E-08^*^** |
| **3250** | **OpenChest** | **2.34** | **1.13** | **3.67** | **1.51** | **-5.08** | **1.71E-06^*^** |
| 3261 | Tumor | 1.72 | 0.85 | 1.7 | 1.43 | 0.09 | 9.31E-01 |
| 3266 | Injury | 1.68 | 0.81 | 1.26 | 0.56 | 3.04 | 2.98E-03 |
| 3280 | DentalExam | 3.64 | 1.31 | 3.62 | 1.63 | 0.07 | 9.45E-01 |
| 3300 | DisabledChild | 3.38 | 1.26 | 2.35 | 1.3 | 4.08 | 8.91E-05 |
| **3301** | **InjuredChild** | **2.36** | **0.87** | **1.49** | **0.81** | **5.24** | **8.65E-07^*^** |
| 3350 | Infant | 2.53 | 1.23 | 1.76 | 1.72 | 2.62 | 1.00E-02 |
| 3400 | SeveredHand | 1.91 | 1.59 | 2.06 | 1.77 | -0.45 | 6.52E-01 |
| **3500** | **Attack** | **3.02** | **1.07** | **1.94** | **1.38** | **4.45** | **2.19E-05^*^** |
| 3530 | Attack | 2.19 | 0.99 | 1.51 | 1 | 3.47 | 7.75E-04 |
| 3550 | Injury | 2.04 | 1.16 | 1.98 | 1.22 | 0.26 | 7.92E-01 |
| 3550.1 | PlaneCrash | 2.62 | 1.34 | 1.92 | 1.34 | 2.63 | 9.76E-03 |
| 3550.2 | Coach | 3.85 | 1.4 | 4.71 | 1.8 | -2.71 | 7.83E-03 |
| 4000 | Artist | 4.02 | 1.47 | 4.28 | 1.39 | -0.92 | 3.60E-01 |
| 4001 | EroticFemale | 3.96 | 1.9 | 3.58 | 1.74 | 1.05 | 2.97E-01 |
| 4002 | EroticFemale | 4.24 | 1.7 | 4.14 | 1.82 | 0.29 | 7.76E-01 |
| 4003 | EroticFemale | 3.7 | 1.91 | 4.3 | 1.64 | -1.71 | 9.11E-02 |
| 4004 | EroticFemale | 4.94 | 1.54 | 4.17 | 1.53 | 2.53 | 1.28E-02 |
| 4005 | EroticFemale | 3.63 | 2.07 | 4.36 | 1.97 | -1.84 | 6.81E-02 |
| 4100 | MaleDancers | 6.33 | 1.6 | 6.7 | 1.73 | -1.13 | 2.62E-01 |
| 4141 | EroticFemale | 4.83 | 2.13 | 4.01 | 1.9 | 2.06 | 4.24E-02 |
| 4142 | EroticFemale | 4.28 | 1.8 | 3.49 | 2.18 | 2 | 4.81E-02 |
| 4150 | AttractiveFem | 5.48 | 1.5 | 5.36 | 1.44 | 0.41 | 6.80E-01 |
| 4180 | EroticFemale | 4.23 | 1.67 | 4.21 | 1.84 | 0.06 | 9.54E-01 |
| 4210 | EroticFemale | 2.89 | 1.71 | 3.13 | 1.66 | -0.71 | 4.79E-01 |
| 4220 | EroticFemale | 6.13 | 0.99 | 5.29 | 1.35 | 3.6 | 4.93E-04 |
| 4230 | Prostitute | 3 | 1.71 | 3.87 | 1.83 | -2.49 | 1.43E-02 |
| 4232 | EroticFemale | 2.89 | 1.39 | 4.06 | 2.05 | -3.41 | 9.33E-04 |
| **4233** | **Prostitute** | **5.54** | **1.69** | **3.89** | **1.7** | **4.95** | **3.03E-06^*^** |
| 4235 | EroticFemale | 3.91 | 1.78 | 3.67 | 1.82 | 0.69 | 4.93E-01 |
| 4240 | EroticFemale | 3.33 | 1.88 | 3.73 | 1.77 | -1.11 | 2.70E-01 |
| 4250 | AttractiveFem | 5.7 | 1.5 | 5.18 | 1.55 | 1.73 | 8.67E-02 |
| 4255 | EroticFemale | 4.87 | 1.57 | 4.86 | 1.54 | 0.03 | 9.74E-01 |
| 4274 | AttractiveFem | 5.15 | 1.18 | 4.49 | 1.52 | 2.47 | 1.52E-02 |
| 4275 | AttractiveFem | 4.64 | 1.86 | 4.38 | 1.4 | 0.79 | 4.30E-01 |
| **4279** | **EroticFemale** | **2.48** | **1.61** | **4.16** | **1.43** | **-5.59** | **1.94E-07^*^** |
| 4290 | EroticFemale | 2.83 | 1.47 | 3.67 | 1.6 | -2.78 | 6.55E-03 |
| 4300 | EroticFemale | 3.54 | 1.88 | 4.19 | 1.91 | -1.74 | 8.55E-02 |
| 4302 | EroticFemale | 2.35 | 1.23 | 3.3 | 1.83 | -3.11 | 2.43E-03 |
| 4310 | EroticFemale | 3.73 | 1.96 | 4.81 | 1.26 | -3.31 | 1.29E-03 |
| 4320 | EroticFemale | 3.46 | 1.7 | 4.66 | 1.21 | -4.11 | 8.00E-05 |
| **4460** | **EroticMale** | **3.65** | **2.09** | **6.29** | **1.56** | **-7.25** | **8.64E-11^*^** |
| **4470** | **EroticMale** | **3.34** | **1.67** | **6.75** | **1.43** | **-11.1** | **3.42E-19^*^** |
| **4490** | **EroticMale** | **2.79** | **1.69** | **6.27** | **1.95** | **-9.69** | **4.23E-16^*^** |
| **4500** | **AttractiveMan** | **4.96** | **2.1** | **6.9** | **2.08** | **-4.72** | **7.62E-06^*^** |
| **4503** | **EroticMale** | **4.51** | **1.35** | **6.72** | **1.49** | **-7.9** | **3.60E-12^*^** |
| **4510** | **AttractiveMan** | **4.98** | **1.38** | **7** | **2.28** | **-5.48** | **3.08E-07^*^** |
| **4520** | **EroticMale** | **4.32** | **1.68** | **6.94** | **1.34** | **-8.71** | **6.04E-14^*^** |
| **4530** | **EroticMale** | **3.34** | **1.53** | **6.19** | **1.93** | **-8.33** | **4.17E-13^*^** |
| **4531** | **EroticMale** | **4.51** | **1.72** | **6.96** | **1.52** | **-7.64** | **1.26E-11^*^** |
| **4532** | **AttractiveMan** | **6.52** | **1.28** | **7.62** | **1.2** | **-4.5** | **1.83E-05^*^** |
| **4533** | **AttractiveMan** | **5.87** | **1.39** | **7** | **2.27** | **-3.06** | **2.85E-03^*^** |
| **4534** | **MaleDancers** | **5.25** | **1.25** | **6.56** | **1.54** | **-4.76** | **6.51E-06^*^** |
| **4535** | **Weightlifter** | **5.79** | **1.15** | **7.06** | **1.58** | **-4.68** | **8.79E-06^*^** |
| 4536 | AttractiveMan | 5.44 | 1.51 | 6.58 | 1.51 | -3.83 | 2.19E-04 |
| **4537** | **AttractiveMan** | **4.15** | **1.32** | **6.44** | **1.81** | **-7.37** | **4.73E-11^*^** |
| **4538** | **EroticMale** | **4.43** | **1.26** | **7.04** | **1.74** | **-8.74** | **5.22E-14^*^** |
| **4550** | **EroticMale** | **2.88** | **1.77** | **6.22** | **1.86** | **-9.35** | **2.40E-15^*^** |
| **4561** | **EroticMale** | **3.62** | **1.68** | **6.1** | **2** | **-6.85** | **6.03E-10^*^** |
| 4571 | AttractiveMan | 6.48 | 1.17 | 6.18 | 1.38 | 1.19 | 2.37E-01 |
| **4572** | **AttractiveMan** | **5.47** | **1.27** | **7.52** | **1.37** | **-7.89** | **3.64E-12^*^** |
| 4598 | Couple | 5.89 | 1.93 | 6.13 | 3.02 | -0.48 | 6.35E-01 |
| **4599** | **Romance** | **5.83** | **1.39** | **7.23** | **1.66** | **-4.65** | **1.00E-05^*^** |
| 4601 | Romance | 7.06 | 1.46 | 6.68 | 1.33 | 1.38 | 1.72E-01 |
| 4603 | Romance | 6.42 | 1.57 | 7.58 | 1.51 | -3.83 | 2.21E-04 |
| 4605 | Couple | 5.42 | 0.82 | 5.34 | 1.22 | 0.37 | 7.10E-01 |
| **4606** | **Romance** | **4.46** | **1.71** | **6.74** | **1.84** | **-6.52** | **2.83E-09^*^** |
| 4607 | EroticCouple | 5.06 | 1.52 | 6.25 | 1.94 | -3.46 | 7.80E-04 |
| **4608** | **EroticCouple** | **4.98** | **1.65** | **6.66** | **1.85** | **-4.88** | **4.04E-06^*^** |
| 4609 | Couple | 5.63 | 1.88 | 6.95 | 1.64 | -3.81 | 2.41E-04 |
| **4610** | **Romance** | **6.04** | **1.2** | **7.79** | **1.49** | **-6.58** | **2.10E-09^*^** |
| **4611** | **EroticCouple** | **3.77** | **1.68** | **6** | **2.06** | **-6.04** | **2.56E-08^*^** |
| 4613 | Condom | 4.42 | 1.65 | 5.28 | 1.76 | -2.57 | 1.15E-02 |
| 4614 | Romance | 6.7 | 1.06 | 7.71 | 1.4 | -4.14 | 7.14E-05 |
| 4617 | EroticFemale | 5.64 | 1.09 | 6.7 | 1.61 | -3.94 | 1.52E-04 |
| 4621 | Harassment | 3.58 | 1.27 | 2.71 | 1.38 | 3.35 | 1.15E-03 |
| **4622** | **Romance** | **5.68** | **1.34** | **8.17** | **1.23** | **-9.82** | **2.19E-16^*^** |
| **4623** | **Romance** | **5.66** | **1.2** | **7.49** | **1.88** | **-5.92** | **4.40E-08^*^** |
| **4624** | **Couple** | **5.64** | **1.29** | **7.17** | **1.65** | **-5.26** | **8.13E-07^*^** |
| 4625 | Couple | 5.83 | 1.22 | 6.59 | 1.9 | -2.43 | 1.69E-02 |
| 4626 | Wedding | 6.89 | 1.13 | 7.8 | 1.76 | -3.13 | 2.28E-03 |
| 4631 | BikerCouple | 4.88 | 1.79 | 4.8 | 1.63 | 0.22 | 8.25E-01 |
| 4635 | Prostitute | 2.8 | 1.41 | 3.2 | 1.76 | -1.28 | 2.05E-01 |
| **4640** | **Romance** | **5.26** | **1.58** | **7.64** | **1.85** | **-7.05** | **2.28E-10^*^** |
| 4641 | Romance | 6.21 | 1 | 7.21 | 1.7 | -3.65 | 4.11E-04 |
| **4650** | **EroticCouple** | **4.68** | **1.52** | **6.94** | **1.58** | **-7.4** | **4.18E-11^*^** |
| 4651 | EroticCouple | 3.65 | 2.02 | 5.15 | 1.98 | -3.81 | 2.36E-04 |
| 4652 | EroticCouple | 3.9 | 2.16 | 5.65 | 2.11 | -4.17 | 6.40E-05 |
| 4653 | EroticCouple | 5.28 | 1.54 | 6.04 | 1.81 | -2.31 | 2.30E-02 |
| 4656 | EroticCouple | 4.4 | 2.92 | 6.44 | 1.95 | -4.14 | 7.32E-05 |
| **4658** | **EroticCouple** | **3.51** | **2** | **6.08** | **2.05** | **-6.44** | **4.04E-09^*^** |
| **4659** | **EroticCouple** | **3.7** | **1.82** | **6.15** | **2.01** | **-6.49** | **3.22E-09^*^** |
| **4660** | **EroticCouple** | **4.26** | **1.61** | **7.22** | **1.4** | **-9.93** | **1.27E-16^*^** |
| **4664** | **EroticCouple** | **3.6** | **2.02** | **5.42** | **2.21** | **-4.36** | **3.16E-05^*^** |
| 4664.1 | Erotic | 3.58 | 1.99 | 4.42 | 2.26 | -2 | 4.84E-02 |
| 4664.2 | Attack | 2.26 | 1.17 | 2.21 | 1.46 | 0.17 | 8.62E-01 |
| **4666** | **EroticCouple** | **3.68** | **1.85** | **5.88** | **1.89** | **-5.97** | **3.50E-08^*^** |
| **4669** | **EroticCouple** | **3.36** | **1.66** | **5.18** | **2** | **-5.03** | **2.12E-06^*^** |
| **4670** | **EroticCouple** | **3.4** | **1.82** | **6.4** | **1.91** | **-8.18** | **8.76E-13^*^** |
| **4672** | **EroticCouple** | **3.52** | **2.09** | **5.6** | **1.85** | **-5.33** | **5.96E-07^*^** |
| **4676** | **EroticCouple** | **4.02** | **1.73** | **6.62** | **1.77** | **-7.54** | **2.05E-11^*^** |
| **4677** | **EroticCouple** | **4.51** | **1.6** | **6.63** | **1.72** | **-6.48** | **3.38E-09^*^** |
| **4680** | **EroticCouple** | **3.59** | **1.95** | **6.91** | **1.92** | **-8.71** | **6.21E-14^*^** |
| **4681** | **EroticCouple** | **4.55** | **1.8** | **6.42** | **2.07** | **-4.89** | **3.85E-06^*^** |
| 4683 | EroticCouple | 3.91 | 1.7 | 5.29 | 2.03 | -3.73 | 3.12E-04 |
| **4687** | **EroticCouple** | **4.39** | **1.9** | **6.64** | **1.59** | **-6.5** | **3.12E-09^*^** |
| 4689 | EroticCouple | 5.45 | 1.69 | 6.82 | 1.68 | -4.13 | 7.43E-05 |
| **4690** | **EroticCouple** | **3.26** | **1.79** | **6.43** | **1.84** | **-8.88** | **2.58E-14^*^** |
| 4700 | Couple | 6.06 | 0.82 | 7.08 | 1.87 | -3.61 | 4.82E-04 |
| 4750 | NudeFemale | 4.81 | 1.93 | 4.56 | 1.63 | 0.71 | 4.81E-01 |
| 4770 | FemaleKiss | 4.27 | 1.91 | 3.47 | 2.19 | 1.98 | 5.06E-02 |
| **4800** | **EroticCouple** | **3.21** | **1.65** | **5.45** | **2.28** | **-5.73** | **1.06E-07^*^** |
| **4810** | **EroticCouple** | **3.28** | **2.31** | **5.98** | **2.11** | **-6.18** | **1.35E-08^*^** |
| **5000** | **Flower** | **6.15** | **1.14** | **7.59** | **1.63** | **-5.23** | **9.38E-07^*^** |
| **5001** | **SunFlower** | **6.45** | **1.28** | **7.78** | **1.33** | **-5.18** | **1.14E-06^*^** |
| **5010** | **Flower** | **6.26** | **1.13** | **7.55** | **1.39** | **-5.2** | **1.05E-06^*^** |
| 5020 | Flower | 6.09 | 1.08 | 6.64 | 1.69 | -1.98 | 5.05E-02 |
| **5030** | **Flower** | **5.89** | **1.36** | **7.18** | **1.56** | **-4.48** | **1.97E-05^*^** |
| 5120 | PineNeedles | 3.06 | 1.33 | 4.15 | 1.53 | -3.86 | 2.02E-04 |
| **5130** | **Rocks** | **3.4** | **1.35** | **4.52** | **1.1** | **-4.59** | **1.27E-05^*^** |
| **5200** | **Flowers** | **6.53** | **1.14** | **7.69** | **1.37** | **-4.68** | **9.10E-06^*^** |
| 5201 | Nature | 6.72 | 1.05 | 7.59 | 1.5 | -3.44 | 8.45E-04 |
| 5220 | Nature | 6.6 | 1.21 | 7.06 | 1.62 | -1.65 | 1.01E-01 |
| 5250 | Nature | 5.35 | 1.46 | 6.11 | 1.99 | -2.22 | 2.87E-02 |
| 5260 | Waterfall | 5.96 | 1.37 | 7.2 | 1.79 | -3.96 | 1.39E-04 |
| **5270** | **Nature** | **5.72** | **1.44** | **7.32** | **1.66** | **-5.22** | **9.49E-07^*^** |
| 5300 | Galaxy | 6.74 | 1.17 | 6.96 | 1.86 | -0.71 | 4.81E-01 |
| 5390 | Boat | 6.15 | 1.11 | 6.02 | 1.4 | 0.53 | 5.96E-01 |
| **5395** | **Boat** | **4.11** | **1.48** | **5.33** | **1.21** | **-4.58** | **1.32E-05^*^** |
| 5410 | Violinist | 6.28 | 1.58 | 6.41 | 1.63 | -0.42 | 6.74E-01 |
| 5450 | Liftoff | 6.68 | 1.12 | 7 | 1.46 | -1.25 | 2.13E-01 |
| 5455 | Cockpit | 4.87 | 0.85 | 5.72 | 1.52 | -3.52 | 6.51E-04 |
| **5460** | **Astronaut** | **5.3** | **1.23** | **7.3** | **1.58** | **-7.2** | **1.10E-10^*^** |
| **5470** | **Astronaut** | **6.19** | **1.19** | **7.33** | **1.44** | **-4.38** | **2.86E-05^*^** |
| 5480 | Fireworks | 6.79 | 1.18 | 7.69 | 1.45 | -3.46 | 7.83E-04 |
| 5500 | Mushroom | 4.91 | 0.69 | 5.34 | 1.49 | -1.9 | 6.07E-02 |
| 5510 | Mushroom | 5.38 | 1.33 | 5.1 | 1.35 | 1.07 | 2.87E-01 |
| 5520 | Mushroom | 4.91 | 1.04 | 5.39 | 1.21 | -2.14 | 3.46E-02 |
| 5530 | Mushroom | 4.8 | 1.39 | 5.44 | 1.51 | -2.24 | 2.73E-02 |
| **5531** | **Mushroom** | **3.74** | **1.5** | **5.07** | **1.38** | **-4.67** | **9.46E-06^*^** |
| 5532 | Mushrooms | 3.94 | 1.21 | 4.99 | 1.66 | -3.7 | 3.48E-04 |
| 5533 | Mushrooms | 5.26 | 1.11 | 5.49 | 1.01 | -1.12 | 2.66E-01 |
| 5534 | Mushrooms | 4.06 | 1.49 | 4.96 | 1.27 | -3.29 | 1.38E-03 |
| 5535 | Stilllife | 4.72 | 1.46 | 4.72 | 1.74 | 0.01 | 9.91E-01 |
| **5551** | **Clouds** | **6.02** | **1.38** | **7.79** | **1.62** | **-5.99** | **3.25E-08^*^** |
| **5593** | **Sky** | **5.26** | **1.21** | **6.69** | **1.47** | **-5.43** | **3.90E-07^*^** |
| **5594** | **Sky** | **5.44** | **1.88** | **7.54** | **1.5** | **-6.26** | **9.61E-09^*^** |
| 5600 | Moutains | 7.1 | 1.63 | 7.83 | 1.28 | -2.51 | 1.38E-02 |
| **5611** | **Moutains** | **5.19** | **1.33** | **7.33** | **1.7** | **-7.14** | **1.49E-10^*^** |
| **5621** | **SkyDivers** | **6.6** | **1.08** | **7.8** | **1.54** | **-4.62** | **1.12E-05^*^** |
| 5622 | Shark | 5.98 | 1.51 | 6.23 | 2.08 | -0.71 | 4.81E-01 |
| 5623 | Windsurfers | 6.51 | 1.18 | 7.26 | 2.08 | -2.27 | 2.54E-02 |
| 5626 | HangGlider | 6.83 | 1.4 | 6.62 | 2.34 | 0.56 | 5.79E-01 |
| 5628 | Moutains | 5.85 | 1.06 | 6.6 | 2.9 | -1.76 | 8.15E-02 |
| **5629** | **Hiker** | **5.74** | **1.24** | **7.15** | **1.51** | **-5.17** | **1.18E-06^*^** |
| **5660** | **Moutains** | **6** | **1.41** | **7.38** | **1.53** | **-4.76** | **6.55E-06^*^** |
| 5661 | Cave | 4.89 | 1.03 | 5.91 | 1.42 | -4.18 | 6.14E-05 |
| **5700** | **Moutains** | **5.21** | **1.32** | **7.54** | **1.56** | **-8.2** | **7.95E-13^*^** |
| 5711 | Field | 6.13 | 1.15 | 6.92 | 1.86 | -2.62 | 1.02E-02 |
| 5720 | Farmland | 5.49 | 1.37 | 6.58 | 1.51 | -3.85 | 2.07E-04 |
| 5731 | Flowers | 5.89 | 1.31 | 5.58 | 1.52 | 1.13 | 2.63E-01 |
| 5740 | Plant | 5.15 | 1.06 | 5.33 | 1.47 | -0.72 | 4.76E-01 |
| **5750** | **Nature** | **3.15** | **1.16** | **6.87** | **1.91** | **-12.02** | **3.35E-21^*^** |
| **5760** | **Nature** | **6.63** | **1** | **8.41** | **1.07** | **-8.74** | **5.19E-14^*^** |
| **5779** | **Courtyard** | **6.28** | **1.35** | **7.72** | **1.41** | **-5.31** | **6.42E-07^*^** |
| 5780 | Nature | 6.79 | 1.4 | 7.68 | 1.44 | -3.19 | 1.87E-03 |
| 5800 | Leaves | 6.53 | 1.23 | 6.51 | 1.57 | 0.07 | 9.43E-01 |
| **5811** | **Flowers** | **6.4** | **1.23** | **7.88** | **1.24** | **-6.07** | **2.25E-08^*^** |
| **5820** | **Moutains** | **6.23** | **1.51** | **7.65** | **1.51** | **-4.76** | **6.40E-06^*^** |
| **5830** | **Sunset** | **7.5** | **1.17** | **8.54** | **0.82** | **-5.21** | **1.01E-06^*^** |
| **5831** | **Seagulls** | **6.47** | **1.5** | **8.05** | **1** | **-6.26** | **9.60E-09^*^** |
| 5849 | Flowers | 6.57 | 1.12 | 6.82 | 2.22 | -0.73 | 4.68E-01 |
| 5870 | Clouds | 5.96 | 1 | 6.92 | 1.86 | -3.3 | 1.35E-03 |
| 5875 | Bicyclist | 5 | 1.22 | 6.16 | 1.61 | -4.14 | 7.32E-05 |
| **5890** | **Earth** | **5.26** | **1.48** | **6.73** | **1.58** | **-4.89** | **3.83E-06^*^** |
| **5891** | **Clouds** | **5.77** | **1.16** | **7.58** | **1.54** | **-6.77** | **8.71E-10^*^** |
| 5900 | Desert | 5.49 | 0.91 | 5.75 | 1.58 | -1.03 | 3.05E-01 |
| **5910** | **Fireworks** | **5.93** | **1.42** | **8.16** | **1.15** | **-8.73** | **5.64E-14^*^** |
| 5920 | Volcano | 4.55 | 1.57 | 4.63 | 1.92 | -0.22 | 8.24E-01 |
| 5940 | Lava | 3.85 | 1.6 | 3.76 | 1.41 | 0.31 | 7.60E-01 |
| 5950 | Lightning | 4.72 | 1.73 | 5.34 | 2.2 | -1.59 | 1.16E-01 |
| 5970 | Tornado | 3.02 | 1.52 | 4.03 | 1.85 | -3.03 | 3.08E-03 |
| **5971** | **Tornado** | **4.57** | **1.1** | **2.97** | **1.81** | **5.45** | **3.63E-07^*^** |
| 5972 | Tornado | 4.04 | 1.61 | 3.67 | 2.67 | 0.86 | 3.91E-01 |
| **5982** | **Sky** | **6.38** | **1.45** | **7.85** | **1.26** | **-5.49** | **3.00E-07^*^** |
| 5990 | Sky | 5.98 | 2.07 | 6.54 | 1.8 | -1.46 | 1.47E-01 |
| 5991 | Sky | 6.38 | 1.17 | 7.06 | 1.75 | -2.33 | 2.18E-02 |
| **5994** | **Skyline** | **5.61** | **1.32** | **7.1** | **1.92** | **-4.61** | **1.17E-05^*^** |
| 6000 | Prison | 4.64 | 1.28 | 3.92 | 1.82 | 2.33 | 2.18E-02 |
| 6010 | Jail | 3.31 | 1.14 | 3.37 | 1.61 | -0.22 | 8.27E-01 |
| 6020 | ElectricChair | 3.19 | 1.14 | 2.93 | 1.71 | 0.92 | 3.60E-01 |
| 6150 | Outlet | 4.91 | 1.04 | 5 | 1.21 | -0.38 | 7.02E-01 |
| 6190 | AimedGun | 3.7 | 1.56 | 2.91 | 1.59 | 2.55 | 1.22E-02 |
| 6200 | AimedGun | 3.57 | 1.21 | 2.71 | 1.43 | 3.32 | 1.26E-03 |
| **6210** | **AimedGun** | **4.02** | **1.22** | **2.15** | **1.42** | **7.18** | **1.23E-10^*^** |
| 6211 | Attack | 3.13 | 1.44 | 3 | 2.28 | 0.34 | 7.34E-01 |
| **6212** | **Soldier** | **3.57** | **1.47** | **1.81** | **1.41** | **6.21** | **1.19E-08^*^** |
| **6213** | **Terrorist** | **3.51** | **0.93** | **2.41** | **1.38** | **4.77** | **6.18E-06^*^** |
| 6230 | AimedGun | 2.46 | 1.24 | 2.06 | 1.59 | 1.42 | 1.58E-01 |
| **6241** | **Gun** | **4.26** | **0.87** | **2.92** | **1.49** | **5.59** | **1.94E-07^*^** |
| **6243** | **AimedGun** | **3.08** | **1.35** | **1.9** | **1.23** | **4.64** | **1.04E-05^*^** |
| 6244 | AimedGun | 3.69 | 1.6 | 2.53 | 1.44 | 3.85 | 2.07E-04 |
| 6250 | AimedGun | 3.38 | 1.31 | 2.7 | 1.62 | 2.36 | 2.03E-02 |
| 6250.2 | IceCream | 6.26 | 1.39 | 6.5 | 1.84 | -0.76 | 4.46E-01 |
| 6260 | AimedGun | 2.75 | 1.44 | 2.35 | 1.45 | 1.41 | 1.63E-01 |
| 6300 | Knife | 2.08 | 1.05 | 1.94 | 1.36 | 0.59 | 5.59E-01 |
| 6311 | DistressedFem | 3.26 | 1.28 | 2.36 | 1.72 | 3.01 | 3.28E-03 |
| 6312 | Abduction | 3.11 | 1.46 | 2.08 | 1.47 | 3.55 | 5.89E-04 |
| 6313 | Attack | 2.57 | 1.49 | 1.61 | 1.22 | 3.59 | 5.16E-04 |
| 6314 | Attack | 4.94 | 1.63 | 4.25 | 1.61 | 2.15 | 3.38E-02 |
| **6315** | **BeatenFem** | **2.81** | **1.15** | **1.72** | **1.23** | **4.63** | **1.08E-05^*^** |
| **6350** | **Attack** | **2.46** | **1.17** | **1.44** | **0.95** | **4.84** | **4.66E-06^*^** |
| 6360 | Attack | 2.38 | 1.41 | 1.86 | 1.69 | 1.68 | 9.53E-02 |
| 6370 | Attack | 2.11 | 1.03 | 2.2 | 1.31 | -0.39 | 6.98E-01 |
| **6410** | **AimedGun** | **4.3** | **1.18** | **2.54** | **1.88** | **5.72** | **1.08E-07^*^** |
| 6415 | DeadTiger | 2.56 | 1.32 | 1.65 | 1.15 | 3.73 | 3.15E-04 |
| 6510 | Attack | 2.27 | 1.53 | 2.06 | 1.28 | 0.75 | 4.52E-01 |
| **6530** | **Attack** | **4.53** | **1.82** | **2.65** | **1.79** | **5.29** | **7.11E-07^*^** |
| **6540** | **Attack** | **3.17** | **1.79** | **1.86** | **1.14** | **4.39** | **2.83E-05^*^** |
| 6550 | Attack | 1.94 | 1.24 | 2.08 | 1.9 | -0.45 | 6.51E-01 |
| 6555 | Knife | 3.06 | 1.17 | 2.95 | 1.65 | 0.39 | 6.96E-01 |
| 6560 | Attack | 2.6 | 1.06 | 1.78 | 1.23 | 3.61 | 4.71E-04 |
| **6561** | **Attack** | **4.38** | **1.45** | **2.79** | **1.44** | **5.56** | **2.24E-07^*^** |
| 6570 | Suicide | 2.54 | 1.52 | 2.1 | 1.61 | 1.43 | 1.55E-01 |
| 6570.1 | Suicide | 2.48 | 1.24 | 2.25 | 1.88 | 0.73 | 4.64E-01 |
| 6570.2 | BlowDry | 4.11 | 1.48 | 4.84 | 0.93 | -3 | 3.44E-03 |
| 6571 | CarTheft | 3.15 | 1.47 | 2.15 | 1.65 | 3.24 | 1.60E-03 |
| **6610** | **Gun** | **3.79** | **1.2** | **2.58** | **1.64** | **4.29** | **4.18E-05^*^** |
| **6800** | **Gun** | **4.11** | **1.07** | **2.87** | **1.53** | **4.78** | **6.02E-06^*^** |
| **6821** | **Gang** | **3.53** | **1.59** | **1.85** | **1.31** | **5.84** | **6.26E-08^*^** |
| 6830 | Guns | 3.17 | 1.32 | 2.31 | 1.7 | 2.87 | 4.94E-03 |
| 6831 | Police | 2.68 | 1.25 | 2.21 | 1.52 | 1.72 | 8.91E-02 |
| **6834** | **Police** | **3.78** | **1.09** | **2.4** | **1.51** | **5.35** | **5.62E-07^*^** |
| 6836 | Police | 3.45 | 1.21 | 3.05 | 1.46 | 1.5 | 1.36E-01 |
| **6838** | **Police** | **4.51** | **1.44** | **2.2** | **1.34** | **8.41** | **2.77E-13^*^** |
| 6840 | Police | 3.57 | 1.44 | 3.07 | 1.86 | 1.54 | 1.26E-01 |
| 6900 | Aircraft | 5.17 | 1.2 | 4.44 | 2.1 | 2.18 | 3.15E-02 |
| 6910 | Bomber | 5.3 | 1.52 | 4.49 | 2.05 | 2.3 | 2.36E-02 |
| 6930 | Missiles | 5.34 | 0.92 | 4.33 | 1.84 | 3.56 | 5.69E-04 |
| 6940 | Tank | 3.91 | 1.4 | 2.69 | 1.73 | 3.94 | 1.48E-04 |
| 7000 | RollingPin | 4.83 | 0.99 | 5.06 | 1.1 | -1.12 | 2.64E-01 |
| 7002 | Towel | 4.79 | 0.91 | 5.03 | 0.98 | -1.31 | 1.95E-01 |
| 7004 | Spoon | 4.89 | 1.05 | 5.14 | 0.59 | -1.46 | 1.48E-01 |
| 7006 | Bowl | 5 | 1.23 | 5.09 | 0.81 | -0.44 | 6.64E-01 |
| 7009 | Mug | 5.09 | 0.62 | 4.89 | 0.96 | 1.23 | 2.21E-01 |
| 7010 | Basket | 4.98 | 0.85 | 4.92 | 0.48 | 0.42 | 6.72E-01 |
| 7020 | Fan | 4.57 | 0.9 | 4.94 | 0.88 | -2.11 | 3.75E-02 |
| 7025 | Stool | 4.9 | 1.02 | 4.79 | 1.1 | 0.5 | 6.15E-01 |
| 7030 | Iron | 4.3 | 1.1 | 4.57 | 1.08 | -1.27 | 2.07E-01 |
| 7031 | Shoes | 3.88 | 1.52 | 4.8 | 0.81 | -3.81 | 2.36E-04 |
| 7034 | Hammer | 4.17 | 1.29 | 4.91 | 0.56 | -3.74 | 3.09E-04 |
| 7035 | Mug | 5.15 | 0.88 | 5.15 | 0.84 | 0 | 1.00E+00 |
| 7036 | Shipyard | 5.14 | 1.04 | 4.71 | 1.1 | 2.04 | 4.42E-02 |
| 7037 | Trains | 5.06 | 1.13 | 4.75 | 1.14 | 1.4 | 1.64E-01 |
| 7038 | Shoes | 4.32 | 0.96 | 4.68 | 1.4 | -1.53 | 1.29E-01 |
| **7039** | **Train** | **5.09** | **0.97** | **6.29** | **1.74** | **-4.36** | **3.13E-05^*^** |
| 7040 | DustPan | 4.43 | 0.99 | 4.66 | 1 | -1.17 | 2.45E-01 |
| 7041 | Baskets | 4.43 | 1.14 | 5.02 | 1.11 | -2.69 | 8.48E-03 |
| 7050 | HairDryer | 5.11 | 1.01 | 5.04 | 0.87 | 0.38 | 7.08E-01 |
| 7060 | TrashCan | 3.88 | 1.78 | 4.29 | 1.35 | -1.31 | 1.93E-01 |
| 7080 | Fork | 4.7 | 0.83 | 5.1 | 0.88 | -2.36 | 2.02E-02 |
| 7090 | Book | 4.81 | 1.08 | 5.44 | 1.35 | -2.63 | 9.74E-03 |
| **7095** | **Headlight** | **4.68** | **1.04** | **6.13** | **1.44** | **-5.87** | **5.54E-08^*^** |
| 7096 | Car | 4.74 | 1.01 | 5.44 | 1.29 | -3.05 | 2.88E-03 |
| 7100 | FireHydrant | 4.87 | 1.06 | 5.2 | 1.39 | -1.36 | 1.77E-01 |
| 7110 | Hammer | 3.77 | 1.35 | 4.59 | 0.84 | -3.68 | 3.82E-04 |
| 7130 | Truck | 4.53 | 0.83 | 4.75 | 0.94 | -1.26 | 2.12E-01 |
| 7140 | Bus | 5.11 | 0.56 | 5.41 | 1.5 | -1.38 | 1.72E-01 |
| 7150 | Umbrella | 5 | 0.92 | 4.69 | 1.19 | 1.48 | 1.41E-01 |
| **7160** | **Fabric** | **4** | **1.27** | **5.05** | **1.19** | **-4.33** | **3.56E-05^*^** |
| 7161 | Pole | 4.85 | 0.93 | 4.97 | 1.16 | -0.58 | 5.67E-01 |
| 7170 | LightBulb | 4.94 | 1.55 | 5.33 | 1.49 | -1.31 | 1.92E-01 |
| 7175 | Lamp | 5.23 | 1.04 | 4.95 | 0.8 | 1.52 | 1.31E-01 |
| 7179 | Rug | 5.67 | 1.1 | 5.01 | 1.13 | 3.03 | 3.14E-03 |
| 7180 | NeonBuilding | 5.46 | 1.75 | 4.7 | 1.22 | 2.54 | 1.27E-02 |
| 7182 | Checkerboard | 4.83 | 1.51 | 5.03 | 1.38 | -0.7 | 4.85E-01 |
| 7183 | Checkerboard | 4.64 | 1.29 | 5.53 | 1.23 | -3.58 | 5.26E-04 |
| **7184** | **AbstractArt** | **3.4** | **1.25** | **4.78** | **1.07** | **-6** | **3.11E-08^*^** |
| 7185 | AbstractArt | 4.91 | 0.88 | 5.08 | 0.64 | -1.08 | 2.81E-01 |
| 7186 | AbstractArt | 5.51 | 1.08 | 4.38 | 1.71 | 4.03 | 1.07E-04 |
| 7187 | AbstractArt | 5.19 | 1.1 | 5.25 | 0.89 | -0.3 | 7.62E-01 |
| 7190 | Clock | 5.34 | 1.24 | 5.59 | 1.27 | -1.01 | 3.15E-01 |
| **7195** | **Teeth** | **4.02** | **1.62** | **5.88** | **1.79** | **-5.53** | **2.50E-07^*^** |
| **7200** | **Brownie** | **6.3** | **1.27** | **7.77** | **1.71** | **-4.98** | **2.61E-06^*^** |
| 7205 | Scarves | 5.3 | 0.98 | 5.75 | 1.41 | -1.89 | 6.17E-02 |
| 7207 | Beads | 5.13 | 1.42 | 5.3 | 1.44 | -0.61 | 5.42E-01 |
| 7211 | Clock | 4.89 | 0.56 | 4.69 | 1.92 | 0.74 | 4.63E-01 |
| 7217 | clothesRack | 4.6 | 1.23 | 5 | 0.78 | -1.95 | 5.34E-02 |
| 7220 | Pastry | 7 | 1.08 | 7.19 | 1.68 | -0.69 | 4.94E-01 |
| 7224 | FileCabinets | 4.75 | 1.25 | 4.51 | 1.25 | 0.98 | 3.32E-01 |
| 7230 | Turkey | 6.55 | 1.27 | 7.35 | 1.8 | -2.62 | 1.01E-02 |
| 7233 | Plate | 5.49 | 1.28 | 5.15 | 1.66 | 1.16 | 2.47E-01 |
| 7234 | IroningBoard | 4.7 | 0.86 | 4.12 | 1.73 | 2.18 | 3.14E-02 |
| 7235 | Chair | 5.17 | 1.09 | 5.06 | 1.22 | 0.48 | 6.30E-01 |
| 7236 | Lightbulb | 5.24 | 1.23 | 5.55 | 1.28 | -1.25 | 2.13E-01 |
| 7237 | AbstractArt | 4.7 | 1.31 | 5.45 | 1.41 | -2.81 | 5.95E-03 |
| 7238 | AbstractArt | 6.38 | 1.39 | 6.55 | 1.53 | -0.58 | 5.63E-01 |
| 7250 | Cake | 6.4 | 1.17 | 6.6 | 1.71 | -0.68 | 4.97E-01 |
| 7260 | Torte | 6.53 | 1.8 | 7.31 | 1.83 | -2.18 | 3.16E-02 |
| **7270** | **IceCream** | **5.61** | **2.07** | **7.77** | **1.68** | **-5.79** | **7.84E-08^*^** |
| **7280** | **Wines** | **5.87** | **0.99** | **7.73** | **1.48** | **-7.52** | **2.27E-11^*^** |
| 7281 | Food | 6.62 | 1.44 | 6.66 | 1.72 | -0.13 | 8.98E-01 |
| 7282 | Cake | 7.13 | 1.38 | 7.13 | 1.41 | -0.01 | 9.91E-01 |
| 7283 | Fruit | 6.55 | 1.56 | 5.88 | 2 | 1.91 | 5.88E-02 |
| 7284 | Fruit | 6.15 | 1.76 | 6.47 | 1.65 | -0.96 | 3.37E-01 |
| 7285 | Tomatoes | 6.63 | 1.51 | 5.99 | 1.81 | 1.94 | 5.55E-02 |
| 7286 | Pancakes | 6.87 | 1.41 | 6.41 | 1.68 | 1.51 | 1.35E-01 |
| 7289 | Food | 6.55 | 1.18 | 6.16 | 2.01 | 1.22 | 2.26E-01 |
| 7291 | Chicken | 6.34 | 1.67 | 6.36 | 2.1 | -0.05 | 9.57E-01 |
| 7320 | Desserts | 5.77 | 1.46 | 6.69 | 1.7 | -2.95 | 3.93E-03 |
| 7325 | Watermelon | 7.11 | 1.32 | 7.48 | 1.66 | -1.27 | 2.08E-01 |
| 7330 | IceCream | 7.09 | 1.76 | 7.96 | 1.49 | -2.71 | 7.83E-03 |
| 7340 | IceCream | 5.89 | 1.81 | 6.87 | 1.63 | -2.87 | 4.93E-03 |
| 7350 | Pizza | 6.94 | 1.24 | 7.11 | 1.89 | -0.54 | 5.89E-01 |
| 7351 | Pizza | 6.23 | 1.48 | 6.06 | 1.6 | 0.57 | 5.67E-01 |
| 7352 | Pizza | 6.04 | 1.71 | 6.12 | 2.21 | -0.2 | 8.41E-01 |
| **7359** | **PieW/bug** | **5.49** | **2.39** | **2.52** | **1.56** | **7.43** | **3.59E-11^*^** |
| **7360** | **FliesOnPie** | **6.68** | **1.49** | **3.11** | **1.79** | **11.03** | **5.00E-19^*^** |
| 7361 | MeatSlicer | 2.02 | 1.82 | 2.55 | 1.73 | -1.51 | 1.35E-01 |
| 7380 | RoachOnPizza | 2.94 | 1.49 | 2.31 | 1.26 | 2.29 | 2.39E-02 |
| 7390 | IceCream | 5.81 | 1.28 | 6.98 | 1.89 | -3.7 | 3.55E-04 |
| **7400** | **Candy** | **5.83** | **1.61** | **7.3** | **1.73** | **-4.48** | **2.00E-05^*^** |
| 7402 | Pastry | 6.89 | 1.27 | 5.84 | 2.33 | 2.87 | 4.98E-03 |
| 7410 | Candy | 6.45 | 0.97 | 7 | 1.63 | -2.11 | 3.75E-02 |
| 7430 | Candy | 6.68 | 1.38 | 7.35 | 1.7 | -2.2 | 3.00E-02 |
| 7450 | Cheeseburger | 6.74 | 1.37 | 6.28 | 2.15 | 1.31 | 1.92E-01 |
| 7460 | FrenchFries | 6.96 | 1.61 | 6.71 | 2.26 | 0.65 | 5.18E-01 |
| 7470 | Pancakes | 6.28 | 1.71 | 7.18 | 1.7 | -2.68 | 8.67E-03 |
| 7472 | Grapes | 6.77 | 1.15 | 6.14 | 2.13 | 1.87 | 6.41E-02 |
| 7475 | Shrimp | 6.47 | 1.73 | 6.59 | 1.62 | -0.36 | 7.17E-01 |
| 7480 | Pasta | 6.34 | 1.18 | 7 | 1.62 | -2.37 | 1.95E-02 |
| 7481 | Food | 6.83 | 1.24 | 6.58 | 2.06 | 0.75 | 4.55E-01 |
| **7490** | **Window** | **4.6** | **0.9** | **5.66** | **1.48** | **-4.44** | **2.32E-05^*^** |
| 7491 | Building | 4.81 | 1.21 | 4.79 | 1.09 | 0.09 | 9.30E-01 |
| 7493 | Man | 4.98 | 1.32 | 5.56 | 1.5 | -2.09 | 3.95E-02 |
| 7495 | Store | 5.26 | 1.06 | 5.96 | 1.67 | -2.57 | 1.16E-02 |
| **7496** | **Street** | **4.81** | **1.12** | **5.99** | **1.7** | **-4.19** | **5.90E-05^*^** |
| 7500 | Building | 5.3 | 0.95 | 5.23 | 1.5 | 0.28 | 7.83E-01 |
| **7501** | **City** | **5.46** | **1.19** | **6.8** | **1.79** | **-4.51** | **1.72E-05^*^** |
| **7502** | **Castle** | **6.34** | **1.56** | **8.15** | **1.25** | **-6.47** | **3.49E-09^*^** |
| 7503 | CardDealer | 4.51 | 1.14 | 5.59 | 1.13 | -4.83 | 4.96E-06^*^ |
| 7504 | Stairs | 4.89 | 0.92 | 5.65 | 1.52 | -3.09 | 2.62E-03 |
| 7510 | Skyscraper | 5.51 | 1 | 5.98 | 1.55 | -1.84 | 6.86E-02 |
| **7545** | **Ocean** | **5.4** | **0.92** | **7.04** | **1.71** | **-6.09** | **2.09E-08^*^** |
| 7550 | Office | 4.96 | 0.72 | 5.17 | 1.52 | -0.9 | 3.68E-01 |
| 7560 | Freeway | 5 | 1.2 | 4.5 | 1.62 | 1.79 | 7.67E-02 |
| **7570** | **Skyline** | **4.83** | **1.49** | **7.27** | **1.48** | **-8.33** | **4.19E-13^*^** |
| 7580 | Desert | 6.79 | 1.64 | 7.59 | 1.44 | -2.63 | 9.78E-03 |
| 7590 | Traffic | 5.21 | 1.37 | 5.18 | 1.53 | 0.11 | 9.11E-01 |
| 7595 | Traffic | 4.68 | 0.78 | 4.53 | 1.32 | 0.71 | 4.79E-01 |
| 7600 | Dragon | 4.62 | 1.81 | 5.71 | 2.02 | -2.89 | 4.75E-03 |
| 7620 | Jet | 5.09 | 1.12 | 5.73 | 1.77 | -2.21 | 2.97E-02 |
| 7640 | Skyscraper | 5 | 0.88 | 4.69 | 1.37 | 1.37 | 1.72E-01 |
| 7700 | Office | 4.25 | 1.44 | 4.2 | 1.39 | 0.18 | 8.58E-01 |
| 7705 | Cabinet | 5.06 | 1.24 | 4.78 | 1.15 | 1.2 | 2.33E-01 |
| 7710 | Bed | 4.36 | 1.17 | 5.49 | 1.62 | -4.08 | 9.08E-05 |
| 7820 | Agate | 5.45 | 1.25 | 5.18 | 1.34 | 1.06 | 2.93E-01 |
| 7830 | Agate | 5.11 | 1.4 | 5.22 | 1.24 | -0.42 | 6.75E-01 |
| 7900 | Violin | 5.4 | 1.08 | 6.5 | 1.69 | -3.96 | 1.40E-04 |
| **7920** | **CarCrash** | **3.26** | **1.11** | **4.36** | **1.26** | **-4.72** | **7.48E-06^*^** |
| 7950 | Tissue | 5.34 | 1.13 | 5.17 | 1.12 | 0.77 | 4.44E-01 |
| 8010 | Runner | 4.96 | 0.93 | 4.24 | 1.85 | 2.52 | 1.35E-02 |
| 8021 | Skier | 5.91 | 1.12 | 6.88 | 1.32 | -4.01 | 1.17E-04 |
| **8030** | **Skier** | **5.81** | **0.85** | **7.35** | **1.86** | **-5.46** | **3.40E-07^*^** |
| 8031 | Skier | 6.19 | 1.08 | 6.75 | 1.57 | -2.12 | 3.66E-02 |
| 8032 | IceSkater | 6.68 | 1.14 | 7.25 | 1.31 | -2.36 | 2.04E-02 |
| **8033** | **IceSkater** | **5.4** | **1.57** | **7.36** | **1.35** | **-6.78** | **8.33E-10^*^** |
| **8034** | **Skier** | **5.62** | **1.41** | **7.19** | **1.63** | **-5.24** | **8.94E-07^*^** |
| 8040 | Diver | 5.87 | 1.17 | 6.7 | 1.59 | -3.02 | 3.21E-03 |
| **8041** | **Diver** | **6.19** | **1.01** | **7.48** | **1.28** | **-5.7** | **1.22E-07^*^** |
| 8050 | Rower | 5.21 | 1.52 | 6.36 | 1.46 | -3.93 | 1.58E-04 |
| 8060 | Boxer | 3.51 | 1.57 | 4.5 | 2.03 | -2.78 | 6.55E-03 |
| **8080** | **Sailing** | **5.28** | **1.31** | **7.73** | **1.43** | **-9.08** | **9.69E-15^*^** |
| **8090** | **Gymnast** | **6.06** | **0.92** | **7.42** | **1.2** | **-6.48** | **3.44E-09^*^** |
| **8116** | **Football** | **4.66** | **1.32** | **6.7** | **1.74** | **-6.72** | **1.07E-09^*^** |
| 8117 | Hockey | 4.91 | 1.08 | 5.67 | 1.84 | -2.56 | 1.19E-02 |
| 8120 | Athlete | 6.7 | 1.23 | 7.15 | 1.4 | -1.74 | 8.57E-02 |
| 8130 | PoleVaulter | 6 | 1.03 | 6.83 | 1.42 | -3.41 | 9.43E-04 |
| 8160 | RockClimber | 5.36 | 1.09 | 4.69 | 1.92 | 2.19 | 3.06E-02 |
| **8161** | **HangGlider** | **5.91** | **0.8** | **7.39** | **1.62** | **-5.93** | **4.26E-08^*^** |
| 8162 | HotAirBalloon | 6.77 | 1.2 | 7.39 | 1.62 | -2.23 | 2.80E-02 |
| **8170** | **Sailboat** | **6.09** | **1.18** | **7.59** | **1.24** | **-6.3** | **7.80E-09^*^** |
| 8178 | Cliffdiver | 5.79 | 1.53 | 6.14 | 2.29 | -0.92 | 3.57E-01 |
| 8179 | Bungee | 5.72 | 0.98 | 6.04 | 2.56 | -0.85 | 3.99E-01 |
| 8180 | CliffDivers | 5.81 | 1.26 | 6.86 | 1.94 | -3.28 | 1.44E-03 |
| **8185** | **SkyDivers** | **5.82** | **1.33** | **7.75** | **1.46** | **-7.01** | **2.72E-10^*^** |
| 8186 | Skysurfer | 5.77 | 1.22 | 6.83 | 1.7 | -3.67 | 3.95E-04 |
| **8190** | **Skier** | **6.83** | **1.45** | **8.08** | **1.48** | **-4.33** | **3.47E-05^*^** |
| 8191 | IceClimber | 6.17 | 1.17 | 5.7 | 1.76 | 1.61 | 1.11E-01 |
| 8192 | VolcanoSkier | 4.36 | 1.33 | 5.21 | 1.58 | -2.96 | 3.78E-03 |
| 8193 | Skier | 6 | 1.04 | 6.91 | 1.71 | -3.28 | 1.42E-03 |
| **8200** | **WaterSkier** | **5.96** | **1.41** | **7.86** | **1.12** | **-7.54** | **2.05E-11^*^** |
| **8210** | **Boat** | **6.26** | **0.99** | **7.6** | **1.36** | **-5.76** | **8.96E-08^*^** |
| 8211 | Sailboat | 5.3 | 0.98 | 5.54 | 2.03 | -0.78 | 4.38E-01 |
| 8220 | Runners | 6.31 | 1.32 | 6.53 | 1.82 | -0.7 | 4.88E-01 |
| 8230 | Boxer | 1.88 | 1.52 | 2.11 | 1.23 | -0.86 | 3.93E-01 |
| 8231 | Boxer | 2.81 | 1.16 | 3.19 | 1.81 | -1.28 | 2.05E-01 |
| 8232 | Boxer | 4.72 | 1.25 | 4.6 | 1.67 | 0.43 | 6.70E-01 |
| 8250 | Motorcyclist | 5.13 | 0.92 | 5.84 | 1.5 | -2.92 | 4.31E-03 |
| 8251 | Motorcycle | 5.49 | 1.38 | 5.89 | 1.79 | -1.28 | 2.04E-01 |
| 8260 | Motorcyclist | 5.55 | 1 | 5.47 | 1.72 | 0.3 | 7.63E-01 |
| 8280 | Diver | 5.45 | 1.06 | 6.33 | 1.59 | -3.34 | 1.18E-03 |
| **8300** | **Pilot** | **5.07** | **1.04** | **6.54** | **1.66** | **-5.43** | **3.82E-07^*^** |
| 8311 | Golfer | 5.72 | 1.04 | 5.67 | 1.35 | 0.22 | 8.23E-01 |
| 8320 | CarRacer | 5.57 | 0.8 | 6.14 | 1.88 | -2.01 | 4.75E-02 |
| 8330 | Winner | 6.09 | 1.21 | 7.04 | 1.48 | -3.59 | 5.09E-04 |
| **8340** | **Plane** | **5.09** | **1** | **6.29** | **1.47** | **-4.9** | **3.73E-06^*^** |
| 8341 | Wingwalker | 5.21 | 1.08 | 5.81 | 2.09 | -1.84 | 6.93E-02 |
| **8350** | **TennisPlayer** | **5.89** | **1.26** | **7.52** | **1.62** | **-5.72** | **1.11E-07^*^** |
| **8370** | **Rafting** | **6.28** | **1.22** | **7.86** | **1.37** | **-6.19** | **1.31E-08^*^** |
| **8380** | **Athletes** | **6.2** | **0.88** | **7.88** | **1.22** | **-8.05** | **1.65E-12^*^** |
| **8400** | **Rafters** | **5.3** | **1.1** | **6.78** | **1.57** | **-5.58** | **2.06E-07^*^** |
| **8420** | **Tubing** | **5.87** | **1.19** | **7.9** | **1.5** | **-7.62** | **1.40E-11^*^** |
| **8460** | **Runner** | **5.53** | **1.02** | **7.2** | **1.6** | **-6.35** | **6.18E-09^*^** |
| 8461 | HappyTeens | 6.57 | 1.3 | 7.54 | 1.35 | -3.72 | 3.33E-04 |
| **8465** | **Runner** | **5.34** | **1.37** | **6.61** | **1.37** | **-4.7** | **8.33E-06^*^** |
| **8470** | **Gymnast** | **5.79** | **1.16** | **7.94** | **1.31** | **-8.84** | **3.12E-14^*^** |
| 8475 | Biking/train | 4.96 | 1.22 | 4.23 | 1.74 | 2.47 | 1.52E-02 |
| 8480 | BikerOnFire | 2.45 | 1.69 | 3.16 | 1.81 | -2.07 | 4.11E-02 |
| 8485 | Fire | 3.4 | 1.42 | 2.31 | 1.42 | 3.89 | 1.81E-04 |
| 8490 | RollerCoaster | 6.51 | 1.06 | 7.44 | 2.33 | -2.63 | 9.87E-03 |
| 8496 | WaterSlide | 7 | 1.18 | 7.94 | 1.75 | -3.21 | 1.76E-03 |
| **8497** | **CarnivalRide** | **5.96** | **1.06** | **7.7** | **1.22** | **-7.74** | **7.85E-12^*^** |
| 8500 | Gold | 6.53 | 1.25 | 7.16 | 1.55 | -2.27 | 2.53E-02 |
| 8501 | Money | 6.66 | 1.55 | 7.61 | 1.97 | -2.73 | 7.51E-03 |
| 8502 | Money | 6.36 | 1.41 | 7.65 | 1.78 | -4.09 | 8.63E-05 |
| **8503** | **Money** | **5.15** | **1.29** | **7.1** | **1.63** | **-6.75** | **9.40E-10^*^** |
| 8510 | SportCar | 6.06 | 1.21 | 7.11 | 1.81 | -3.47 | 7.57E-04 |
| 8531 | SportCar | 6 | 1.75 | 7.11 | 1.65 | -3.31 | 1.30E-03 |
| 8540 | Athletes | 6.6 | 1.37 | 7.66 | 1.44 | -3.85 | 2.10E-04 |
| 8600 | Mascot | 5.85 | 1.22 | 6.51 | 1.57 | -2.39 | 1.87E-02 |
| 8620 | Woman | 6.17 | 1.11 | 5.97 | 1.57 | 0.75 | 4.54E-01 |
| **9000** | **Cemetery** | **4.49** | **1.56** | **2.33** | **1.45** | **7.27** | **7.91E-11^*^** |
| 9001 | Cemetery | 3.55 | 1.4 | 2.82 | 1.88 | 2.26 | 2.62E-02 |
| 9005 | HIVTattoo | 3.83 | 1.81 | 3.23 | 2.07 | 1.57 | 1.18E-01 |
| **9006** | **HIVTattoo** | **3.51** | **1.47** | **2.08** | **1.52** | **4.85** | **4.49E-06^*^** |
| 9007 | Needles | 2.23 | 1.24 | 2.3 | 1.51 | -0.26 | 7.94E-01 |
| 9008 | Needle | 2.83 | 1.42 | 3.14 | 1.74 | -0.99 | 3.23E-01 |
| 9010 | BarbedWire | 4.04 | 1.22 | 4.17 | 1.78 | -0.43 | 6.65E-01 |
| 9040 | StarvingChild | 2.13 | 1.48 | 1.5 | 0.97 | 2.52 | 1.32E-02 |
| 9041 | ScareChild | 3.87 | 1.39 | 2.65 | 1.58 | 4.17 | 6.53E-05 |
| 9042 | StickThruLip | 2.28 | 1.48 | 2.44 | 1.5 | -0.56 | 5.78E-01 |
| 9045 | NativeFem | 3.17 | 1.56 | 3.59 | 1.98 | -1.2 | 2.34E-01 |
| 9046 | Family | 3.85 | 1.67 | 2.94 | 1.56 | 2.86 | 5.16E-03 |
| **9050** | **PlaneCrash** | **3.5** | **1.22** | **1.9** | **1.32** | **6.38** | **5.34E-09^*^** |
| 9070 | Boy | 4.47 | 1.41 | 5.13 | 1.85 | -2.05 | 4.31E-02 |
| 9080 | Wires | 3.57 | 1.21 | 4.08 | 1.33 | -2.02 | 4.62E-02 |
| 9090 | Exhaust | 4.96 | 1.35 | 4.02 | 1.98 | 2.82 | 5.78E-03 |
| 9101 | Cocaine | 4.69 | 1.31 | 3.4 | 1.85 | 4.1 | 8.50E-05 |
| 9102 | Heroin | 2.92 | 1.46 | 3.03 | 1.75 | -0.35 | 7.29E-01 |
| 9110 | Puddle | 3.53 | 1.2 | 3.75 | 1.44 | -0.84 | 4.04E-01 |
| 9120 | OilFires | 3.24 | 1.45 | 2.9 | 1.46 | 1.18 | 2.40E-01 |
| 9140 | Cow | 2.3 | 1.18 | 1.88 | 1.26 | 1.74 | 8.48E-02 |
| 9156 | Plane | 5.23 | 0.81 | 6.2 | 1.51 | -4.07 | 9.21E-05 |
| 9160 | Soldier | 3.79 | 1.12 | 2.81 | 1.66 | 3.52 | 6.51E-04 |
| 9171 | Fisher | 3.45 | 1.59 | 3.41 | 1.73 | 0.11 | 9.11E-01 |
| 9180 | Seal | 2.94 | 1.09 | 3.19 | 1.78 | -0.88 | 3.82E-01 |
| 9181 | DeadCows | 2.74 | 1.26 | 1.98 | 1.98 | 2.35 | 2.07E-02 |
| 9182 | Horses | 2.94 | 1.26 | 3.64 | 2.28 | -1.95 | 5.34E-02 |
| 9190 | Woman | 3.45 | 1 | 3.63 | 1.58 | -0.71 | 4.79E-01 |
| 9210 | Rain | 3.98 | 1.24 | 4.64 | 1.82 | -2.16 | 3.30E-02 |
| **9220** | **Cemetery** | **3.66** | **1.26** | **1.86** | **1.46** | **6.72** | **1.09E-09^*^** |
| 9230 | OilFire | 3.11 | 1.08 | 3.56 | 1.54 | -1.73 | 8.61E-02 |
| 9250 | WarVictim | 3 | 1.12 | 2.34 | 1.28 | 2.79 | 6.36E-03 |
| **9252** | **DeadBody** | **3.09** | **1.65** | **1.53** | **1.25** | **5.38** | **4.75E-07^*^** |
| 9253 | Mutilation | 2 | 0.88 | 1.6 | 0.99 | 2.16 | 3.28E-02 |
| 9265 | HungMan | 2.45 | 1.21 | 2.42 | 1.55 | 0.11 | 9.13E-01 |
| 9270 | ToxicWaste | 3.66 | 1.26 | 3.98 | 1.49 | -1.18 | 2.40E-01 |
| 9280 | Smoke | 3.32 | 1.3 | 2.69 | 1.47 | 2.31 | 2.31E-02 |
| 9290 | Garbage | 2.81 | 0.9 | 2.76 | 1.44 | 0.2 | 8.39E-01 |
| 9300 | Dirty | 1.87 | 0.95 | 1.83 | 1.37 | 0.18 | 8.55E-01 |
| 9301 | Toilet | 1.76 | 0.82 | 1.87 | 1.29 | -0.52 | 6.05E-01 |
| 9320 | Vomit | 2.17 | 1.2 | 2.26 | 1.82 | -0.3 | 7.67E-01 |
| 9330 | Garbage | 2.94 | 1.33 | 2.78 | 1.73 | 0.52 | 6.07E-01 |
| **9331** | **HomelessMan** | **4.6** | **0.98** | **2.67** | **1.27** | **8.65** | **8.15E-14^*^** |
| 9340 | Garbage | 2.89 | 1.15 | 2.43 | 1.49 | 1.76 | 8.15E-02 |
| 9341 | Pollution | 3.3 | 1.27 | 3.26 | 2.2 | 0.11 | 9.14E-01 |
| 9342 | Pollution | 2.81 | 1.28 | 2.53 | 1.3 | 1.09 | 2.77E-01 |
| 9360 | EmptyPool | 4.43 | 1.17 | 4.1 | 1.34 | 1.31 | 1.92E-01 |
| 9373 | Garbage | 2.4 | 0.97 | 3 | 1.51 | -2.4 | 1.84E-02 |
| **9390** | **Dishes** | **4.49** | **1.38** | **3.32** | **1.42** | **4.24** | **5.05E-05^*^** |
| 9400 | Soldier | 2.46 | 1.21 | 2.03 | 1.41 | 1.64 | 1.03E-01 |
| 9401 | Knives | 4.09 | 1.16 | 4.23 | 1.46 | -0.56 | 5.77E-01 |
| 9402 | Mob | 3.09 | 1.36 | 4.28 | 2.19 | -3.33 | 1.20E-03 |
| 9404 | Soldiers | 3.68 | 1.27 | 2.92 | 1.43 | 2.86 | 5.22E-03 |
| 9405 | SlicedHand | 1.83 | 0.84 | 1.59 | 1.02 | 1.3 | 1.95E-01 |
| 9409 | MenW/guns | 3.34 | 1.1 | 2.61 | 1.46 | 2.88 | 4.91E-03 |
| **9410** | **Soldier** | **1.94** | **0.92** | **1.2** | **0.58** | **4.83** | **4.82E-06^*^** |
| 9411 | Boy | 4.46 | 1.15 | 4.27 | 1.58 | 0.7 | 4.85E-01 |
| 9415 | Handicapped | 3.45 | 0.95 | 2.58 | 2.14 | 2.68 | 8.61E-03 |
| **9417** | **Ticket** | **4.68** | **0.96** | **2.95** | **1.45** | **7.19** | **1.17E-10^*^** |
| 9420 | Soldier | 2.13 | 1.08 | 1.87 | 1.54 | 0.99 | 3.25E-01 |
| **9421** | **Soldier** | **3.57** | **1.21** | **1.96** | **1.08** | **7.11** | **1.73E-10^*^** |
| 9430 | Burial | 2.91 | 1.08 | 2.3 | 1.57 | 2.32 | 2.21E-02 |
| **9432** | **Mastactomy** | **4.15** | **1.83** | **1.95** | **1.32** | **6.94** | **3.87E-10^*^** |
| **9433** | **DeadMan** | **2.51** | **1.1** | **1.35** | **0.71** | **6.32** | **7.24E-09^*^** |
| **9435** | **Accident** | **3.06** | **1.17** | **1.81** | **1.27** | **5.2** | **1.05E-06^*^** |
| 9440 | Skulls | 2.62 | 1.23 | 3.16 | 1.72 | -1.85 | 6.67E-02 |
| 9452 | Gun | 2.93 | 1.52 | 2.85 | 1.89 | 0.24 | 8.13E-01 |
| 9470 | Ruins | 3.49 | 1.28 | 2.58 | 1.6 | 3.2 | 1.86E-03 |
| 9471 | BurntBldg | 3.43 | 1.38 | 2.87 | 1.32 | 2.09 | 3.95E-02 |
| 9472 | Bridge | 4.09 | 1.3 | 3.9 | 1.31 | 0.73 | 4.68E-01 |
| 9480 | Skull | 2.85 | 1.37 | 3.12 | 2.39 | -0.71 | 4.82E-01 |
| 9490 | Corpse | 2.33 | 1.14 | 3.06 | 1.72 | -2.57 | 1.17E-02 |
| 9495 | Fire | 3.19 | 0.99 | 2.72 | 1.56 | 1.84 | 6.88E-02 |
| 9500 | Porpoises | 2.96 | 1.4 | 2.13 | 1.58 | 2.82 | 5.83E-03 |
| **9520** | **Kids** | **4.65** | **1.37** | **2.79** | **1.73** | **6.07** | **2.27E-08^*^** |
| 9530 | Boys | 3.34 | 1.71 | 2.26 | 1.5 | 3.4 | 9.68E-04 |
| **9560** | **DuckInOil** | **4.11** | **1.3** | **2.18** | **1.99** | **5.85** | **6.07E-08^*^** |
| **9561** | **SickKitty** | **5** | **2** | **2.21** | **1.66** | **7.68** | **1.05E-11^*^** |
| 9570 | Dog | 2.38 | 1.66 | 1.47 | 1 | 3.35 | 1.13E-03 |
| **9571** | **Cat** | **2.64** | **1.59** | **1.38** | **1.09** | **4.66** | **9.54E-06^*^** |
| 9582 | DentalExam | 3.72 | 1.16 | 4.24 | 2.64 | -1.3 | 1.97E-01 |
| 9584 | DentalExam | 3.55 | 1.23 | 3.29 | 1.55 | 0.96 | 3.41E-01 |
| 9592 | Injection | 3.74 | 1 | 2.88 | 1.67 | 3.19 | 1.90E-03 |
| 9594 | Injection | 3.91 | 1.08 | 3.56 | 1.9 | 1.17 | 2.44E-01 |
| **9600** | **Ship** | **3.06** | **1.19** | **1.9** | **1.18** | **4.99** | **2.55E-06^*^** |
| **9611** | **PlaneCrash** | **3.89** | **1.24** | **2.42** | **1.92** | **4.64** | **1.04E-05^*^** |
| **9620** | **Shipwreck** | **3.89** | **1.46** | **2.13** | **1.48** | **6.08** | **2.16E-08^*^** |
| **9621** | **Ship** | **4.91** | **0.98** | **2.95** | **1.64** | **7.4** | **4.12E-11^*^** |
| 9622 | Jet | 3.68 | 1.75 | 2.37 | 1.6 | 3.97 | 1.37E-04 |
| **9630** | **Bomb** | **5.06** | **1.44** | **2.96** | **1.5** | **7.27** | **7.74E-11^*^** |
| **9635.1** | **ManOnFire** | **3.6** | **1.74** | **1.43** | **0.89** | **7.88** | **3.92E-12^*^** |
| 9700 | Trash | 4.53 | 1.12 | 4.6 | 1.32 | -0.29 | 7.72E-01 |
| **9800** | **Skinhead** | **3.98** | **1.47** | **1.64** | **1.14** | **9** | **1.44E-14^*^** |
| **9810** | **KKKrally** | **3.26** | **1.37** | **1.94** | **1.71** | **4.34** | **3.45E-05^*^** |
| 9830 | Cigarettes | 2.94 | 1.28 | 2.42 | 1.88 | 1.64 | 1.04E-01 |
| **9910** | **CarAccident** | **3.45** | **1.25** | **1.8** | **1.14** | **6.98** | **3.16E-10^*^** |
| **9911** | **CarAccident** | **3.19** | **1.01** | **1.88** | **1.16** | **6.12** | **1.81E-08^*^** |
| 9912 | Firefigher | 3.42 | 1.1 | 3.51 | 1.91 | -0.3 | 7.69E-01 |
| 9913 | Truck | 3.91 | 1.53 | 3.93 | 1.95 | -0.04 | 9.65E-01 |
| 9920 | CarAccident | 2.85 | 0.93 | 2.37 | 1.62 | 1.86 | 6.58E-02 |
| **9921** | **Fire** | **3.68** | **1.53** | **1.58** | **1.06** | **8.05** | **1.67E-12^*^** |

^#^ The data about American participants were collected by Lang.

*P<6×10^-5^
