# Supplementary material for: Affective reactions differ between Chinese and American healthy young adults: a cross-cultural study using the international affective picture system
Source: BMC Psychiatry. 2015 Mar 27;15:60. doi: 10.1186/s12888-015-0442-9 (PMC4378560; doi:10.1186/s12888-015-0442-9)
Supplement: Additional file 4: Table S3. — Valence scores of Chinese and American male participants. [file 12888_2015_442_MOESM4_ESM.doc]

Table S3. Valence scores of Chinese and American male participants

| No. | description | China | | America^#^ | | t | p |
| --- | --- | --- | --- | --- | --- | --- | --- |
|  |  | mean | SD | mean | SD |  |  |
| 1019 | Snake | 4.31 | 2.14 | 4.67 | 1.63 | -0.99 | 3.23E-01 |
| 1022 | Snake | 4.05 | 2.82 | 4.48 | 1.62 | -0.97 | 3.36E-01 |
| 1030 | Snake | 3.48 | 1.46 | 4.7 | 2.55 | -3.27 | 1.44E-03 |
| 1040 | Snake | 3.48 | 1.59 | 4.38 | 2.31 | -2.5 | 1.40E-02 |
| 1050 | Snake | 3.49 | 2.13 | 3.9 | 2.28 | -1 | 3.20E-01 |
| 1051 | Snake | 4.34 | 1.56 | 4.53 | 1.6 | -0.64 | 5.20E-01 |
| 1052 | Snake | 4.02 | 1.71 | 4.35 | 1.56 | -1.07 | 2.86E-01 |
| 1070 | Snake | 3.79 | 1.36 | 4.43 | 2.42 | -1.82 | 7.20E-02 |
| 1080 | Snake | 3.66 | 1.49 | 4.9 | 2.32 | -3.51 | 6.35E-04 |
| 1090 | Snake | 3.79 | 1.58 | 4.17 | 1.79 | -1.22 | 2.27E-01 |
| **1101** | **Snake** | **3.74** | **1.38** | **5.11** | **1.37** | **-5.33** | **4.99E-07^*^** |
| 1110 | Snake | 3.9 | 1.6 | 4.07 | 2.03 | -0.51 | 6.13E-01 |
| **1111** | **Snake** | **2.25** | **1.15** | **3.76** | **1.52** | **-6.12** | **1.34E-08^*^** |
| 1112 | Snake | 4.38 | 1.24 | 4.83 | 1.79 | -1.61 | 1.11E-01 |
| 1113 | Snake | 3.52 | 2.05 | 4.37 | 1.72 | -2.37 | 1.93E-02 |
| 1114 | Snake | 3.43 | 1.62 | 4.73 | 1.93 | -3.96 | 1.33E-04 |
| 1120 | Snake | 3.56 | 1.81 | 4.73 | 1.75 | -3.51 | 6.45E-04 |
| 1121 | Lizard | 5.39 | 1.7 | 6.02 | 1.23 | -2.22 | 2.83E-02 |
| 1200 | Spider | 4.03 | 1.4 | 4.46 | 2.13 | -1.32 | 1.91E-01 |
| **1201** | **Spider** | **2.8** | **1.22** | **4.27** | **1.73** | **-5.39** | **3.80E-07^*^** |
| 1205 | Spider | 3.84 | 1.63 | 4.15 | 1.78 | -0.98 | 3.30E-01 |
| 1220 | Spider | 4.08 | 1.45 | 3.88 | 1.76 | 0.67 | 5.02E-01 |
| 1230 | Spider | 5.03 | 1.62 | 4.95 | 1.56 | 0.27 | 7.89E-01 |
| 1240 | Spider | 4.3 | 1.57 | 4.7 | 1.81 | -1.28 | 2.04E-01 |
| 1270 | Roach | 3.51 | 1.52 | 4.58 | 1.65 | -3.63 | 4.22E-04 |
| 1274 | Roaches | 3.49 | 1.64 | 3.5 | 1.44 | -0.03 | 9.73E-01 |
| 1275 | Roaches | 3.67 | 1.54 | 3.53 | 1.61 | 0.48 | 6.34E-01 |
| **1280** | **Rat** | **2.72** | **1.11** | **4.7** | **1.7** | **-7.61** | **8.26E-12^*^** |
| 1300 | PitBull | 3.31 | 1.4 | 4.06 | 1.54 | -2.75 | 7.00E-03 |
| 1301 | Dog | 3.87 | 1.59 | 4.1 | 1.71 | -0.75 | 4.55E-01 |
| 1302 | Dog | 4.44 | 1.42 | 4.38 | 1.64 | 0.21 | 8.33E-01 |
| 1303 | Dog | 5.1 | 1.49 | 4.72 | 1.94 | 1.2 | 2.33E-01 |
| **1310** | **Leopard** | **4.1** | **1.72** | **5.92** | **1.54** | **-5.92** | **3.44E-08^*^** |
| **1313** | **Frog** | **3.95** | **1.49** | **5.46** | **1.25** | **-5.8** | **5.91E-08^*^** |
| 1321 | Bear | 4.98 | 1.61 | 4.94 | 1.71 | 0.13 | 8.97E-01 |
| 1333 | Parrots | 5.8 | 1.36 | 5.79 | 1.21 | 0.04 | 9.67E-01 |
| 1340 | Women | 5.46 | 1.22 | 6.31 | 1.27 | -3.66 | 3.78E-04 |
| 1390 | Bees | 4.69 | 1.54 | 5 | 1.43 | -1.11 | 2.69E-01 |
| 1419 | Bird | 5.72 | 1.16 | 6.08 | 1.13 | -1.68 | 9.59E-02 |
| **1440** | **Seal** | **6.54** | **1.37** | **7.96** | **1.59** | **-5.18** | **9.70E-07^*^** |
| 1450 | Gannet | 6.42 | 1.06 | 5.84 | 1.56 | 2.39 | 1.83E-02 |
| **1460** | **Kitten** | **6.18** | **1.51** | **7.8** | **1.47** | **-5.81** | **5.78E-08^*^** |
| 1463 | Kittens | 7.1 | 1.09 | 7.1 | 1.47 | 0 | 1.00E+00 |
| **1500** | **Dog** | **5.18** | **1.32** | **6.77** | **1.95** | **-5.26** | **6.86E-07^*^** |
| **1510** | **Dog** | **4.74** | **1.37** | **6.88** | **1.79** | **-7.33** | **3.50E-11^*^** |
| 1525 | AttackDog | 3.67 | 1.86 | 3.55 | 1.59 | 0.37 | 7.14E-01 |
| 1540 | Cat | 5.85 | 1.21 | 6.83 | 1.87 | -3.44 | 8.24E-04 |
| 1560 | Hawk | 5.74 | 1.32 | 6.55 | 2.32 | -2.39 | 1.86E-02 |
| 1590 | Horse | 5.7 | 1.32 | 6.68 | 1.75 | -3.45 | 7.74E-04 |
| **1600** | **Horse** | **5.43** | **1.2** | **6.95** | **1.59** | **-5.89** | **3.83E-08^*^** |
| 1601 | Giraffes | 5.9 | 1.21 | 6.54 | 1.46 | -2.59 | 1.08E-02 |
| 1602 | Butterfly | 6.31 | 1.37 | 5.92 | 1.38 | 1.52 | 1.32E-01 |
| 1603 | Butterfly | 5.97 | 1.26 | 6.33 | 1.55 | -1.38 | 1.69E-01 |
| 1604 | Butterfly | 6.1 | 1.22 | 6.4 | 1.31 | -1.27 | 2.05E-01 |
| 1610 | Rabbit | 6.57 | 1.15 | 7.32 | 1.42 | -3.15 | 2.05E-03 |
| 1616 | Bird | 4.89 | 1.16 | 5.34 | 0.92 | -2.26 | 2.56E-02 |
| 1620 | Sprgbok | 5.72 | 1.13 | 6.8 | 1.68 | -4.15 | 6.31E-05 |
| 1640 | Coyote | 5.69 | 1.16 | 6.23 | 1.86 | -1.93 | 5.63E-02 |
| 1650 | Jaguar | 5.15 | 1.39 | 6.08 | 2.14 | -2.84 | 5.26E-03 |
| **1660** | **Gorilla** | **4.77** | **1.63** | **6.65** | **1.96** | **-5.66** | **1.14E-07^*^** |
| 1661 | Orangutan | 5.26 | 1.05 | 5.19 | 1.39 | 0.31 | 7.57E-01 |
| 1670 | Cow | 5.49 | 1.63 | 6.51 | 1.76 | -3.24 | 1.58E-03 |
| **1710** | **Puppies** | **6.89** | **1.42** | **8.02** | **1.21** | **-4.53** | **1.45E-05^*^** |
| **1720** | **Lion** | **5.57** | **1.26** | **6.81** | **1.53** | **-4.8** | **4.76E-06^*^** |
| 1721 | Lion | 6.02 | 1.5 | 6.85 | 1.38 | -3.06 | 2.73E-03 |
| 1722 | Jaguars | 5.95 | 1.28 | 6.85 | 1.55 | -3.44 | 8.21E-04 |
| 1726 | Tiger | 4.85 | 1.64 | 5.34 | 1.94 | -1.48 | 1.42E-01 |
| 1731 | Lion | 6.69 | 1.36 | 6.84 | 1.48 | -0.57 | 5.71E-01 |
| **1740** | **Owl** | **5.34** | **1.12** | **6.81** | **1.46** | **-6.16** | **1.08E-08^*^** |
| **1750** | **Bunnies** | **6.34** | **1.17** | **7.89** | **1.26** | **-6.86** | **3.68E-10^*^** |
| 1810 | Hippo | 5.89 | 1.34 | 6.35 | 1.4 | -1.8 | 7.42E-02 |
| 1811 | Monkies | 6.23 | 2.04 | 7.22 | 1.59 | -2.85 | 5.25E-03 |
| 1812 | Elephants | 6.13 | 1.34 | 6.3 | 1.25 | -0.7 | 4.86E-01 |
| 1850 | Camels | 5.28 | 1.24 | 6.13 | 1.31 | -3.58 | 5.04E-04 |
| 1900 | Fish | 6.23 | 1.43 | 6.4 | 1.67 | -0.59 | 5.55E-01 |
| 1910 | Grouper | 5.36 | 1.58 | 6.46 | 1.87 | -3.44 | 8.03E-04 |
| **1920** | **Porpoise** | **6.26** | **1.14** | **7.83** | **1.29** | **-6.96** | **2.18E-10^*^** |
| 1930 | Shark | 4.28 | 1.39 | 4.12 | 1.92 | 0.52 | 6.02E-01 |
| 1931 | Shark | 4.85 | 1.67 | 4.51 | 2.35 | 0.91 | 3.62E-01 |
| 1932 | Shark | 4.07 | 1.64 | 4.85 | 1.87 | -2.4 | 1.82E-02 |
| 1935 | HermitCrab | 4.57 | 1.42 | 5.17 | 1.32 | -2.33 | 2.16E-02 |
| 1942 | Tuetle | 5.56 | 1.77 | 6.15 | 1.49 | -1.91 | 5.92E-02 |
| **1945** | **Tuetles** | **3.82** | **1.54** | **5.21** | **1.53** | **-4.84** | **4.02E-06^*^** |
| 1947 | Octopus | 6.18 | 1.29 | 6.04 | 1.3 | 0.58 | 5.64E-01 |
| **1999** | **Mickey** | **6.07** | **1.09** | **7.17** | **1.4** | **-4.78** | **5.25E-06^*^** |
| 2000 | Adult | 5.52 | 1.43 | 5.93 | 1.86 | -1.35 | 1.80E-01 |
| 2005 | AttractiveMan | 5.38 | 0.99 | 5.19 | 1.94 | 0.69 | 4.91E-01 |
| 2010 | Adult | 5 | 1.37 | 5.75 | 1.71 | -2.63 | 9.63E-03 |
| 2020 | Adult | 5.18 | 1.87 | 5.4 | 1.82 | -0.64 | 5.26E-01 |
| 2025 | Women | 5.25 | 1.5 | 6.17 | 1.19 | -3.58 | 5.11E-04 |
| **2030** | **Women** | **6.26** | **1.06** | **7.51** | **1.68** | **-4.92** | **2.90E-06^*^** |
| 2040 | Baby | 6.53 | 1.54 | 7.63 | 2.01 | -3.35 | 1.08E-03 |
| 2050 | Baby | 6.52 | 2.16 | 7.8 | 1.54 | -3.57 | 5.29E-04 |
| 2053 | Baby | 2.85 | 1.71 | 2.78 | 1.8 | 0.21 | 8.31E-01 |
| 2055.1 | ManInPool | 3.39 | 1.6 | 3.51 | 1.74 | -0.39 | 7.00E-01 |
| 2057 | Father | 7.25 | 1.39 | 7.16 | 1.31 | 0.36 | 7.23E-01 |
| **2058** | **Baby** | **6.18** | **1.19** | **7.34** | **1.37** | **-4.89** | **3.33E-06^*^** |
| 2070 | Baby | 7.1 | 1.5 | 7.69 | 1.59 | -2.05 | 4.25E-02 |
| 2071 | Baby | 6.95 | 1.28 | 7.45 | 1.24 | -2.12 | 3.63E-02 |
| 2080 | Babies | 6.41 | 1.39 | 7.56 | 1.66 | -4.07 | 8.57E-05 |
| **2091** | **Girls** | **6.77** | **1.19** | **5.49** | **1.49** | **5.16** | **1.02E-06^*^** |
| 2092 | Clowns | 5.52 | 1.43 | 5.76 | 1.55 | -0.87 | 3.88E-01 |
| 2095 | Toddler | 2.89 | 1.39 | 2.16 | 1.31 | 2.88 | 4.75E-03 |
| 2100 | AngryFace | 3.69 | 1.82 | 4.3 | 1.95 | -1.74 | 8.46E-02 |
| 2110 | AngryFace | 3.72 | 1.43 | 3.98 | 1.64 | -0.91 | 3.63E-01 |
| 2120 | AngryFace | 3.03 | 1.63 | 3.65 | 2.05 | -1.82 | 7.11E-02 |
| 2130 | Women | 3.2 | 1.31 | 4.2 | 1.45 | -3.9 | 1.61E-04 |
| 2141 | GrievingFem | 3.52 | 1.41 | 2.72 | 1.39 | 3.05 | 2.80E-03 |
| **2150** | **Baby** | **6.1** | **1.26** | **7.46** | **1.6** | **-5.14** | **1.12E-06^*^** |
| 2160 | Father | 6.31 | 1.51 | 6.87 | 1.87 | -1.79 | 7.59E-02 |
| 2165 | Father | 7.07 | 1.28 | 6.74 | 1.39 | 1.33 | 1.86E-01 |
| 2170 | Mother | 6.43 | 1.1 | 7.35 | 1.45 | -3.9 | 1.60E-04 |
| 2190 | Man | 4.75 | 0.75 | 4.73 | 1.25 | 0.11 | 9.14E-01 |
| 2191 | Farmer | 5.11 | 1.08 | 5.49 | 1.49 | -1.6 | 1.12E-01 |
| 2200 | NeutFace | 5.08 | 1.01 | 4.61 | 1.18 | 2.32 | 2.23E-02 |
| **2205** | **Hospital** | **3.62** | **1.2** | **2.24** | **1.93** | **4.75** | **5.84E-06^*^** |
| 2206 | Fingerprint | 4.67 | 1.11 | 3.91 | 1.51 | 3.14 | 2.16E-03 |
| **2208** | **Bride** | **5.59** | **1.2** | **7.02** | **1.72** | **-5.3** | **5.74E-07^*^** |
| **2209** | **Bride** | **5.42** | **1.15** | **7.12** | **1.33** | **-7.4** | **2.47E-11^*^** |
| 2210 | NeutFace | 4.89 | 1.67 | 4.81 | 0.87 | 0.31 | 7.58E-01 |
| 2214 | NeutFace | 5.11 | 0.8 | 4.83 | 0.99 | 1.69 | 9.35E-02 |
| 2215 | NeutFace | 4.79 | 1.14 | 4.79 | 1.02 | 0 | 1.00E+00 |
| **2216** | **Chidren** | **6.02** | **0.97** | **7.12** | **1.41** | **-5** | **2.09E-06^*^** |
| **2220** | **MaleFace** | **4.18** | **1.22** | **5.21** | **1.34** | **-4.33** | **3.19E-05^*^** |
| 2221 | Judge | 4.36 | 1.24 | 4.74 | 1.2 | -1.66 | 9.91E-02 |
| **2222** | **BoysReading** | **5.43** | **1.16** | **6.55** | **1.34** | **-4.83** | **4.19E-06^*^** |
| **2224** | **Boys** | **5.53** | **1.23** | **6.78** | **1.35** | **-5.22** | **8.20E-07^*^** |
| 2230 | SadFace | 4.44 | 0.99 | 4.67 | 1.06 | -1.21 | 2.30E-01 |
| 2235 | Butcher | 5 | 0.93 | 5.47 | 1.17 | -2.42 | 1.71E-02 |
| 2240 | NeutChild | 5.9 | 1.27 | 5.91 | 1.38 | -0.04 | 9.68E-01 |
| 2250 | NeutBaby | 5.66 | 1 | 5.8 | 2.37 | -0.43 | 6.64E-01 |
| **2260** | **NeutBaby** | **6.05** | **1.15** | **7.63** | **1.6** | **-6.22** | **8.43E-09^*^** |
| 2270 | NeutChild | 4.83 | 1.22 | 5.39 | 1.04 | -2.61 | 1.02E-02 |
| 2271 | Women | 3.51 | 1.26 | 4.34 | 1.05 | -3.78 | 2.51E-04 |
| 2272 | LonelyBoy | 4.57 | 1.4 | 4.51 | 1.43 | 0.23 | 8.21E-01 |
| 2276 | Girl | 3.89 | 1.47 | 3.17 | 1.56 | 2.55 | 1.20E-02 |
| 2278 | Kids | 4.08 | 1.29 | 3.39 | 1.34 | 2.82 | 5.74E-03 |
| 2280 | Boy | 4.89 | 0.93 | 4.57 | 1.16 | 1.66 | 1.01E-01 |
| **2299** | **Family** | **5.48** | **1.06** | **6.75** | **1.38** | **-5.63** | **1.29E-07^*^** |
| 2303 | Children | 5.66 | 1.15 | 6.51 | 2.01 | -2.89 | 4.63E-03 |
| **2304** | **Girl** | **5.02** | **1.49** | **6.42** | **1.23** | **-5.41** | **3.51E-07^*^** |
| 2310 | Mother | 6.07 | 1.03 | 6.61 | 1.24 | -2.57 | 1.14E-02 |
| 2311 | Mother | 6.62 | 1.24 | 7.24 | 1.48 | -2.46 | 1.53E-02 |
| 2312 | Mother | 4.48 | 1.03 | 4 | 1.35 | 2.18 | 3.11E-02 |
| 2320 | Girl | 5.31 | 1.47 | 5.45 | 1.33 | -0.53 | 5.97E-01 |
| 2331 | Chef | 6.1 | 1.22 | 6.6 | 1.73 | -1.83 | 6.93E-02 |
| **2340** | **Family** | **6.1** | **1.21** | **7.65** | **1.36** | **-6.5** | **2.16E-09^*^** |
| 2341 | Children | 6.64 | 1 | 6.78 | 1.41 | -0.63 | 5.31E-01 |
| 2344 | Children | 5.59 | 1.46 | 6.06 | 1.55 | -1.68 | 9.61E-02 |
| **2345** | **Children** | **5.52** | **1.61** | **6.91** | **1.59** | **-4.64** | **9.14E-06^*^** |
| **2346** | **Kids** | **5.49** | **1.62** | **7.09** | **1.32** | **-5.71** | **9.00E-08^*^** |
| **2351** | **NursingBaby** | **4.32** | **1.59** | **5.48** | **1.92** | **-3.57** | **5.21E-04^*^** |
| **2352** | **Kiss** | **4.95** | **1.49** | **6.5** | **1.71** | **-5.22** | **7.87E-07^*^** |
| 2352.1 | Kiss | 5.97 | 1.11 | 6.77 | 1.81 | -2.95 | 3.83E-03 |
| 2352.2 | BloodyKiss | 2.08 | 1.24 | 2.41 | 1.59 | -1.26 | 2.10E-01 |
| **2357** | **Man** | **4.23** | **0.99** | **5.33** | **1.24** | **-5.33** | **4.87E-07^*^** |
| 2360 | Family | 6.05 | 1.53 | 6.98 | 1.76 | -3.05 | 2.85E-03 |
| 2370 | ThreeMan | 5.64 | 1.56 | 6.71 | 1.32 | -3.91 | 1.54E-04 |
| 2372 | Woman | 4.46 | 0.92 | 5.35 | 1.49 | -3.98 | 1.21E-04 |
| 2375.1 | Woman | 3.52 | 1.47 | 2.55 | 1.37 | 3.63 | 4.18E-04 |
| 2381 | Girl | 4.97 | 1.63 | 5.54 | 0.96 | -2.2 | 2.96E-02 |
| 2383 | Secretary | 4.64 | 1.08 | 4.62 | 1.24 | 0.09 | 9.26E-01 |
| 2385 | Girl | 4.66 | 1.52 | 5.27 | 1.45 | -2.19 | 3.06E-02 |
| 2387 | Kids | 6.15 | 1.19 | 6.52 | 1.33 | -1.58 | 1.16E-01 |
| 2388 | Kids | 5.73 | 1.33 | 6.73 | 1.4 | -3.93 | 1.43E-04 |
| **2389** | **Teens** | **5.05** | **1.07** | **6.38** | **1.26** | **-6.16** | **1.09E-08^*^** |
| 2391 | Boy | 6.38 | 1.18 | 7.33 | 1.86 | -3.37 | 1.02E-03 |
| 2393 | Factoryworker | 4.98 | 0.79 | 4.82 | 1.08 | 0.93 | 3.56E-01 |
| 2394 | Medicalworker | 5.15 | 0.91 | 5.36 | 1.29 | -1.03 | 3.04E-01 |
| 2395 | Family | 5.59 | 1.05 | 6.62 | 1.76 | -3.94 | 1.38E-04 |
| 2399 | Woman | 3.57 | 0.87 | 3.9 | 1.15 | -1.77 | 7.98E-02 |
| 2410 | Boy | 4.95 | 1.4 | 4.72 | 1.46 | 0.86 | 3.90E-01 |
| **2435** | **Mom/Son** | **4.79** | **1.05** | **5.7** | **1.24** | **-4.29** | **3.73E-05^*^** |
| 2440 | NeutGirl | 4.9 | 1.46 | 4.44 | 1.08 | 1.88 | 6.31E-02 |
| 2441 | NeutralGirl | 4.26 | 1.14 | 4.95 | 0.9 | -3.53 | 5.89E-04 |
| 2442 | DryingHair | 5.74 | 1.44 | 5.78 | 1.09 | -0.16 | 8.70E-01 |
| 2455 | SadGirls | 3.97 | 1.4 | 3.32 | 1.54 | 2.38 | 1.89E-02 |
| 2480 | ElderlyMan | 5.11 | 1.52 | 4.76 | 1.23 | 1.33 | 1.85E-01 |
| 2485 | Man | 4.56 | 1.12 | 5.33 | 1.39 | -3.32 | 1.22E-03 |
| 2487 | Musician | 5.21 | 0.99 | 5.09 | 1.41 | 0.54 | 5.90E-01 |
| 2490 | Man | 4.34 | 1.05 | 3.96 | 1.93 | 1.36 | 1.75E-01 |
| 2491 | SickMan | 4.56 | 1.05 | 4.33 | 0.94 | 1.23 | 2.23E-01 |
| 2493 | NeutralMale | 5.23 | 0.96 | 4.52 | 1.08 | 3.75 | 2.78E-04 |
| 2495 | Man | 4.93 | 0.85 | 5.35 | 0.86 | -2.63 | 9.69E-03 |
| 2499 | NeutralMale | 4.62 | 1.24 | 5.33 | 1.01 | -3.31 | 1.24E-03 |
| 2500 | Man | 6.13 | 1.12 | 5.83 | 1.56 | 1.21 | 2.28E-01 |
| 2501 | Couple | 6.1 | 1.01 | 6.33 | 1.86 | -0.86 | 3.93E-01 |
| **2510** | **ElderlyWoman** | **5.27** | **1.55** | **6.66** | **1.91** | **-4.34** | **3.05E-05^*^** |
| 2514 | Woman | 4.95 | 0.9 | 5.15 | 0.85 | -1.22 | 2.26E-01 |
| 2515 | Harvest | 5.2 | 1.06 | 5.7 | 1.44 | -2.16 | 3.25E-02 |
| 2516 | ElderlyWoman | 4.95 | 1.18 | 5.11 | 0.99 | -0.78 | 4.39E-01 |
| 2518 | Quilting | 4.64 | 1.27 | 5.38 | 1.57 | -2.82 | 5.71E-03 |
| 2520 | ElderlyMan | 4.43 | 1.17 | 4.12 | 1.99 | 1.05 | 2.94E-01 |
| 2530 | Couple | 6.13 | 1.09 | 7.25 | 1.84 | -4.11 | 7.40E-05 |
| 2540 | Mother | 7.18 | 1.34 | 7.23 | 1.57 | -0.19 | 8.53E-01 |
| 2550 | Couple | 6.75 | 1.3 | 7.37 | 1.2 | -2.64 | 9.54E-03 |
| 2560 | Picnic | 5.2 | 1.67 | 6.02 | 1.55 | -2.71 | 7.79E-03 |
| 2570 | Man | 4.21 | 1.34 | 4.6 | 1.37 | -1.54 | 1.26E-01 |
| **2575** | **Propeller** | **4.93** | **0.79** | **5.69** | **1.13** | **-4.28** | **3.87E-05^*^** |
| 2579 | Bakers | 5.59 | 1.19 | 5.7 | 1.2 | -0.49 | 6.23E-01 |
| 2580 | Chess | 5.03 | 1.29 | 5.45 | 1.33 | -1.72 | 8.83E-02 |
| **2590** | **ElderlyWoman** | **4.15** | **1.24** | **3.04** | **1.48** | **4.41** | **2.36E-05^*^** |
| 2595 | Women | 4.39 | 1.13 | 4.79 | 1.15 | -1.88 | 6.27E-02 |
| 2600 | Beer | 4.93 | 1.35 | 5.92 | 2.1 | -3.1 | 2.45E-03 |
| **2616** | **Dancer** | **4.74** | **1.52** | **6.13** | **1.33** | **-5.16** | **1.05E-06^*^** |
| 2620 | Woman | 6.52 | 1.42 | 5.73 | 1.35 | 3.04 | 2.93E-03 |
| 2630 | Male | 5.31 | 1.16 | 4.88 | 1.38 | 1.83 | 7.01E-02 |
| 2635 | Cowboy | 4.57 | 1.16 | 5.26 | 1.61 | -2.7 | 8.09E-03 |
| 2650 | Boy | 6.66 | 1.25 | 6.82 | 1.91 | -0.55 | 5.86E-01 |
| 2655 | Child | 6.53 | 1.29 | 6.62 | 1.47 | -0.35 | 7.26E-01 |
| 2660 | Baby | 6.31 | 1.22 | 7.28 | 1.59 | -3.73 | 2.94E-04 |
| 2661 | Baby | 3.07 | 1.45 | 3.22 | 2.01 | -0.47 | 6.40E-01 |
| 2681 | Police | 4.26 | 1.54 | 3.8 | 1.42 | 1.65 | 1.01E-01 |
| 2682 | Police | 3.8 | 1.67 | 3.98 | 1.97 | -0.53 | 5.94E-01 |
| 2683 | War | 3.66 | 1.18 | 3.32 | 1.67 | 1.29 | 1.99E-01 |
| **2688** | **Hunters** | **5.15** | **1.59** | **3.18** | **2** | **5.93** | **3.18E-08^*^** |
| **2690** | **Terrorist** | **3.79** | **1.7** | **5.08** | **1.27** | **-4.51** | **1.59E-05^*^** |
| 2691 | Riot | 4.15 | 1.42 | 3.9 | 1.81 | 0.84 | 4.04E-01 |
| 2692 | Bomb | 4.18 | 1.13 | 4.02 | 1.53 | 0.65 | 5.16E-01 |
| 2694 | Police | 3.69 | 1.1 | 4.18 | 1.71 | -1.88 | 6.23E-02 |
| 2695 | Refugees | 4.2 | 0.19 | 4.49 | 1.23 | -1.9 | 5.96E-02 |
| 2700 | Woman | 3.48 | 1.15 | 3.33 | 1.87 | 0.54 | 5.94E-01 |
| 2702 | BingeEating | 6.07 | 1.67 | 5.78 | 1.59 | 0.95 | 3.45E-01 |
| 2710 | DrugAddict | 3.08 | 1.22 | 3.04 | 1.83 | 0.14 | 8.88E-01 |
| 2715 | Smoking | 4.02 | 1.3 | 3.6 | 1.71 | 1.51 | 1.34E-01 |
| **2720** | **Urinating** | **3.84** | **1.67** | **5.71** | **1.53** | **-6.21** | **8.77E-09^*^** |
| 2722 | Jail | 3.56 | 1.25 | 4.2 | 1.42 | -2.58 | 1.10E-02 |
| 2730 | NativeBoy | 3.51 | 2.19 | 3.4 | 2.92 | 0.23 | 8.16E-01 |
| 2745.1 | Shopping | 4.92 | 0.92 | 5.22 | 0.89 | -1.77 | 7.95E-02 |
| 2749 | Smoking | 4.77 | 0.96 | 5.15 | 1.18 | -1.92 | 5.74E-02 |
| 2750 | Bum | 2.83 | 1.42 | 2.57 | 1.46 | 0.97 | 3.35E-01 |
| 2751 | DrunkDriving | 3.55 | 1.47 | 3.49 | 2.04 | 0.18 | 8.54E-01 |
| 2752 | Alcoholic | 3.31 | 1.18 | 4.15 | 1.57 | -3.31 | 1.27E-03 |
| 2753 | Alcoholic | 4.16 | 1.42 | 3.73 | 1.65 | 1.51 | 1.33E-01 |
| **2780** | **Actor** | **2.98** | **1.52** | **4.75** | **1.33** | **-6.57** | **1.55E-09^*^** |
| 2791 | Balloons | 5.68 | 1.11 | 6.02 | 1.8 | -1.26 | 2.11E-01 |
| 2795 | Boy | 3.79 | 1.18 | 4.09 | 1.57 | -1.18 | 2.40E-01 |
| 2800 | SadChild | 2.07 | 1.22 | 2.31 | 1.36 | -1 | 3.18E-01 |
| 2810 | Boy | 4.02 | 1.35 | 4.56 | 1.74 | -1.89 | 6.12E-02 |
| 2830 | Woman | 4.57 | 1.72 | 5.09 | 1.41 | -1.74 | 8.38E-02 |
| 2840 | Chess | 5.56 | 0.91 | 4.92 | 1.79 | 2.52 | 1.30E-02 |
| 2850 | Tourist | 4.79 | 1.11 | 4.69 | 1.4 | 0.43 | 6.67E-01 |
| 2870 | Teenager | 4.95 | 0.9 | 5.17 | 0.94 | -1.28 | 2.02E-01 |
| **2880** | **Shadow** | **4.39** | **1.05** | **5.13** | **0.77** | **-4.21** | **5.13E-05^*^** |
| 2890 | Twins | 4.54 | 1.63 | 4.87 | 1.08 | -1.24 | 2.17E-01 |
| **2900** | **CryingBoy** | **3.9** | **1.11** | **2.76** | **1.25** | **5.21** | **8.53E-07^*^** |
| 2900.1 | FoodBasket | 3.9 | 1.19 | 3.26 | 1.33 | 2.74 | 7.20E-03 |
| 2900.2 | DeerHead | 5.85 | 1.33 | 6.21 | 1.53 | -1.36 | 1.77E-01 |
| 2980 | Mutilation | 4.74 | 1.22 | 5.48 | 1.19 | -3.28 | 1.38E-03 |
| 2981 | Mutilation | 3.7 | 1.68 | 3.55 | 1.89 | 0.45 | 6.52E-01 |
| 3000 | Mutilation | 1.3 | 0.82 | 1.69 | 1.47 | -1.83 | 7.05E-02 |
| 3005.1 | OpenGrave | 1.98 | 1.13 | 1.96 | 1.44 | 0.08 | 9.33E-01 |
| 3010 | Mutilation | 1.61 | 1.44 | 2.2 | 1.36 | -2.24 | 2.67E-02 |
| 3015 | Accident | 2.16 | 1.52 | 1.83 | 1.19 | 1.27 | 2.06E-01 |
| 3022 | Scream | 3.79 | 1.42 | 4.28 | 1.6 | -1.75 | 8.30E-02 |
| 3030 | Mutilation | 2.49 | 1.07 | 2.31 | 1.87 | 0.66 | 5.12E-01 |
| 3051 | Mutilation | 1.92 | 1.28 | 2.56 | 1.74 | -2.29 | 2.37E-02 |
| 3053 | BurnVictim | 3.13 | 10.37 | 1.5 | 1.16 | 1.11 | 2.71E-01 |
| 3060 | Mutilation | 1.59 | 0.94 | 1.94 | 1.39 | -1.62 | 1.07E-01 |
| **3061** | **Mutilation** | **1.44** | **0.94** | **2.79** | **1.65** | **-5.59** | **1.52E-07^*^** |
| 3062 | Mutilation | 1.45 | 1.03 | 2.21 | 1.41 | -3.37 | 1.02E-03 |
| 3063 | Mutilation | 1.84 | 1.58 | 1.84 | 1.12 | 0 | 1.00E+00 |
| 3064 | Mutilation | 1.44 | 0.83 | 1.78 | 1.26 | -1.76 | 8.15E-02 |
| 3068 | Mutilation | 2.07 | 1.35 | 2.47 | 1.92 | -1.32 | 1.88E-01 |
| 3069 | Mutilation | 1.68 | 1.1 | 2.1 | 1.66 | -1.64 | 1.03E-01 |
| 3071 | Mutilation | 1.62 | 0.86 | 2.06 | 1.59 | -1.92 | 5.72E-02 |
| 3080 | Mutilation | 2.27 | 1.44 | 1.63 | 1.11 | 2.61 | 1.01E-02 |
| 3100 | BurnVictim | 1.84 | 0.93 | 1.88 | 1.14 | -0.21 | 8.35E-01 |
| 3102 | BurnVictim | 1.49 | 1.12 | 1.62 | 1.39 | -0.56 | 5.77E-01 |
| 3110 | BurnVictim | 1.56 | 1.15 | 2.1 | 1.56 | -2.16 | 3.32E-02 |
| 3120 | DeadBoy | 1.85 | 1.34 | 1.8 | 1.32 | 0.2 | 8.41E-01 |
| 3130 | Mutilation | 1.98 | 0.99 | 1.9 | 1.57 | 0.34 | 7.37E-01 |
| 3140 | DeadBoy | 2.03 | 1.35 | 2.22 | 1.27 | -0.77 | 4.42E-01 |
| 3150 | Mutilation | 1.77 | 1.06 | 2.59 | 1.56 | -3.38 | 9.79E-04 |
| 3160 | EyeDisease | 2.41 | 1.44 | 2.73 | 1.12 | -1.3 | 1.95E-01 |
| 3168 | Mutilation | 1.54 | 1.04 | 1.94 | 1.28 | -1.86 | 6.49E-02 |
| 3170 | BabyTumor | 1.52 | 0.87 | 1.77 | 1.31 | -1.24 | 2.18E-01 |
| 3180 | BatteredFem | 2.88 | 0.98 | 2.27 | 1.33 | 2.86 | 5.07E-03 |
| 3181 | BatteredFem | 2.3 | 0.99 | 2.79 | 1.54 | -2.09 | 3.88E-02 |
| 3190 | Scar | 4.07 | 1.79 | 4.21 | 1.79 | -0.42 | 6.76E-01 |
| 3210 | Surgery | 4.33 | 1.51 | 4.83 | 1.8 | -1.63 | 1.06E-01 |
| 3220 | Hospital | 2.75 | 1.79 | 2.59 | 1.28 | 0.54 | 5.92E-01 |
| 3230 | DyingMan | 2.9 | 1.51 | 2.44 | 1.5 | 1.63 | 1.05E-01 |
| **3250** | **OpenChest** | **2.61** | **1.33** | **3.92** | **1.96** | **-4.3** | **3.54E-05^*^** |
| 3261 | Tumor | 1.84 | 1.42 | 1.98 | 1.19 | -0.56 | 5.73E-01 |
| 3266 | Injury | 1.97 | 1.2 | 1.98 | 1.28 | -0.04 | 9.66E-01 |
| 3280 | DentalExam | 3.87 | 1.54 | 3.83 | 1.15 | 0.15 | 8.78E-01 |
| 3300 | DisabledChild | 3.46 | 1.51 | 3.14 | 1.72 | 1.07 | 2.88E-01 |
| 3301 | InjuredChild | 2.77 | 1.02 | 2.33 | 1.69 | 1.75 | 8.31E-02 |
| 3350 | Infant | 2.84 | 1.29 | 2 | 1.62 | 3.12 | 2.27E-03 |
| **3400** | **SeveredHand** | **1.42** | **0.59** | **2.67** | **2.01** | **-4.83** | **4.33E-06^*^** |
| 3500 | Attack | 3.41 | 1.2 | 2.5 | 1.24 | 4 | 1.12E-04 |
| 3530 | Attack | 2.54 | 1.09 | 2.1 | 1.53 | 1.82 | 7.18E-02 |
| 3550 | Injury | 2.05 | 1.33 | 3.1 | 1.76 | -3.68 | 3.60E-04 |
| 3550.1 | PlaneCrash | 2.49 | 1.23 | 2.98 | 1.24 | -2.12 | 3.58E-02 |
| **3550.2** | **Coach** | **3.77** | **1.31** | **5.28** | **1.19** | **-6.41** | **3.31E-09^*^** |
| 4000 | Artist | 4.93 | 1.35 | 5.42 | 1.73 | -1.72 | 8.79E-02 |
| **4001** | **EroticFemale** | **5.92** | **1.48** | **7.2** | **1.54** | **-4.55** | **1.35E-05^*^** |
| **4002** | **EroticFemale** | **5.76** | **1.77** | **7.69** | **1.48** | **-6.25** | **7.21E-09^*^** |
| 4003 | EroticFemale | 5.39 | 1.82 | 6.67 | 1.71 | -3.86 | 1.87E-04 |
| **4004** | **EroticFemale** | **4.67** | **1.46** | **6.28** | **1.51** | **-5.82** | **5.54E-08^*^** |
| **4005** | **EroticFemale** | **4.02** | **1.93** | **6.52** | **1.58** | **-7.48** | **1.64E-11^*^** |
| 4100 | MaleDancers | 5.3 | 2.24 | 5.39 | 1.26 | -0.26 | 7.99E-01 |
| 4141 | EroticFemale | 7.08 | 1.54 | 7.46 | 1.59 | -1.3 | 1.95E-01 |
| 4142 | EroticFemale | 6.84 | 1.43 | 7.55 | 1.68 | -2.46 | 1.52E-02 |
| **4150** | **AttractiveFem** | **5.61** | **1.46** | **7.8** | **1.36** | **-8.26** | **2.76E-13^*^** |
| **4180** | **EroticFemale** | **7.03** | **1.51** | **8.21** | **1.34** | **-4.38** | **2.58E-05^*^** |
| **4210** | **EroticFemale** | **5.85** | **1.92** | **8.25** | **1.3** | **-7.63** | **7.59E-12^*^** |
| **4220** | **EroticFemale** | **6.56** | **1.06** | **8.02** | **1.37** | **-6.5** | **2.15E-09^*^** |
| 4230 | Prostitute | 5.7 | 1.83 | 6.29 | 2.29 | -1.55 | 1.24E-01 |
| **4232** | **EroticFemale** | **5.01** | **2** | **7.88** | **1.1** | **-9.16** | **2.36E-15^*^** |
| 4233 | Prostitute | 5.43 | 1.7 | 5.48 | 1.68 | -0.16 | 8.75E-01 |
| 4235 | EroticFemale | 6.62 | 1.7 | 7.29 | 1.61 | -2.16 | 3.31E-02 |
| **4240** | **EroticFemale** | **4.7** | **1.8** | **7.52** | **1.22** | **-9.56** | **2.81E-16^*^** |
| **4250** | **AttractiveFem** | **6.43** | **1.61** | **8.39** | **0.93** | **-7.7** | **5.24E-12^*^** |
| 4255 | EroticFemale | 6.43 | 1.2 | 7.34 | 1.38 | -3.8 | 2.29E-04 |
| 4274 | AttractiveFem | 6.48 | 1.29 | 6.45 | 1.57 | 0.11 | 9.10E-01 |
| **4275** | **AttractiveFem** | **6.48** | **1.43** | **7.51** | **1.08** | **-4.26** | **4.13E-05^*^** |
| **4279** | **EroticFemale** | **4.9** | **1.82** | **7.23** | **1.25** | **-7.78** | **3.39E-12^*^** |
| **4290** | **EroticFemale** | **5.17** | **2.01** | **7.61** | **1.69** | **-6.94** | **2.42E-10^*^** |
| 4300 | EroticFemale | 7.31 | 1.98 | 7.56 | 1.29 | -0.78 | 4.38E-01 |
| **4302** | **EroticFemale** | **4.75** | **2.25** | **6.84** | **2.1** | **-5.11** | **1.28E-06^*^** |
| 4310 | EroticFemale | 7.13 | 1.61 | 7.56 | 1.53 | -1.46 | 1.47E-01 |
| 4320 | EroticFemale | 6.35 | 1.61 | 7.48 | 1.92 | -3.46 | 7.65E-04 |
| 4460 | EroticMale | 5.05 | 1.82 | 4.77 | 1.23 | 0.94 | 3.50E-01 |
| 4470 | EroticMale | 4.48 | 1.41 | 4.79 | 1.16 | -1.27 | 2.08E-01 |
| 4490 | EroticMale | 3.67 | 1.42 | 4.29 | 1.31 | -2.41 | 1.73E-02 |
| 4500 | AttractiveMan | 5 | 1.69 | 4.53 | 1.4 | 1.6 | 1.13E-01 |
| 4503 | EroticMale | 5.97 | 1.53 | 5.13 | 1.04 | 3.35 | 1.11E-03 |
| **4510** | **AttractiveMan** | **5.33** | **1.15** | **4.05** | **1.72** | **4.82** | **4.43E-06^*^** |
| 4520 | EroticMale | 4.97 | 1.35 | 5.21 | 1.21 | -0.99 | 3.22E-01 |
| 4530 | EroticMale | 5 | 1.35 | 4.46 | 1.12 | 2.3 | 2.33E-02 |
| 4531 | EroticMale | 4.84 | 1.36 | 4.63 | 1.58 | 0.77 | 4.42E-01 |
| 4532 | AttractiveMan | 5.89 | 1.16 | 5.15 | 1.36 | 3.17 | 1.95E-03 |
| 4533 | AttractiveMan | 5.89 | 1.28 | 5.42 | 1.92 | 1.59 | 1.15E-01 |
| **4534** | **MaleDancers** | **7.15** | **1.68** | **4.72** | **1.27** | **8.56** | **5.73E-14^*^** |
| 4535 | Weightlifter | 6.23 | 1.25 | 5.36 | 1.35 | 3.6 | 4.73E-04 |
| 4536 | AttractiveMan | 5.56 | 1.37 | 5.24 | 1.08 | 1.36 | 1.75E-01 |
| 4537 | AttractiveMan | 4.72 | 1.28 | 4.78 | 1.3 | -0.25 | 8.04E-01 |
| 4538 | EroticMale | 5.02 | 1.1 | 4.59 | 1.46 | 1.82 | 7.17E-02 |
| 4550 | EroticMale | 4.15 | 1.89 | 3.26 | 1.69 | 2.63 | 9.58E-03 |
| **4561** | **EroticMale** | **5.39** | **1.19** | **3.82** | **1.96** | **5.37** | **4.20E-07^*^** |
| **4571** | **AttractiveMan** | **6.05** | **1.3** | **4.73** | **1.28** | **5.47** | **2.67E-07^*^** |
| **4572** | **AttractiveMan** | **5.39** | **1.1** | **4.3** | **1.2** | **5.1** | **1.35E-06^*^** |
| 4598 | Couple | 6.1 | 1.83 | 6.55 | 2.45 | -1.14 | 2.58E-01 |
| 4599 | Romance | 6.7 | 1.04 | 7.02 | 1.28 | -1.49 | 1.39E-01 |
| 4601 | Romance | 7.27 | 1.33 | 6.96 | 1.1 | 1.34 | 1.83E-01 |
| 4603 | Romance | 6.28 | 1.52 | 6.51 | 1.46 | -0.82 | 4.12E-01 |
| 4605 | Couple | 5.67 | 1.11 | 5.85 | 1.77 | -0.67 | 5.02E-01 |
| 4606 | Romance | 5.51 | 1.56 | 6.37 | 1.36 | -3.11 | 2.33E-03 |
| **4607** | **EroticCouple** | **6.72** | **1.14** | **7.99** | **1.09** | **-6.07** | **1.66E-08^*^** |
| **4608** | **EroticCouple** | **6.49** | **1.36** | **7.55** | **1.28** | **-4.28** | **3.95E-05^*^** |
| 4609 | Couple | 6.56 | 1.37 | 6.39 | 1.65 | 0.61 | 5.44E-01 |
| 4610 | Romance | 6.13 | 1.13 | 6.71 | 1.85 | -2.1 | 3.82E-02 |
| 4611 | EroticCouple | 6.52 | 1.27 | 7.27 | 1.25 | -3.18 | 1.89E-03 |
| 4613 | Condom | 5.27 | 1.26 | 5.43 | 1.79 | -0.57 | 5.72E-01 |
| 4614 | Romance | 6.25 | 1.4 | 6.38 | 1.11 | -0.54 | 5.89E-01 |
| 4617 | EroticFemale | 5.77 | 1.13 | 6.42 | 1.49 | -2.68 | 8.34E-03 |
| 4621 | Harassment | 4.72 | 6.48 | 3.83 | 1.65 | 0.95 | 3.45E-01 |
| **4622** | **Romance** | **5.33** | **1.4** | **6.7** | **1.62** | **-4.89** | **3.24E-06^*^** |
| 4623 | Romance | 6.11 | 1.38 | 6.75 | 1.63 | -2.3 | 2.35E-02 |
| 4624 | Couple | 5.35 | 1.38 | 6.44 | 1.44 | -4.15 | 6.43E-05 |
| 4625 | Couple | 5.54 | 1.25 | 6.27 | 1.45 | -2.92 | 4.24E-03 |
| 4626 | Wedding | 6.41 | 1.1 | 7.36 | 1.51 | -3.94 | 1.41E-04 |
| 4631 | BikerCouple | 5.37 | 1.73 | 5.92 | 1.92 | -1.62 | 1.07E-01 |
| 4635 | Prostitute | 3.53 | 1.29 | 4.6 | 1.66 | -3.92 | 1.49E-04 |
| 4640 | Romance | 5.51 | 1.25 | 6.67 | 1.99 | -3.86 | 1.87E-04 |
| **4641** | **Romance** | **6.08** | **1.1** | **7.16** | **1.47** | **-4.55** | **1.35E-05^*^** |
| **4650** | **EroticCouple** | **5.8** | **1.28** | **6.98** | **1.51** | **-4.57** | **1.25E-05^*^** |
| 4651 | EroticCouple | 6.54 | 1.35 | 7.52 | 1.66 | -3.52 | 6.21E-04 |
| 4652 | EroticCouple | 7.48 | 1.6 | 7.92 | 1.06 | -1.69 | 9.43E-02 |
| 4653 | EroticCouple | 6.57 | 1.44 | 7.1 | 1.27 | -2.07 | 4.07E-02 |
| 4656 | EroticCouple | 6.57 | 1.52 | 7.21 | 1.86 | -2.05 | 4.30E-02 |
| 4658 | EroticCouple | 6.6 | 2.08 | 7.35 | 1.37 | -2.22 | 2.87E-02 |
| **4659** | **EroticCouple** | **6.08** | **1.76** | **7.7** | **1.64** | **-5.07** | **1.54E-06^*^** |
| **4660** | **EroticCouple** | **5.98** | **1.55** | **7.63** | **1.3** | **-6.09** | **1.50E-08^*^** |
| 4664 | EroticCouple | 7.01 | 1.56 | 7.99 | 1.25 | -3.65 | 3.94E-04 |
| 4664.1 | Erotic | 7.15 | 1.69 | 7.44 | 1.97 | -0.86 | 3.94E-01 |
| 4664.2 | Attack | 3.26 | 1.55 | 3.73 | 1.85 | -1.49 | 1.38E-01 |
| 4666 | EroticCouple | 5.87 | 1.9 | 6.87 | 1.38 | -3.15 | 2.07E-03 |
| 4669 | EroticCouple | 5.9 | 1.87 | 6.84 | 1.93 | -2.65 | 9.10E-03 |
| 4670 | EroticCouple | 7.18 | 1.3 | 7.77 | 1.05 | -2.63 | 9.68E-03 |
| 4672 | EroticCouple | 6.72 | 1.64 | 6.44 | 2.17 | 0.8 | 4.28E-01 |
| **4676** | **EroticCouple** | **5.61** | **1.49** | **7.03** | **1.54** | **-5.03** | **1.85E-06^*^** |
| 4677 | EroticCouple | 5.51 | 1.8 | 6.53 | 1.56 | -3.21 | 1.74E-03 |
| **4680** | **EroticCouple** | **5.97** | **2.39** | **7.73** | **1.61** | **-4.5** | **1.65E-05^*^** |
| 4681 | EroticCouple | 6.1 | 1.42 | 7.09 | 1.29 | -3.88 | 1.76E-04 |
| 4683 | EroticCouple | 6.52 | 1.49 | 7.45 | 1.35 | -3.48 | 7.21E-04 |
| **4687** | **EroticCouple** | **6.1** | **1.52** | **7.25** | **1.31** | **-4.29** | **3.74E-05^*^** |
| 4689 | EroticCouple | 6.2 | 1.24 | 7 | 1.35 | -3.32 | 1.19E-03 |
| 4690 | EroticCouple | 6.26 | 1.7 | 7.42 | 1.96 | -3.42 | 8.70E-04 |
| 4700 | Couple | 5.7 | 0.9 | 6.74 | 2.03 | -3.73 | 2.94E-04 |
| 4750 | NudeFemale | 7.51 | 1.64 | 6.81 | 1.48 | 2.38 | 1.90E-02 |
| 4770 | FemaleKiss | 5.47 | 1.92 | 6.67 | 1.92 | -3.34 | 1.11E-03 |
| **4800** | **EroticCouple** | **5.89** | **2.12** | **7.43** | **1.68** | **-4.24** | **4.59E-05^*^** |
| 4810 | EroticCouple | 6.16 | 1.96 | 7.2 | 1.89 | -2.88 | 4.71E-03 |
| 5000 | Flower | 5.9 | 1.14 | 6.58 | 1.77 | -2.52 | 1.30E-02 |
| 5001 | SunFlower | 6.08 | 1.07 | 6.4 | 1.47 | -1.36 | 1.75E-01 |
| 5010 | Flower | 6.05 | 0.92 | 6.75 | 1.52 | -3.09 | 2.52E-03 |
| 5020 | Flower | 5.48 | 1.26 | 6 | 1.63 | -1.95 | 5.40E-02 |
| 5030 | Flower | 5.98 | 1.01 | 5.88 | 1.67 | 0.4 | 6.89E-01 |
| 5120 | PineNeedles | 3.75 | 1.49 | 4.72 | 0.93 | -4.05 | 9.34E-05 |
| 5130 | Rocks | 3.67 | 1.38 | 4.37 | 1.17 | -2.89 | 4.56E-03 |
| 5200 | Flowers | 6.16 | 1.11 | 6.96 | 1.62 | -3.17 | 1.96E-03 |
| 5201 | Nature | 6 | 1.64 | 6.41 | 1.72 | -1.31 | 1.93E-01 |
| 5220 | Nature | 6.46 | 1.18 | 6.94 | 1.34 | -2.05 | 4.23E-02 |
| 5250 | Nature | 5.68 | 1.33 | 6.04 | 2.06 | -1.15 | 2.54E-01 |
| 5260 | Waterfall | 6.77 | 1.72 | 7.47 | 1.71 | -2.18 | 3.11E-02 |
| 5270 | Nature | 6.44 | 1.57 | 7.2 | 1.49 | -2.65 | 9.26E-03 |
| 5300 | Galaxy | 7.23 | 1.28 | 6.83 | 1.72 | 1.44 | 1.52E-01 |
| 5390 | Boat | 6.03 | 1.55 | 5.13 | 1.56 | 3.1 | 2.45E-03 |
| **5395** | **Boat** | **4.38** | **1.16** | **5.34** | **1.24** | **-4.3** | **3.60E-05^*^** |
| 5410 | Violinist | 5.95 | 1.3 | 5.78 | 1.46 | 0.66 | 5.08E-01 |
| 5450 | Liftoff | 7.25 | 1.19 | 7.02 | 1.76 | 0.84 | 4.01E-01 |
| 5455 | Cockpit | 5.23 | 0.99 | 5.89 | 1.07 | -3.45 | 7.95E-04 |
| **5460** | **Astronaut** | **5.33** | **1.3** | **7.37** | **1.44** | **-8.02** | **9.95E-13^*^** |
| 5470 | Astronaut | 6.39 | 1.35 | 7.38 | 1.82 | -3.38 | 9.93E-04 |
| 5480 | Fireworks | 6.85 | 1.21 | 7.37 | 1.8 | -1.87 | 6.44E-02 |
| 5500 | Mushroom | 4.98 | 1.17 | 5.49 | 1.67 | -1.94 | 5.45E-02 |
| 5510 | Mushroom | 5.3 | 1.1 | 5.2 | 1.52 | 0.41 | 6.80E-01 |
| 5520 | Mushroom | 4.8 | 1.01 | 5.28 | 1.74 | -1.88 | 6.33E-02 |
| 5530 | Mushroom | 4.71 | 1.13 | 5.33 | 1.64 | -2.42 | 1.71E-02 |
| **5531** | **Mushroom** | **4.03** | **1.33** | **5.24** | **1.54** | **-4.55** | **1.34E-05^*^** |
| **5532** | **Mushrooms** | **4.02** | **1.3** | **5.43** | **1.72** | **-5.05** | **1.66E-06^*^** |
| 5533 | Mushrooms | 5.31 | 1.35 | 5.12 | 1.29 | 0.77 | 4.44E-01 |
| 5534 | Mushrooms | 4.05 | 1.49 | 4.71 | 1.6 | -2.3 | 2.35E-02 |
| 5535 | Stilllife | 4.9 | 1.29 | 4.93 | 1.14 | -0.13 | 8.96E-01 |
| 5551 | Clouds | 6.18 | 1.11 | 6.79 | 1.47 | -2.56 | 1.18E-02 |
| 5593 | Sky | 6.13 | 1.19 | 6.22 | 1.65 | -0.34 | 7.32E-01 |
| 5594 | Sky | 6.26 | 1.86 | 7.2 | 1.38 | -3.01 | 3.24E-03 |
| **5600** | **Moutains** | **5.25** | **1.67** | **7.27** | **1.64** | **-6.52** | **1.93E-09^*^** |
| 5611 | Moutains | 5.74 | 1.3 | 6.74 | 1.37 | -4.02 | 1.03E-04 |
| 5621 | SkyDivers | 6.44 | 1.41 | 7.28 | 1.22 | -3.37 | 1.01E-03 |
| 5622 | Shark | 5.74 | 1.47 | 6.44 | 1.43 | -2.58 | 1.12E-02 |
| 5623 | Windsurfers | 6.52 | 1.51 | 7.12 | 1.29 | -2.26 | 2.57E-02 |
| 5626 | HangGlider | 7.14 | 1.35 | 6.81 | 1.75 | 1.15 | 2.52E-01 |
| 5628 | Moutains | 5.85 | 1.33 | 6.42 | 1.82 | -1.96 | 5.27E-02 |
| 5629 | Hiker | 6.21 | 1.43 | 6.89 | 1.59 | -2.43 | 1.68E-02 |
| 5660 | Moutains | 6.61 | 1.53 | 7.16 | 1.66 | -1.85 | 6.62E-02 |
| 5661 | Cave | 5.28 | 1.37 | 6.02 | 1.41 | -2.85 | 5.11E-03 |
| **5700** | **Moutains** | **6.23** | **1.45** | **7.7** | **1.36** | **-5.57** | **1.70E-07^*^** |
| 5711 | Field | 5.74 | 1.39 | 6.29 | 1.34 | -2.15 | 3.37E-02 |
| 5720 | Farmland | 5.69 | 1.44 | 6.02 | 1.65 | -1.15 | 2.52E-01 |
| 5731 | Flowers | 5.52 | 1.01 | 5.19 | 1.62 | 1.35 | 1.79E-01 |
| 5740 | Plant | 5.2 | 1.19 | 5.47 | 1.27 | -1.18 | 2.41E-01 |
| 5750 | Nature | 6.08 | 1.38 | 6.33 | 1.75 | -0.86 | 3.89E-01 |
| **5760** | **Nature** | **6.44** | **1.25** | **7.69** | **1.28** | **-5.3** | **5.75E-07^*^** |
| **5779** | **Courtyard** | **5.72** | **1.11** | **6.69** | **1.21** | **-4.5** | **1.64E-05^*^** |
| 5780 | Nature | 6.56 | 1.41 | 7.35 | 1.46 | -2.95 | 3.82E-03 |
| 5800 | Leaves | 5.89 | 1.29 | 6.21 | 1.83 | -1.11 | 2.70E-01 |
| 5811 | Flowers | 5.85 | 1.29 | 6.52 | 1.65 | -2.47 | 1.52E-02 |
| 5820 | Moutains | 6.33 | 1.77 | 6.95 | 1.91 | -1.81 | 7.26E-02 |
| 5830 | Sunset | 7.6 | 1.2 | 7.37 | 1.8 | 0.83 | 4.09E-01 |
| 5831 | Seagulls | 6.11 | 1.4 | 7.07 | 1.1 | -4.01 | 1.08E-04 |
| 5849 | Flowers | 5.6 | 1.31 | 6.4 | 1.4 | -3.17 | 1.93E-03 |
| 5870 | Clouds | 5.97 | 1.15 | 6.6 | 1.62 | -2.46 | 1.54E-02 |
| 5875 | Bicyclist | 5.12 | 0.85 | 5.85 | 1.12 | -4.04 | 9.80E-05 |
| 5890 | Earth | 6.16 | 1.76 | 6.6 | 1.95 | -1.28 | 2.04E-01 |
| 5891 | Clouds | 6.44 | 1.26 | 6.83 | 1.27 | -1.65 | 1.02E-01 |
| 5900 | Desert | 5.21 | 1.39 | 6.15 | 1.69 | -3.3 | 1.30E-03 |
| **5910** | **Fireworks** | **5.56** | **1.45** | **7.41** | **1.2** | **-7.34** | **3.33E-11^*^** |
| 5920 | Volcano | 5.82 | 1.28 | 5.83 | 1.71 | -0.04 | 9.71E-01 |
| 5940 | Lava | 4.31 | 1.38 | 4.79 | 1.82 | -1.62 | 1.07E-01 |
| 5950 | Lightning | 6.07 | 1.73 | 6.8 | 1.58 | -2.34 | 2.09E-02 |
| 5970 | Tornado | 4.26 | 1.71 | 4.31 | 1.64 | -0.16 | 8.74E-01 |
| 5971 | Tornado | 4.77 | 1.41 | 4.35 | 1.64 | 1.49 | 1.40E-01 |
| 5972 | Tornado | 4.1 | 1.48 | 4.11 | 1.73 | -0.03 | 9.73E-01 |
| 5982 | Sky | 6.75 | 1.55 | 7.38 | 1.66 | -2.11 | 3.70E-02 |
| 5990 | Sky | 6.07 | 2.18 | 6.54 | 1.77 | -1.25 | 2.15E-01 |
| 5991 | Sky | 6.56 | 1.36 | 6.04 | 2.28 | 1.54 | 1.27E-01 |
| 5994 | Skyline | 5.56 | 1.28 | 6.39 | 1.39 | -3.34 | 1.11E-03 |
| 6000 | Prison | 4.88 | 1.11 | 4.19 | 1.65 | 2.7 | 7.94E-03 |
| 6010 | Jail | 3.16 | 1.21 | 4.25 | 2.35 | -3.26 | 1.45E-03 |
| 6020 | ElectricChair | 3.38 | 1.39 | 4.1 | 2.15 | -2.2 | 3.01E-02 |
| 6150 | Outlet | 4.67 | 1.39 | 5.17 | 1.13 | -2.08 | 3.97E-02 |
| 6190 | AimedGun | 4.1 | 1.62 | 4.52 | 1.79 | -1.33 | 1.87E-01 |
| 6200 | AimedGun | 3.77 | 1.13 | 3.78 | 1.65 | -0.04 | 9.69E-01 |
| 6210 | AimedGun | 4.33 | 1.36 | 3.73 | 1.87 | 2.01 | 4.68E-02 |
| 6211 | Attack | 3.65 | 1.54 | 4.25 | 1.62 | -2.04 | 4.37E-02 |
| **6212** | **Soldier** | **3.84** | **1.47** | **2.59** | **1.47** | **4.55** | **1.34E-05^*^** |
| 6213 | Terrorist | 3.77 | 1.16 | 3.75 | 1.39 | 0.08 | 9.33E-01 |
| 6230 | AimedGun | 3.13 | 1.56 | 2.73 | 1.48 | 1.4 | 1.64E-01 |
| 6241 | Gun | 4.25 | 0.98 | 4.09 | 1.3 | 0.76 | 4.49E-01 |
| 6243 | AimedGun | 3.78 | 1.61 | 2.8 | 1.61 | 3.26 | 1.48E-03 |
| 6244 | AimedGun | 4.89 | 1.91 | 3.85 | 1.93 | 2.9 | 4.46E-03 |
| 6250 | AimedGun | 3.74 | 1.35 | 2.98 | 1.97 | 2.48 | 1.48E-02 |
| **6250.2** | **IceCream** | **6.05** | **1.2** | **3.38** | **1.97** | **9.07** | **3.76E-15^*^** |
| 6260 | AimedGun | 3.55 | 1.87 | 2.53 | 1.63 | 3.08 | 2.58E-03 |
| 6300 | Knife | 3 | 1.88 | 3.3 | 1.67 | -0.9 | 3.73E-01 |
| 6311 | DistressedFem | 3.18 | 1.34 | 2.82 | 1.33 | 1.44 | 1.52E-01 |
| 6312 | Abduction | 3.08 | 1.08 | 2.88 | 1.48 | 0.85 | 4.00E-01 |
| 6313 | Attack | 2.75 | 1.37 | 2.43 | 1.42 | 1.23 | 2.21E-01 |
| **6314** | **Attack** | **5.82** | **1.43** | **3.93** | **1.39** | **7.16** | **8.30E-11^*^** |
| 6315 | BeatenFem | 3.18 | 1.22 | 2.94 | 1.89 | 0.83 | 4.07E-01 |
| 6350 | Attack | 2.97 | 1.63 | 2.39 | 1.42 | 2.01 | 4.68E-02 |
| 6360 | Attack | 3.08 | 1.7 | 2.63 | 1.7 | 1.42 | 1.59E-01 |
| 6370 | Attack | 2.51 | 1.16 | 3.24 | 1.55 | -2.91 | 4.28E-03 |
| 6410 | AimedGun | 4.28 | 1.36 | 4.43 | 1.82 | -0.51 | 6.11E-01 |
| 6415 | DeadTiger | 3.26 | 1.44 | 2.81 | 1.63 | 1.58 | 1.17E-01 |
| 6510 | Attack | 3.59 | 1.88 | 2.86 | 1.76 | 2.13 | 3.49E-02 |
| **6530** | **Attack** | **5.03** | **1.95** | **2.86** | **1.94** | **5.97** | **2.73E-08^*^** |
| 6540 | Attack | 3.93 | 2.21 | 2.53 | 1.84 | 3.64 | 4.16E-04 |
| 6550 | Attack | 2.44 | 1.59 | 3.39 | 2.63 | -2.42 | 1.69E-02 |
| 6555 | Knife | 3.18 | 1.25 | 3.74 | 1.42 | -2.26 | 2.56E-02 |
| 6560 | Attack | 2.92 | 1.35 | 2.57 | 1.49 | 1.33 | 1.87E-01 |
| 6561 | Attack | 4.55 | 1.54 | 3.58 | 1.38 | 3.52 | 6.16E-04 |
| 6570 | Suicide | 3.18 | 1.91 | 2.29 | 1.84 | 2.53 | 1.27E-02 |
| 6570.1 | Suicide | 2.61 | 1.36 | 2.96 | 1.5 | -1.32 | 1.90E-01 |
| 6570.2 | BlowDry | 4.84 | 1.32 | 4.9 | 0.97 | -0.27 | 7.87E-01 |
| 6571 | CarTheft | 3.26 | 1.53 | 3.8 | 2.16 | -1.58 | 1.16E-01 |
| 6610 | Gun | 4.28 | 1.14 | 4.59 | 1.31 | -1.36 | 1.75E-01 |
| 6800 | Gun | 4.49 | 1.16 | 5.13 | 1.91 | -2.25 | 2.67E-02 |
| 6821 | Gang | 3.44 | 1.48 | 2.96 | 1.93 | 1.52 | 1.31E-01 |
| 6830 | Guns | 3.67 | 1.26 | 3.43 | 1.74 | 0.87 | 3.89E-01 |
| 6831 | Police | 2.98 | 1.41 | 2.98 | 1.39 | 0 | 1.00E+00 |
| 6834 | Police | 3.82 | 1.15 | 3.69 | 1.77 | 0.48 | 6.32E-01 |
| 6836 | Police | 3.66 | 1.14 | 4.15 | 1.62 | -1.92 | 5.73E-02 |
| **6838** | **Police** | **4.25** | **1.22** | **2.88** | **1.51** | **5.43** | **3.25E-07^*^** |
| 6840 | Police | 3.51 | 1.27 | 4.43 | 2.04 | -3 | 3.35E-03 |
| 6900 | Aircraft | 6.08 | 1.19 | 5.15 | 1.97 | 3.17 | 1.96E-03 |
| 6910 | Bomber | 6.08 | 1.54 | 6.13 | 2.22 | -0.14 | 8.86E-01 |
| **6930** | **Missiles** | **5.77** | **1.09** | **4.46** | **1.83** | **4.83** | **4.30E-06^*^** |
| 6940 | Tank | 5.07 | 1.51 | 4.77 | 1.92 | 0.95 | 3.46E-01 |
| 7000 | RollingPin | 4.67 | 0.9 | 4.93 | 0.35 | -1.93 | 5.55E-02 |
| 7002 | Towel | 4.97 | 1.01 | 4.91 | 0.97 | 0.32 | 7.47E-01 |
| 7004 | Spoon | 5.23 | 1.19 | 4.89 | 0.69 | 1.81 | 7.36E-02 |
| 7006 | Bowl | 4.92 | 1.1 | 4.65 | 1.1 | 1.31 | 1.92E-01 |
| 7009 | Mug | 5.05 | 0.74 | 4.96 | 1.05 | 0.54 | 5.88E-01 |
| 7010 | Basket | 4.84 | 1.11 | 4.95 | 1.43 | -0.47 | 6.40E-01 |
| 7020 | Fan | 4.89 | 0.93 | 5.02 | 1.22 | -0.65 | 5.14E-01 |
| 7025 | Stool | 4.7 | 1.31 | 4.46 | 1.23 | 1.01 | 3.17E-01 |
| 7030 | Iron | 4.39 | 1 | 4.82 | 0.99 | -2.31 | 2.26E-02 |
| 7031 | Shoes | 4.1 | 1.76 | 4.2 | 1.3 | -0.34 | 7.36E-01 |
| 7034 | Hammer | 4.41 | 1.17 | 5 | 1.1 | -2.77 | 6.58E-03 |
| 7035 | Mug | 4.9 | 0.98 | 4.81 | 1.05 | 0.48 | 6.34E-01 |
| 7036 | Shipyard | 5.02 | 0.85 | 5.08 | 1.02 | -0.35 | 7.30E-01 |
| 7037 | Trains | 4.62 | 1.23 | 4.88 | 1.11 | -1.18 | 2.41E-01 |
| **7038** | **Shoes** | **4.05** | **1.24** | **4.97** | **0.93** | **-4.4** | **2.42E-05^*^** |
| 7039 | Train | 5.14 | 1.48 | 5.53 | 1.29 | -1.49 | 1.39E-01 |
| 7040 | DustPan | 4.1 | 1.16 | 4.72 | 1.19 | -2.83 | 5.52E-03 |
| 7041 | Baskets | 4.39 | 1.21 | 4.96 | 1.14 | -2.58 | 1.10E-02 |
| 7050 | HairDryer | 4.85 | 0.68 | 4.81 | 0.71 | 0.31 | 7.58E-01 |
| 7060 | TrashCan | 3.87 | 1.78 | 4.59 | 0.86 | -2.64 | 9.50E-03 |
| 7080 | Fork | 4.66 | 1.12 | 5.43 | 1.26 | -3.49 | 6.93E-04 |
| 7090 | Book | 4.78 | 0.85 | 4.95 | 1.94 | -0.64 | 5.23E-01 |
| 7095 | Headlight | 4.9 | 1.11 | 5.79 | 1.32 | -3.96 | 1.32E-04 |
| 7096 | Car | 5.11 | 1.2 | 5.7 | 1.2 | -2.63 | 9.68E-03 |
| 7100 | FireHydrant | 4.85 | 1.15 | 5.29 | 0.92 | -2.23 | 2.80E-02 |
| 7110 | Hammer | 4.08 | 1.13 | 4.51 | 1.02 | -2.12 | 3.60E-02 |
| 7130 | Truck | 4.62 | 0.9 | 4.79 | 1.14 | -0.9 | 3.69E-01 |
| 7140 | Bus | 4.97 | 0.71 | 5.59 | 1.34 | -3.23 | 1.61E-03 |
| 7150 | Umbrella | 4.93 | 1.17 | 4.76 | 0.73 | 0.9 | 3.68E-01 |
| 7160 | Fabric | 4.33 | 1.5 | 4.98 | 0.97 | -2.67 | 8.59E-03 |
| 7161 | Pole | 4.9 | 0.8 | 4.99 | 0.86 | -0.58 | 5.61E-01 |
| 7170 | LightBulb | 5.34 | 1.18 | 4.9 | 0.94 | 2.17 | 3.20E-02 |
| 7175 | Lamp | 5.16 | 1.13 | 4.78 | 1.18 | 1.77 | 8.01E-02 |
| 7179 | Rug | 5.07 | 1.06 | 5.11 | 0.95 | -0.21 | 8.33E-01 |
| 7180 | NeonBuilding | 4.79 | 1.67 | 4.76 | 1.43 | 0.1 | 9.19E-01 |
| 7182 | Checkerboard | 5.23 | 1.36 | 5.32 | 1.22 | -0.37 | 7.12E-01 |
| 7183 | Checkerboard | 5.18 | 1.35 | 5.64 | 1.56 | -1.71 | 9.08E-02 |
| 7184 | AbstractArt | 3.82 | 1.45 | 4.94 | 1.93 | -3.59 | 4.95E-04 |
| 7185 | AbstractArt | 4.92 | 1.05 | 4.84 | 1.07 | 0.4 | 6.87E-01 |
| 7186 | AbstractArt | 5.3 | 1.19 | 4.98 | 1.36 | 1.35 | 1.79E-01 |
| 7187 | AbstractArt | 4.93 | 1.38 | 4.87 | 1.12 | 0.25 | 8.02E-01 |
| 7190 | Clock | 5.92 | 1.29 | 5.5 | 1.44 | 1.66 | 1.00E-01 |
| **7195** | **Teeth** | **4.33** | **1.41** | **6.21** | **1.41** | **-7.13** | **9.29E-11^*^** |
| **7200** | **Brownie** | **5.67** | **1.43** | **7.5** | **1.78** | **-6.16** | **1.08E-08^*^** |
| 7205 | Scarves | 4.95 | 1 | 5.35 | 1.35 | -1.84 | 6.81E-02 |
| 7207 | Beads | 5.28 | 1.11 | 5 | 1.48 | 1.17 | 2.45E-01 |
| 7211 | Clock | 4.79 | 0.78 | 4.98 | 1.57 | -0.86 | 3.92E-01 |
| 7217 | clothesRack | 4.48 | 1.14 | 4.63 | 1.15 | -0.7 | 4.84E-01 |
| 7220 | Pastry | 6.59 | 1.24 | 6.46 | 1.76 | 0.47 | 6.40E-01 |
| 7224 | FileCabinets | 4.43 | 1.14 | 4.38 | 1.49 | 0.21 | 8.37E-01 |
| 7230 | Turkey | 6.26 | 1.58 | 7.42 | 1.47 | -4.05 | 9.47E-05 |
| 7233 | Plate | 5.05 | 1.23 | 5.01 | 1.21 | 0.18 | 8.61E-01 |
| 7234 | IroningBoard | 5.11 | 1.13 | 4.36 | 1.41 | 3.19 | 1.82E-03 |
| 7235 | Chair | 4.95 | 0.97 | 4.85 | 1.13 | 0.51 | 6.08E-01 |
| 7236 | Lightbulb | 5.59 | 1.07 | 5.74 | 1.33 | -0.68 | 5.01E-01 |
| 7237 | AbstractArt | 4.56 | 1.07 | 5.4 | 1.3 | -3.83 | 2.09E-04 |
| 7238 | AbstractArt | 6.12 | 1.34 | 6.26 | 1.74 | -0.49 | 6.24E-01 |
| 7250 | Cake | 6.02 | 1.36 | 6.63 | 1.39 | -2.38 | 1.91E-02 |
| 7260 | Torte | 6.23 | 0.96 | 7.1 | 1.43 | -3.93 | 1.43E-04 |
| **7270** | **IceCream** | **5.49** | **1.62** | **7.24** | **1.76** | **-5.57** | **1.69E-07^*^** |
| **7280** | **Wines** | **5.31** | **1.35** | **6.59** | **1.95** | **-4.19** | **5.40E-05^*^** |
| 7281 | Food | 6.46 | 1.3 | 6.13 | 1.25 | 1.38 | 1.70E-01 |
| 7282 | Cake | 6.93 | 1.12 | 6.31 | 1.45 | 2.61 | 1.03E-02 |
| 7283 | Fruit | 6.16 | 1.4 | 5.2 | 1.6 | 3.45 | 7.82E-04 |
| 7284 | Fruit | 5.62 | 1.69 | 5.93 | 1.63 | -1 | 3.21E-01 |
| 7285 | Tomatoes | 6.11 | 1.38 | 5.3 | 1.23 | 3.29 | 1.34E-03 |
| 7286 | Pancakes | 6.21 | 1.21 | 6.3 | 1.79 | -0.32 | 7.46E-01 |
| 7289 | Food | 6.18 | 1.15 | 6.58 | 1.98 | -1.37 | 1.72E-01 |
| 7291 | Chicken | 6.31 | 1.64 | 6.35 | 1.69 | -0.13 | 8.98E-01 |
| 7320 | Desserts | 5.5 | 1.71 | 6.37 | 1.55 | -2.83 | 5.46E-03 |
| 7325 | Watermelon | 6.7 | 1.42 | 6.48 | 1.47 | 0.82 | 4.16E-01 |
| 7330 | IceCream | 6.49 | 1.71 | 7.29 | 2.21 | -2.21 | 2.92E-02 |
| **7340** | **IceCream** | **5.21** | **1.43** | **6.4** | **1.61** | **-4.22** | **4.92E-05^*^** |
| 7350 | Pizza | 6.56 | 1.04 | 7.08 | 2.12 | -1.75 | 8.32E-02 |
| 7351 | Pizza | 5.82 | 1.34 | 5.57 | 1.73 | 0.88 | 3.80E-01 |
| 7352 | Pizza | 5.78 | 1.5 | 6.27 | 2.22 | -1.42 | 1.57E-01 |
| **7359** | **PieW/bug** | **5.43** | **1.86** | **3.38** | **1.75** | **6.05** | **1.88E-08^*^** |
| **7360** | **FliesOnPie** | **6.38** | **1.33** | **4.29** | **1.99** | **6.8** | **4.87E-10^*^** |
| **7361** | **MeatSlicer** | **1.86** | **1.32** | **3.65** | **1.56** | **-6.71** | **7.66E-10^*^** |
| 7380 | RoachOnPizza | 3.56 | 1.81 | 2.61 | 1.55 | 2.98 | 3.49E-03 |
| 7390 | IceCream | 5.89 | 1.14 | 6.69 | 1.56 | -3.21 | 1.74E-03 |
| **7400** | **Candy** | **5.52** | **1.44** | **6.67** | **1.49** | **-4.21** | **5.08E-05^*^** |
| 7402 | Pastry | 6.61 | 1.19 | 6.19 | 1.54 | 1.66 | 9.88E-02 |
| 7410 | Candy | 5.7 | 1.37 | 6.8 | 1.49 | -4.14 | 6.70E-05 |
| 7430 | Candy | 6.23 | 1.36 | 6.88 | 1.84 | -2.2 | 3.00E-02 |
| 7450 | Cheeseburger | 6.54 | 1.23 | 6.52 | 1.87 | 0.07 | 9.45E-01 |
| 7460 | FrenchFries | 6.9 | 1.56 | 6.9 | 1.9 | 0 | 1.00E+00 |
| 7470 | Pancakes | 6.05 | 1.42 | 6.98 | 1.5 | -3.42 | 8.64E-04 |
| 7472 | Grapes | 6.21 | 1.4 | 6.38 | 1.44 | -0.64 | 5.22E-01 |
| 7475 | Shrimp | 6.41 | 1.2 | 6.04 | 1.67 | 1.39 | 1.66E-01 |
| 7480 | Pasta | 6.46 | 1.23 | 7.16 | 1.64 | -2.64 | 9.46E-03 |
| 7481 | Food | 6.48 | 1.49 | 6.45 | 1.3 | 0.11 | 9.10E-01 |
| 7490 | Window | 4.85 | 0.87 | 5.31 | 1.27 | -2.32 | 2.19E-02 |
| 7491 | Building | 4.79 | 1.07 | 4.87 | 0.94 | -0.42 | 6.74E-01 |
| 7493 | Man | 4.97 | 0.76 | 5.12 | 1.1 | -0.87 | 3.85E-01 |
| 7495 | Store | 5.07 | 1.03 | 5.83 | 1.51 | -3.23 | 1.59E-03 |
| **7496** | **Street** | **4.56** | **1.1** | **5.83** | **1.61** | **-5.07** | **1.56E-06^*^** |
| 7500 | Building | 5.66 | 1.14 | 5.44 | 1.36 | 0.95 | 3.44E-01 |
| **7501** | **City** | **5.25** | **1.09** | **6.9** | **1.61** | **-6.61** | **1.28E-09^*^** |
| 7502 | Castle | 6.34 | 1.68 | 7.3 | 1.44 | -3.25 | 1.53E-03 |
| 7503 | CardDealer | 5.33 | 1.21 | 5.96 | 1.61 | -2.42 | 1.72E-02 |
| 7504 | Stairs | 4.9 | 1.03 | 5.69 | 1.38 | -3.55 | 5.65E-04 |
| 7510 | Skyscraper | 5.62 | 1.17 | 6.12 | 1.68 | -1.9 | 6.03E-02 |
| 7545 | Ocean | 5.57 | 1.15 | 6.52 | 1.71 | -3.59 | 4.87E-04 |
| 7550 | Office | 4.97 | 1.02 | 5.39 | 1.24 | -2.01 | 4.70E-02 |
| 7560 | Freeway | 5.05 | 0.88 | 4.48 | 1.7 | 2.36 | 2.02E-02 |
| **7570** | **Skyline** | **4.78** | **1.83** | **6.6** | **1.87** | **-5.27** | **6.39E-07^*^** |
| 7580 | Desert | 7.15 | 1.39 | 7.4 | 1.77 | -0.86 | 3.94E-01 |
| 7590 | Traffic | 5.02 | 1.07 | 4.17 | 1.4 | 3.72 | 3.06E-04 |
| 7595 | Traffic | 4.82 | 0.85 | 4.6 | 1.69 | 0.92 | 3.59E-01 |
| 7600 | Dragon | 5.28 | 1.62 | 6.19 | 1.26 | -3.3 | 1.30E-03 |
| 7620 | Jet | 5.26 | 0.81 | 5.81 | 1.67 | -2.35 | 2.03E-02 |
| 7640 | Skyscraper | 4.98 | 1.22 | 5.31 | 1.17 | -1.47 | 1.44E-01 |
| 7700 | Office | 4.08 | 1.2 | 4.31 | 1.55 | -0.9 | 3.67E-01 |
| 7705 | Cabinet | 4.8 | 1.3 | 4.47 | 0.79 | 1.59 | 1.15E-01 |
| 7710 | Bed | 4.68 | 0.84 | 5.33 | 1.53 | -2.94 | 4.00E-03 |
| 7820 | Agate | 5.18 | 1.06 | 5.65 | 1.45 | -2.03 | 4.51E-02 |
| **7830** | **Agate** | **4.92** | **1.63** | **7.29** | **1.66** | **-7.72** | **4.69E-12^*^** |
| **7900** | **Violin** | **5.1** | **0.97** | **6.5** | **1.79** | **-5.43** | **3.22E-07^*^** |
| 7920 | CarCrash | 3.66 | 1.14 | 4.67 | 1.53 | -4.09 | 7.93E-05 |
| 7950 | Tissue | 5.23 | 1.1 | 4.62 | 1.26 | 2.79 | 6.21E-03 |
| 8010 | Runner | 4.84 | 0.86 | 4.58 | 1.87 | 1.01 | 3.17E-01 |
| 8021 | Skier | 6.2 | 1 | 6.69 | 1.57 | -2.06 | 4.19E-02 |
| **8030** | **Skier** | **5.37** | **0.97** | **7.29** | **1.66** | **-7.85** | **2.42E-12^*^** |
| 8031 | Skier | 6.64 | 0.97 | 6.77 | 1.2 | -0.65 | 5.18E-01 |
| **8032** | **IceSkater** | **6.64** | **1.39** | **5.52** | **1.31** | **4.42** | **2.27E-05^*^** |
| 8033 | IceSkater | 5.9 | 1.15 | 5.82 | 1.25 | 0.36 | 7.21E-01 |
| **8034** | **Skier** | **5.66** | **1.01** | **6.9** | **1.41** | **-5.54** | **1.91E-07^*^** |
| 8040 | Diver | 5.7 | 1.22 | 6.56 | 1.53 | -3.38 | 9.85E-04 |
| 8041 | Diver | 5.9 | 1.12 | 5.68 | 1.54 | 0.89 | 3.73E-01 |
| 8050 | Rower | 5.05 | 1.16 | 6.06 | 1.54 | -4.05 | 9.41E-05 |
| 8060 | Boxer | 5.34 | 1.98 | 6.6 | 1.92 | -3.45 | 7.87E-04 |
| **8080** | **Sailing** | **5.49** | **1.29** | **7.73** | **1.25** | **-9.41** | **5.99E-16^*^** |
| 8090 | Gymnast | 5.92 | 1.02 | 6.56 | 1.34 | -2.93 | 4.04E-03 |
| **8116** | **Football** | **4.93** | **1.11** | **7.02** | **1.8** | **-7.74** | **4.22E-12^*^** |
| **8117** | **Hockey** | **5.31** | **1.23** | **6.47** | **1.33** | **-4.87** | **3.54E-06^*^** |
| 8120 | Athlete | 6.3 | 1.2 | 7.02 | 1.33 | -3.06 | 2.71E-03 |
| 8130 | PoleVaulter | 6.13 | 1.36 | 6.29 | 1.19 | -0.66 | 5.08E-01 |
| 8160 | RockClimber | 5.82 | 1.16 | 5.54 | 1.95 | 0.97 | 3.35E-01 |
| **8161** | **HangGlider** | **5.44** | **1.03** | **6.5** | **1.55** | **-4.44** | **2.08E-05^*^** |
| 8162 | HotAirBalloon | 6.3 | 1.09 | 6.45 | 1.28 | -0.68 | 4.96E-01 |
| **8170** | **Sailboat** | **6.21** | **1.13** | **7.67** | **1.44** | **-6.14** | **1.18E-08^*^** |
| 8178 | Cliffdiver | 6.18 | 1.38 | 6.88 | 1.57 | -2.56 | 1.18E-02 |
| **8179** | **Bungee** | **5.31** | **1.27** | **6.96** | **1.58** | **-6.26** | **6.85E-09^*^** |
| **8180** | **CliffDivers** | **6** | **1.45** | **7.5** | **1.74** | **-5.08** | **1.47E-06^*^** |
| **8185** | **SkyDivers** | **5.98** | **1.2** | **7.32** | **1.58** | **-5.22** | **8.20E-07^*^** |
| **8186** | **Skysurfer** | **5.72** | **1.29** | **7.22** | **1.38** | **-6.04** | **1.95E-08^*^** |
| 8190 | Skier | 7.05 | 1.56 | 8.13 | 1.29 | -3.98 | 1.20E-04 |
| 8191 | IceClimber | 6.49 | 1.25 | 6.49 | 1.6 | 0 | 1.00E+00 |
| **8192** | **VolcanoSkier** | **4.26** | **1.46** | **5.88** | **1.4** | **-6.04** | **1.92E-08^*^** |
| 8193 | Skier | 5.97 | 1.06 | 6.53 | 1.42 | -2.44 | 1.61E-02 |
| **8200** | **WaterSkier** | **6.14** | **1.02** | **7.15** | **1.54** | **-4.26** | **4.16E-05^*^** |
| **8210** | **Boat** | **6.1** | **1.12** | **7.46** | **1.26** | **-6.16** | **1.11E-08^*^** |
| 8211 | Sailboat | 5.59 | 1.22 | 6 | 1.7 | -1.52 | 1.31E-01 |
| 8220 | Runners | 5.64 | 1.43 | 6.46 | 1.6 | -2.92 | 4.27E-03 |
| **8230** | **Boxer** | **2.26** | **1.34** | **4.17** | **1.99** | **-6.2** | **9.13E-09^*^** |
| **8231** | **Boxer** | **3.07** | **1.28** | **4.6** | **1.54** | **-5.86** | **4.47E-08^*^** |
| 8232 | Boxer | 5.11 | 1.16 | 5.85 | 1.74 | -2.76 | 6.78E-03 |
| 8250 | Motorcyclist | 5.75 | 1.21 | 6.57 | 1.68 | -3.07 | 2.67E-03 |
| 8251 | Motorcycle | 5.74 | 1.22 | 6.49 | 1.58 | -2.9 | 4.50E-03 |
| **8260** | **Motorcyclist** | **5.62** | **1.32** | **6.9** | **1.6** | **-4.74** | **6.25E-06^*^** |
| 8280 | Diver | 5.82 | 0.92 | 6.43 | 1.34 | -2.92 | 4.23E-03 |
| **8300** | **Pilot** | **5.2** | **0.85** | **7.54** | **1.38** | **-11.31** | **2.18E-20^*^** |
| 8311 | Golfer | 5.7 | 1.09 | 6.08 | 1.94 | -1.35 | 1.81E-01 |
| 8320 | CarRacer | 5.25 | 1.16 | 6.33 | 1.68 | -4.11 | 7.38E-05 |
| 8330 | Winner | 5.74 | 1.44 | 6.22 | 1.15 | -1.94 | 5.49E-02 |
| 8340 | Plane | 6.31 | 9.28 | 7.41 | 1.72 | -0.83 | 4.10E-01 |
| **8341** | **Wingwalker** | **5.52** | **1.18** | **6.73** | **1.44** | **-4.99** | **2.15E-06^*^** |
| **8350** | **TennisPlayer** | **5.59** | **1.33** | **6.8** | **1.41** | **-4.74** | **6.06E-06^*^** |
| **8370** | **Rafting** | **5.62** | **1.17** | **7.67** | **1.19** | **-9.31** | **1.07E-15^*^** |
| **8380** | **Athletes** | **5.77** | **1.09** | **7.25** | **1.79** | **-5.54** | **1.99E-07^*^** |
| **8400** | **Rafters** | **5.54** | **1.09** | **7.43** | **1.4** | **-8.21** | **3.62E-13^*^** |
| **8420** | **Tubing** | **5.56** | **1.31** | **7.61** | **1.61** | **-7.59** | **9.19E-12^*^** |
| 8460 | Runner | 5.59 | 1.2 | 5.52 | 0.98 | 0.34 | 7.37E-01 |
| 8461 | HappyTeens | 6.2 | 1.03 | 6.87 | 1.66 | -2.68 | 8.33E-03 |
| 8465 | Runner | 5.31 | 1.32 | 5.24 | 1.29 | 0.29 | 7.75E-01 |
| **8470** | **Gymnast** | **5.36** | **1.34** | **7.55** | **1.71** | **-7.77** | **3.67E-12^*^** |
| 8475 | Biking/train | 5.05 | 1.45 | 5.51 | 1.71 | -1.57 | 1.19E-01 |
| 8480 | BikerOnFire | 3.82 | 2.31 | 4.5 | 2.09 | -1.64 | 1.04E-01 |
| 8485 | Fire | 3.79 | 1.52 | 3.23 | 1.71 | 1.87 | 6.42E-02 |
| 8490 | RollerCoaster | 5.93 | 0.93 | 6.85 | 2.36 | -2.91 | 4.39E-03 |
| 8496 | WaterSlide | 7.03 | 1.12 | 7.09 | 1.32 | -0.27 | 7.91E-01 |
| **8497** | **CarnivalRide** | **5.02** | **1.7** | **6.76** | **1.51** | **-5.74** | **7.79E-08^*^** |
| 8500 | Gold | 7.1 | 1.25 | 6.71 | 1.74 | 1.41 | 1.61E-01 |
| 8501 | Money | 7.32 | 1.5 | 8.14 | 1.24 | -3.15 | 2.11E-03 |
| 8502 | Money | 6.98 | 1.7 | 7.33 | 1.63 | -1.12 | 2.65E-01 |
| 8503 | Money | 5.82 | 1.55 | 6.93 | 1.81 | -3.57 | 5.30E-04 |
| 8510 | SportCar | 7.66 | 10.46 | 7.62 | 1.54 | 0.03 | 9.79E-01 |
| 8531 | SportCar | 6.47 | 1.73 | 6.94 | 1.35 | -1.59 | 1.14E-01 |
| 8540 | Athletes | 6.13 | 1.43 | 7.28 | 1.59 | -4.1 | 7.69E-05 |
| **8600** | **Mascot** | **5.08** | **1.35** | **6.25** | **1.65** | **-4.22** | **4.99E-05^*^** |
| 8620 | Woman | 6.29 | 1.15 | 6.11 | 1.24 | 0.81 | 4.20E-01 |
| **9000** | **Cemetery** | **4.54** | **1.44** | **2.81** | **1.65** | **6.04** | **1.95E-08^*^** |
| 9001 | Cemetery | 4.57 | 1.69 | 3.41 | 2.15 | 3.27 | 1.43E-03 |
| 9005 | HIVTattoo | 4.97 | 1.98 | 4.15 | 2.31 | 2.06 | 4.14E-02 |
| **9006** | **HIVTattoo** | **4.31** | **1.28** | **2.63** | **1.63** | **6.24** | **7.36E-09^*^** |
| 9007 | Needles | 2.71 | 1.3 | 2.67 | 1.29 | 0.17 | 8.69E-01 |
| **9008** | **Needle** | **2.26** | **1.25** | **3.87** | **1.93** | **-5.47** | **2.71E-07^*^** |
| 9010 | BarbedWire | 4 | 1.28 | 3.68 | 1.57 | 1.21 | 2.27E-01 |
| 9040 | StarvingChild | 2.02 | 1.22 | 1.88 | 1.17 | 0.62 | 5.33E-01 |
| 9041 | ScareChild | 3.72 | 1.03 | 3.43 | 1.49 | 1.24 | 2.16E-01 |
| **9042** | **StickThruLip** | **2.32** | **1.46** | **3.93** | **1.98** | **-5.06** | **1.58E-06^*^** |
| 9045 | NativeFem | 3.05 | 1.59 | 3.96 | 1.13 | -3.45 | 7.91E-04 |
| 9046 | Family | 3.93 | 1.24 | 3.87 | 1.19 | 0.26 | 7.93E-01 |
| 9050 | PlaneCrash | 3.31 | 1.27 | 3.05 | 1.7 | 0.95 | 3.45E-01 |
| 9070 | Boy | 4.7 | 0.99 | 4.88 | 1.94 | -0.65 | 5.14E-01 |
| 9080 | Wires | 3.59 | 1.5 | 4.05 | 1.6 | -1.59 | 1.13E-01 |
| **9090** | **Exhaust** | **4.77** | **1.75** | **3.24** | **1.46** | **5.01** | **1.95E-06^*^** |
| 9101 | Cocaine | 4.21 | 1.38 | 3.91 | 2.08 | 0.94 | 3.51E-01 |
| 9102 | Heroin | 3.38 | 1.65 | 3.74 | 1.73 | -1.14 | 2.55E-01 |
| 9110 | Puddle | 3.69 | 0.96 | 3.78 | 1.41 | -0.41 | 6.82E-01 |
| 9120 | OilFires | 3.59 | 1.49 | 3.51 | 1.97 | 0.25 | 8.03E-01 |
| 9140 | Cow | 2.39 | 1.17 | 2.56 | 1.42 | -0.71 | 4.80E-01 |
| **9156** | **Plane** | **5.6** | **1.32** | **6.81** | **1.66** | **-4.39** | **2.52E-05^*^** |
| 9160 | Soldier | 3.93 | 1.21 | 3.71 | 1.49 | 0.88 | 3.80E-01 |
| 9171 | Fisher | 3.89 | 1.43 | 4.66 | 1.95 | -2.47 | 1.52E-02 |
| 9180 | Seal | 3.66 | 1.4 | 2.76 | 1.36 | 3.48 | 7.05E-04 |
| 9181 | DeadCows | 2.82 | 1.19 | 2.54 | 1.69 | 1.05 | 2.95E-01 |
| 9182 | Horses | 2.9 | 1.39 | 3.39 | 1.76 | -1.68 | 9.52E-02 |
| 9190 | Woman | 3.57 | 1.27 | 4.22 | 1.19 | -2.81 | 5.78E-03 |
| 9210 | Rain | 4.33 | 1.22 | 4.41 | 1.85 | -0.28 | 7.79E-01 |
| **9220** | **Cemetery** | **4.26** | **1.39** | **2.27** | **1.61** | **7.16** | **8.27E-11^*^** |
| 9230 | OilFire | 3.75 | 1.42 | 4.24 | 1.57 | -1.76 | 8.03E-02 |
| 9250 | WarVictim | 3.08 | 1.19 | 2.85 | 1.47 | 0.93 | 3.52E-01 |
| 9252 | DeadBody | 3.15 | 1.72 | 2.51 | 1.78 | 1.96 | 5.22E-02 |
| 9253 | Mutilation | 2.43 | 1.38 | 2.51 | 1.23 | -0.32 | 7.46E-01 |
| 9265 | HungMan | 2.67 | 1.3 | 2.85 | 1.44 | -0.71 | 4.81E-01 |
| 9270 | ToxicWaste | 4.03 | 1.23 | 3.39 | 1.5 | 2.53 | 1.26E-02 |
| 9280 | Smoke | 3.27 | 1.23 | 2.96 | 1.63 | 1.17 | 2.43E-01 |
| 9290 | Garbage | 3.31 | 1.22 | 3.06 | 1.63 | 0.95 | 3.45E-01 |
| 9300 | Dirty | 2.38 | 1.62 | 2.9 | 2.07 | -1.52 | 1.30E-01 |
| 9301 | Toilet | 2.16 | 1.36 | 2.72 | 1.72 | -1.97 | 5.17E-02 |
| 9320 | Vomit | 2.2 | 1.25 | 3.07 | 1.96 | -2.92 | 4.15E-03 |
| 9330 | Garbage | 3.39 | 1.39 | 3 | 1.76 | 1.34 | 1.83E-01 |
| **9331** | **HomelessMan** | **4.23** | **0.97** | **3.09** | **1.27** | **5.51** | **2.26E-07^*^** |
| 9340 | Garbage | 3.33 | 1.26 | 2.39 | 1.48 | 3.7 | 3.28E-04 |
| 9341 | Pollution | 3.26 | 1.24 | 3.52 | 1.49 | -1.03 | 3.06E-01 |
| 9342 | Pollution | 3.26 | 1.14 | 3.22 | 1.45 | 0.17 | 8.68E-01 |
| 9360 | EmptyPool | 4.51 | 1.3 | 3.96 | 1.43 | 2.17 | 3.21E-02 |
| 9373 | Garbage | 2.95 | 1.07 | 3.85 | 1.29 | -4.12 | 7.16E-05 |
| **9390** | **Dishes** | **4.36** | **1.17** | **3** | **1.76** | **5.02** | **1.94E-06^*^** |
| 9400 | Soldier | 2.84 | 1.24 | 3.19 | 1.63 | -1.32 | 1.90E-01 |
| 9401 | Knives | 4.39 | 1.35 | 4.85 | 1.05 | -2 | 4.79E-02 |
| 9402 | Mob | 3.69 | 1.52 | 4.27 | 2.06 | -1.75 | 8.23E-02 |
| 9404 | Soldiers | 4.28 | 1.37 | 4.5 | 1.53 | -0.82 | 4.15E-01 |
| 9405 | SlicedHand | 2.2 | 0.93 | 2.09 | 1.27 | 0.54 | 5.90E-01 |
| 9409 | MenW/guns | 3.75 | 1.26 | 4.13 | 1.75 | -1.37 | 1.75E-01 |
| 9410 | Soldier | 2.52 | 1.18 | 1.96 | 1.56 | 2.21 | 2.90E-02 |
| 9411 | Boy | 5.21 | 1.39 | 4.99 | 1.42 | 0.84 | 4.03E-01 |
| 9415 | Handicapped | 3.54 | 1.25 | 3.06 | 1.83 | 1.68 | 9.47E-02 |
| **9417** | **Ticket** | **4.43** | **1.04** | **3.4** | **1.49** | **4.4** | **2.40E-05^*^** |
| 9420 | Soldier | 2.21 | 1.14 | 2.96 | 1.44 | -3.14 | 2.12E-03 |
| **9421** | **Soldier** | **4.13** | **1.42** | **2.47** | **1.71** | **5.73** | **8.20E-08^*^** |
| 9430 | Burial | 3.59 | 1.26 | 3.1 | 1.51 | 1.91 | 5.85E-02 |
| 9432 | Mastactomy | 4.02 | 1.67 | 3.29 | 1.74 | 2.3 | 2.34E-02 |
| 9433 | DeadMan | 2.59 | 1.05 | 2.39 | 1.38 | 0.89 | 3.75E-01 |
| 9435 | Accident | 3.48 | 1.18 | 2.78 | 1.5 | 2.83 | 5.57E-03 |
| **9440** | **Skulls** | **2.75** | **1.41** | **4.42** | **1.82** | **-5.59** | **1.53E-07^*^** |
| 9452 | Gun | 3.28 | 1.6 | 3.62 | 2.02 | -1.02 | 3.12E-01 |
| 9470 | Ruins | 3.74 | 1.47 | 3.62 | 1.18 | 0.47 | 6.36E-01 |
| 9471 | BurntBldg | 3.87 | 1.26 | 3.43 | 1.3 | 1.84 | 6.78E-02 |
| 9472 | Bridge | 4.15 | 1.24 | 4.28 | 2.23 | -0.4 | 6.89E-01 |
| **9480** | **Skull** | **2.95** | **1.2** | **4.06** | **1.37** | **-4.66** | **8.65E-06^*^** |
| **9490** | **Corpse** | **2.87** | **1.32** | **4.2** | **1.53** | **-5.04** | **1.78E-06^*^** |
| 9495 | Fire | 3.39 | 1.13 | 4.06 | 1.7 | -2.56 | 1.18E-02 |
| 9500 | Porpoises | 3.21 | 1.47 | 2.85 | 1.86 | 1.17 | 2.45E-01 |
| **9520** | **Kids** | **4.67** | **1.33** | **2.14** | **1.44** | **9.82** | **6.59E-17^*^** |
| 9530 | Boys | 3.03 | 1.12 | 3.65 | 1.92 | -2.19 | 3.04E-02 |
| **9560** | **DuckInOil** | **3.97** | **1.52** | **2.07** | **1.89** | **6.02** | **2.09E-08^*^** |
| **9561** | **SickKitty** | **4.82** | **1.88** | **3.2** | **2.07** | **4.42** | **2.29E-05^*^** |
| 9570 | Dog | 2.39 | 1.43 | 1.9 | 1.4 | 1.85 | 6.69E-02 |
| 9571 | Cat | 3.49 | 4.11 | 2.65 | 1.62 | 1.37 | 1.74E-01 |
| 9582 | DentalExam | 3.9 | 1.26 | 4.09 | 1.63 | -0.71 | 4.78E-01 |
| 9584 | DentalExam | 3.79 | 1.02 | 3.44 | 1.62 | 1.43 | 1.55E-01 |
| 9592 | Injection | 4.21 | 1.17 | 4 | 1.66 | 0.8 | 4.24E-01 |
| 9594 | Injection | 3.9 | 1.14 | 4.08 | 1.25 | -0.81 | 4.19E-01 |
| 9600 | Ship | 3.43 | 1.4 | 3.11 | 1.8 | 1.08 | 2.82E-01 |
| 9611 | PlaneCrash | 3.98 | 1.33 | 3.02 | 1.95 | 3.16 | 1.99E-03 |
| **9620** | **Shipwreck** | **4.54** | **1.57** | **3.27** | **1.61** | **4.28** | **3.86E-05^*^** |
| **9621** | **Ship** | **5.13** | **1.4** | **3.54** | **1.85** | **5.29** | **5.82E-07^*^** |
| 9622 | Jet | 4.18 | 1.82 | 3.97 | 1.87 | 0.61 | 5.43E-01 |
| **9630** | **Bomb** | **5.89** | **1.44** | **2.96** | **1.94** | **9.38** | **7.21E-16^*^** |
| 9635.1 | ManOnFire | 3.39 | 1.59 | 2.45 | 1.5 | 3.24 | 1.56E-03 |
| 9700 | Trash | 4.61 | 1.14 | 5 | 1.08 | -1.87 | 6.38E-02 |
| **9800** | **Skinhead** | **4.33** | **1.42** | **2.48** | **1.85** | **6.12** | **1.33E-08^*^** |
| **9810** | **KKKrally** | **4.03** | **1.56** | **2.25** | **1.84** | **5.65** | **1.17E-07^*^** |
| 9830 | Cigarettes | 3.1 | 1.04 | 2.65 | 1.63 | 1.82 | 7.16E-02 |
| 9910 | CarAccident | 3.34 | 1.4 | 2.35 | 1.34 | 3.85 | 1.92E-04 |
| 9911 | CarAccident | 3.54 | 1.13 | 2.81 | 1.42 | 3.1 | 2.47E-03 |
| 9912 | Firefigher | 3.23 | 1.28 | 3.4 | 1.61 | -0.64 | 5.26E-01 |
| 9913 | Truck | 4 | 1.28 | 4.85 | 1.72 | -3.07 | 2.71E-03 |
| 9920 | CarAccident | 3.1 | 0.94 | 2.63 | 1.42 | 2.15 | 3.35E-02 |
| 9921 | Fire | 3.61 | 1.45 | 2.6 | 1.68 | 3.48 | 7.05E-04 |

^#^ The data about American participants were collected by Lang.

*P<6×10^-5^
