# Supplementary material for: Affective reactions differ between Chinese and American healthy young adults: a cross-cultural study using the international affective picture system
Source: BMC Psychiatry. 2015 Mar 27;15:60. doi: 10.1186/s12888-015-0442-9 (PMC4378560; doi:10.1186/s12888-015-0442-9)
Supplement: Additional file 5: Table S4. — Arousal scores of Chinese and American male participants. [file 12888_2015_442_MOESM5_ESM.doc]

Table S4. Arousal scores of Chinese and American male participants

| No. | description | China | | America^#^ | | t | p |
| --- | --- | --- | --- | --- | --- | --- | --- |
|  |  | mean | SD | mean | SD |  |  |
| 1019 | Snake | 5.69 | 1.79 | 5.34 | 1.76 | 1.05 | 2.98E-01 |
| 1022 | Snake | 4.86 | 1.98 | 5.83 | 1.86 | -2.7 | 8.09E-03 |
| 1030 | Snake | 5.52 | 1.93 | 5.39 | 2.4 | 0.32 | 7.46E-01 |
| 1040 | Snake | 5.36 | 1.81 | 6.18 | 2.23 | -2.19 | 3.02E-02 |
| 1050 | Snake | 7.32 | 1.44 | 6.84 | 1.55 | 1.74 | 8.44E-02 |
| 1051 | Snake | 5.11 | 2.02 | 5.79 | 1.69 | -1.93 | 5.62E-02 |
| 1052 | Snake | 5.66 | 1.89 | 5.92 | 2.2 | -0.7 | 4.88E-01 |
| 1070 | Snake | 5.05 | 1.82 | 6.25 | 2.22 | -3.21 | 1.72E-03 |
| 1080 | Snake | 5.13 | 1.85 | 5.56 | 2.49 | -1.07 | 2.86E-01 |
| 1090 | Snake | 4.66 | 1.92 | 5.85 | 2.07 | -3.22 | 1.68E-03 |
| 1101 | Snake | 4.78 | 1.86 | 5.36 | 2.14 | -1.56 | 1.21E-01 |
| 1110 | Snake | 4.95 | 1.82 | 6.02 | 2.07 | -2.96 | 3.78E-03 |
| **1111** | **Snake** | **6.78** | **1.83** | **4.45** | **1.99** | **6.57** | **1.56E-09^*^** |
| 1112 | Snake | 4.08 | 2.24 | 4.4 | 2.44 | -0.74 | 4.63E-01 |
| 1113 | Snake | 6.69 | 1.81 | 5.73 | 2.07 | 2.66 | 8.93E-03 |
| 1114 | Snake | 5.31 | 2.16 | 6.33 | 2.16 | -2.52 | 1.31E-02 |
| 1120 | Snake | 6.06 | 1.53 | 6.6 | 1.38 | -1.97 | 5.14E-02 |
| 1121 | Lizard | 5.58 | 1.57 | 4.45 | 1.86 | 3.55 | 5.60E-04 |
| 1200 | Spider | 4.41 | 1.85 | 5.36 | 2.62 | -2.3 | 2.33E-02 |
| 1201 | Spider | 5.92 | 1.85 | 5.75 | 1.99 | 0.48 | 6.35E-01 |
| 1205 | Spider | 5.41 | 1.96 | 5.61 | 2.13 | -0.54 | 5.93E-01 |
| 1220 | Spider | 5.06 | 1.76 | 5.4 | 2.5 | -0.86 | 3.90E-01 |
| 1230 | Spider | 5.13 | 1.8 | 3.9 | 2.28 | 3.25 | 1.51E-03 |
| 1240 | Spider | 4.31 | 1.74 | 4.83 | 2.26 | -1.41 | 1.62E-01 |
| 1270 | Roach | 3.84 | 1.92 | 3.9 | 2.19 | -0.16 | 8.75E-01 |
| 1274 | Roaches | 4.88 | 1.98 | 5.09 | 2.14 | -0.55 | 5.85E-01 |
| 1275 | Roaches | 4.45 | 1.93 | 4.14 | 2.14 | 0.82 | 4.14E-01 |
| **1280** | **Rat** | **6.13** | **1.96** | **4.48** | **1.84** | **4.62** | **9.96E-06^*^** |
| **1300** | **PitBull** | **5.61** | **1.63** | **6.9** | **1.59** | **-4.28** | **3.90E-05^*^** |
| 1301 | Dog | 5.91 | 1.55 | 5.63 | 2.39 | 0.77 | 4.44E-01 |
| 1302 | Dog | 5.23 | 1.71 | 5.89 | 1.79 | -2.02 | 4.52E-02 |
| 1303 | Dog | 5.09 | 1.72 | 5.24 | 2.35 | -0.4 | 6.91E-01 |
| 1310 | Leopard | 6.3 | 1.79 | 5.89 | 1.61 | 1.28 | 2.04E-01 |
| 1313 | Frog | 5.23 | 1.81 | 3.85 | 2.04 | 3.86 | 1.85E-04 |
| 1321 | Bear | 4.97 | 1.74 | 6.34 | 1.94 | -4.01 | 1.08E-04 |
| 1333 | Parrots | 4.81 | 1.84 | 3.38 | 1.92 | 4.09 | 8.13E-05 |
| 1340 | Women | 3.84 | 1.77 | 3.89 | 2.2 | -0.13 | 9.00E-01 |
| 1390 | Bees | 4.78 | 2.01 | 5.15 | 2.14 | -0.95 | 3.42E-01 |
| 1419 | Bird | 4.69 | 1.65 | 3.73 | 1.84 | 2.95 | 3.80E-03 |
| 1440 | Seal | 5.5 | 1.89 | 4.76 | 2.25 | 1.93 | 5.60E-02 |
| **1450** | **Gannet** | **4.64** | **1.81** | **2.87** | **1.89** | **5.13** | **1.16E-06^*^** |
| 1460 | Kitten | 5.25 | 1.73 | 4.2 | 2.69 | 2.57 | 1.16E-02 |
| 1463 | Kittens | 5.75 | 1.95 | 4.46 | 2.17 | 3.37 | 1.01E-03 |
| 1500 | Dog | 4.66 | 1.75 | 4.08 | 2.58 | 1.45 | 1.50E-01 |
| 1510 | Dog | 4.36 | 1.93 | 4.15 | 2.38 | 0.53 | 5.99E-01 |
| 1525 | AttackDog | 5.72 | 1.8 | 6.14 | 2.31 | -1.11 | 2.71E-01 |
| 1540 | Cat | 5.25 | 1.81 | 4.33 | 2.28 | 2.43 | 1.65E-02 |
| 1560 | Hawk | 5.73 | 1.7 | 5.68 | 2.12 | 0.14 | 8.88E-01 |
| 1590 | Horse | 4.42 | 1.93 | 4.76 | 1.97 | -0.93 | 3.55E-01 |
| 1600 | Horse | 4.55 | 1.89 | 4.08 | 2.02 | 1.29 | 2.01E-01 |
| 1601 | Giraffes | 4.75 | 1.69 | 3.83 | 2.18 | 2.57 | 1.14E-02 |
| **1602** | **Butterfly** | **5.53** | **1.98** | **3.19** | **1.68** | **6.74** | **6.68E-10^*^** |
| **1603** | **Butterfly** | **4.92** | **1.85** | **3.47** | **1.25** | **4.78** | **5.18E-06^*^** |
| 1604 | Butterfly | 4.61 | 1.92 | 3.17 | 1.98 | 3.96 | 1.30E-04 |
| **1610** | **Rabbit** | **5.83** | **1.62** | **4.24** | **2.23** | **4.47** | **1.85E-05^*^** |
| 1616 | Bird | 4.7 | 1.86 | 3.6 | 1.91 | 3.14 | 2.15E-03 |
| 1620 | Sprgbok | 4.3 | 1.69 | 3.6 | 2.34 | 1.88 | 6.27E-02 |
| 1640 | Coyote | 4.77 | 1.93 | 5.13 | 2.18 | -0.94 | 3.47E-01 |
| 1650 | Jaguar | 5.17 | 1.8 | 6.4 | 1.99 | -3.49 | 6.77E-04 |
| 1660 | Gorilla | 4.97 | 1.56 | 4.45 | 2.38 | 1.42 | 1.58E-01 |
| 1661 | Orangutan | 4.58 | 1.81 | 3.79 | 2 | 2.23 | 2.75E-02 |
| 1670 | Cow | 3.45 | 7.82 | 2.95 | 2.67 | 0.43 | 6.66E-01 |
| 1710 | Puppies | 6.19 | 1.73 | 5.53 | 2.07 | 1.88 | 6.32E-02 |
| 1720 | Lion | 5.02 | 1.93 | 5.62 | 1.8 | -1.71 | 8.97E-02 |
| 1721 | Lion | 5.23 | 1.94 | 4.58 | 2.17 | 1.7 | 9.11E-02 |
| 1722 | Jaguars | 4.98 | 1.66 | 4.65 | 2.07 | 0.97 | 3.34E-01 |
| 1726 | Tiger | 5.25 | 1.79 | 6.63 | 2.26 | -3.68 | 3.52E-04 |
| 1731 | Lion | 5.52 | 1.94 | 4.64 | 2.44 | 2.16 | 3.28E-02 |
| 1740 | Owl | 4.31 | 1.74 | 4.81 | 2.21 | -1.37 | 1.74E-01 |
| 1750 | Bunnies | 4.98 | 1.73 | 4.21 | 2.22 | 2.11 | 3.71E-02 |
| 1810 | Hippo | 5.7 | 1.88 | 5 | 2.16 | 1.87 | 6.42E-02 |
| **1811** | **Monkies** | **6.94** | **1.55** | **5.05** | **1.84** | **6.02** | **2.12E-08^*^** |
| 1812 | Elephants | 4.91 | 1.85 | 3.53 | 2.28 | 3.61 | 4.52E-04 |
| 1850 | Camels | 4.28 | 1.8 | 4.38 | 1.98 | -0.28 | 7.79E-01 |
| **1900** | **Fish** | **5.11** | **2.02** | **3.04** | **2.07** | **5.43** | **3.19E-07^*^** |
| 1910 | Grouper | 4.47 | 1.81 | 3.96 | 2.26 | 1.35 | 1.78E-01 |
| 1920 | Porpoise | 5.25 | 1.88 | 4.21 | 2.49 | 2.58 | 1.12E-02 |
| 1930 | Shark | 5.23 | 0.77 | 5.98 | 2.24 | -2.55 | 1.21E-02 |
| 1931 | Shark | 5.81 | 1.77 | 6.88 | 1.77 | -3.23 | 1.59E-03 |
| 1932 | Shark | 5.61 | 2.18 | 6.21 | 2.18 | -1.47 | 1.43E-01 |
| 1935 | HermitCrab | 4.63 | 1.86 | 4.13 | 1.97 | 1.39 | 1.68E-01 |
| **1942** | **Tuetle** | **5.66** | **2.23** | **3.94** | **2.04** | **4.27** | **4.00E-05^*^** |
| 1945 | Tuetles | 5.13 | 1.9 | 4.27 | 2.02 | 2.34 | 2.07E-02 |
| 1947 | Octopus | 5.8 | 2.01 | 4.55 | 2.16 | 3.22 | 1.69E-03 |
| 1999 | Mickey | 4.95 | 1.69 | 4.52 | 2.31 | 1.16 | 2.47E-01 |
| **2000** | **Adult** | **4.58** | **1.45** | **2.92** | **1.75** | **5.6** | **1.45E-07^*^** |
| 2005 | AttractiveMan | 4.22 | 1.69 | 3.18 | 1.89 | 3.13 | 2.22E-03 |
| 2010 | Adult | 4.03 | 1.41 | 2.9 | 1.95 | 3.64 | 4.12E-04 |
| **2020** | **Adult** | **4.78** | **1.32** | **3.28** | **1.78** | **5.24** | **7.23E-07^*^** |
| 2025 | Women | 5 | 1.86 | 5.09 | 2.04 | -0.25 | 8.04E-01 |
| 2030 | Women | 5.13 | 1.81 | 6.24 | 1.83 | -3.27 | 1.44E-03 |
| 2040 | Baby | 5.44 | 1.91 | 4.33 | 2.19 | 2.92 | 4.21E-03 |
| 2050 | Baby | 5.29 | 1.86 | 4.05 | 2.3 | 3.22 | 1.64E-03 |
| 2053 | Baby | 6.23 | 1.75 | 4.65 | 2.41 | 4.11 | 7.46E-05 |
| 2055.1 | ManInPool | 5.34 | 1.98 | 4.61 | 2.02 | 1.97 | 5.16E-02 |
| **2057** | **Father** | **5.86** | **1.88** | **4.32** | **1.98** | **4.29** | **3.75E-05^*^** |
| 2058 | Baby | 4.89 | 2.02 | 4.47 | 2.16 | 1.08 | 2.82E-01 |
| **2070** | **Baby** | **6.59** | **1.6** | **4.02** | **2.3** | **7.13** | **9.65E-11^*^** |
| 2071 | Baby | 5.86 | 2.02 | 4.6 | 2.03 | 3.33 | 1.18E-03 |
| 2080 | Babies | 5.3 | 1.75 | 4.35 | 2.5 | 2.42 | 1.72E-02 |
| **2091** | **Girls** | **5.73** | **1.51** | **3.63** | **2.1** | **6.29** | **5.82E-09^*^** |
| 2092 | Clowns | 4.98 | 1.96 | 3.86 | 2.17 | 2.92 | 4.21E-03 |
| 2095 | Toddler | 6 | 2.03 | 4.69 | 2.11 | 3.39 | 9.46E-04 |
| **2100** | **AngryFace** | **5.52** | **1.79** | **3.77** | **2.59** | **4.32** | **3.32E-05^*^** |
| 2110 | AngryFace | 5.5 | 1.66 | 4.03 | 2.19 | 4.13 | 6.87E-05 |
| 2120 | AngryFace | 5.88 | 1.74 | 4.93 | 2.46 | 2.45 | 1.59E-02 |
| 2130 | Women | 5.19 | 2.01 | 4.09 | 2.22 | 2.79 | 6.13E-03 |
| 2141 | GrievingFem | 4.33 | 1.88 | 4.4 | 1.94 | -0.2 | 8.40E-01 |
| 2150 | Baby | 4.73 | 1.76 | 4.66 | 2.37 | 0.18 | 8.55E-01 |
| 2160 | Father | 4.98 | 1.63 | 5.31 | 2.1 | -0.96 | 3.41E-01 |
| **2165** | **Father** | **5.53** | **1.74** | **3.89** | **2.24** | **4.46** | **1.94E-05^*^** |
| 2170 | Mother | 5.27 | 2.02 | 3.9 | 2.12 | 3.55 | 5.53E-04 |
| **2190** | **Man** | **3.72** | **1.53** | **2.27** | **1.72** | **4.81** | **4.56E-06^*^** |
| 2191 | Farmer | 4 | 1.63 | 3.63 | 2.1 | 1.07 | 2.86E-01 |
| **2200** | **NeutFace** | **4.25** | **1.94** | **2.33** | **1.87** | **5.38** | **4.02E-07^*^** |
| 2205 | Hospital | 4.48 | 1.94 | 4.41 | 2.42 | 0.17 | 8.62E-01 |
| 2206 | Fingerprint | 4.08 | 1.6 | 3.56 | 2.18 | 1.48 | 1.40E-01 |
| 2208 | Bride | 4.52 | 1.75 | 4.94 | 2.25 | -1.15 | 2.54E-01 |
| 2209 | Bride | 4.38 | 1.62 | 5.04 | 2.23 | -1.85 | 6.66E-02 |
| **2210** | **NeutFace** | **5** | **1.97** | **2.66** | **1.71** | **6.72** | **7.38E-10^*^** |
| 2214 | NeutFace | 3.89 | 1.8 | 2.77 | 1.89 | 3.26 | 1.46E-03 |
| 2215 | NeutFace | 3.81 | 2.03 | 3.3 | 1.96 | 1.37 | 1.73E-01 |
| 2216 | Chidren | 5.16 | 1.71 | 5.08 | 2.14 | 0.22 | 8.23E-01 |
| 2220 | MaleFace | 4.7 | 1.95 | 5.04 | 1.43 | -1.04 | 3.00E-01 |
| 2221 | Judge | 4.05 | 1.53 | 3.11 | 2.4 | 2.58 | 1.10E-02 |
| 2222 | BoysReading | 4.2 | 1.61 | 3.72 | 1.97 | 1.46 | 1.47E-01 |
| 2224 | Boys | 4.2 | 1.7 | 4.59 | 2 | -1.13 | 2.61E-01 |
| 2230 | SadFace | 4.08 | 1.53 | 4.12 | 1.64 | -0.14 | 8.92E-01 |
| 2235 | Butcher | 3.86 | 1.85 | 3.34 | 1.85 | 1.5 | 1.36E-01 |
| 2240 | NeutChild | 4.47 | 1.75 | 3.24 | 1.87 | 3.65 | 3.93E-04 |
| 2250 | NeutBaby | 4.22 | 1.6 | 3.95 | 2.12 | 0.78 | 4.36E-01 |
| 2260 | NeutBaby | 4.52 | 1.58 | 3.74 | 2.09 | 2.28 | 2.42E-02 |
| 2270 | NeutChild | 3.84 | 1.54 | 3.07 | 1.87 | 2.44 | 1.60E-02 |
| 2271 | Women | 4.31 | 1.59 | 3.56 | 1.7 | 2.45 | 1.58E-02 |
| 2272 | LonelyBoy | 4.08 | 1.78 | 3.59 | 1.72 | 1.49 | 1.39E-01 |
| 2276 | Girl | 4.48 | 1.83 | 4.02 | 1.67 | 1.4 | 1.65E-01 |
| 2278 | Kids | 4.47 | 1.75 | 4.39 | 2 | 0.23 | 8.21E-01 |
| 2280 | Boy | 3.66 | 1.85 | 3.51 | 1.67 | 0.45 | 6.52E-01 |
| 2299 | Family | 4.42 | 1.64 | 3.97 | 1.87 | 1.39 | 1.68E-01 |
| 2303 | Children | 4.63 | 1.82 | 5 | 2.02 | -1.04 | 3.02E-01 |
| 2304 | Girl | 4.5 | 1.66 | 3.17 | 1.85 | 4.08 | 8.28E-05 |
| 2310 | Mother | 4.89 | 1.81 | 3.89 | 1.98 | 2.84 | 5.34E-03 |
| 2311 | Mother | 5.48 | 1.87 | 4.85 | 2.22 | 1.66 | 9.89E-02 |
| 2312 | Mother | 4.38 | 1.8 | 3.77 | 1.56 | 1.92 | 5.76E-02 |
| **2320** | **Girl** | **4.34** | **1.54** | **2.56** | **1.68** | **5.96** | **2.84E-08^*^** |
| 2331 | Chef | 4.7 | 1.62 | 3.87 | 2.17 | 2.38 | 1.90E-02 |
| 2340 | Family | 4.67 | 1.94 | 5.35 | 2.03 | -1.83 | 6.96E-02 |
| **2341** | **Children** | **5.17** | **1.72** | **3.59** | **2.18** | **4.38** | **2.61E-05^*^** |
| 2344 | Children | 4.95 | 1.56 | 4.11 | 1.72 | 2.76 | 6.78E-03 |
| 2345 | Children | 5.2 | 1.71 | 4.6 | 2.1 | 1.71 | 8.98E-02 |
| 2346 | Kids | 5.3 | 1.81 | 5.41 | 2.24 | -0.3 | 7.63E-01 |
| 2351 | NursingBaby | 4.83 | 1.76 | 4.62 | 2.04 | 0.59 | 5.56E-01 |
| 2352 | Kiss | 4.48 | 1.7 | 4.85 | 1.95 | -1.09 | 2.76E-01 |
| 2352.1 | Kiss | 5.09 | 1.81 | 4.98 | 1.97 | 0.32 | 7.46E-01 |
| **2352.2** | **BloodyKiss** | **7.52** | **1.94** | **5.78** | **1.94** | **4.78** | **5.18E-06^*^** |
| 2357 | Man | 3.33 | 1.81 | 3.06 | 1.84 | 0.8 | 4.23E-01 |
| 2360 | Family | 5.09 | 1.93 | 3.65 | 2.02 | 3.91 | 1.54E-04 |
| **2370** | **ThreeMan** | **4.48** | **1.79** | **2.85** | **2.07** | **4.56** | **1.31E-05^*^** |
| 2372 | Woman | 3.45 | 2 | 3.39 | 1.9 | 0.17 | 8.63E-01 |
| 2375.1 | Woman | 4.73 | 1.87 | 4.48 | 2.21 | 0.66 | 5.10E-01 |
| **2381** | **Girl** | **5.16** | **1.69** | **3.36** | **2.01** | **5.24** | **7.29E-07^*^** |
| 2383 | Secretary | 3.5 | 1.93 | 3.49 | 1.9 | 0.03 | 9.78E-01 |
| 2385 | Girl | 4.77 | 1.62 | 3.73 | 1.95 | 3.13 | 2.19E-03 |
| 2387 | Kids | 4.81 | 1.76 | 3.84 | 1.8 | 2.93 | 4.14E-03 |
| 2388 | Kids | 4.88 | 1.8 | 3.81 | 1.92 | 3.08 | 2.58E-03 |
| 2389 | Teens | 4.38 | 1.8 | 5.26 | 2.1 | -2.45 | 1.59E-02 |
| 2391 | Boy | 5.09 | 1.65 | 5.5 | 2.35 | -1.11 | 2.70E-01 |
| 2393 | Factoryworker | 3.94 | 1.95 | 2.9 | 1.8 | 2.94 | 3.96E-03 |
| 2394 | Medicalworker | 4.03 | 1.79 | 3.85 | 1.99 | 0.52 | 6.06E-01 |
| 2395 | Family | 4.3 | 1.86 | 3.88 | 2.03 | 1.15 | 2.51E-01 |
| 2399 | Woman | 4.28 | 1.39 | 3.72 | 1.93 | 1.83 | 7.02E-02 |
| 2410 | Boy | 4.36 | 1.57 | 3.83 | 2.16 | 1.54 | 1.27E-01 |
| 2435 | Mom/Son | 4.05 | 1.98 | 4.03 | 1.96 | 0.05 | 9.63E-01 |
| **2440** | **NeutGirl** | **4.11** | **1.72** | **2.44** | **1.57** | **5.39** | **3.79E-07^*^** |
| 2441 | NeutralGirl | 4.39 | 1.57 | 3.48 | 1.82 | 2.9 | 4.49E-03 |
| 2442 | DryingHair | 5.03 | 1.71 | 3.87 | 1.75 | 3.6 | 4.72E-04 |
| 2455 | SadGirls | 4.83 | 1.6 | 4.26 | 1.8 | 1.81 | 7.34E-02 |
| **2480** | **ElderlyMan** | **4.22** | **1.81** | **2.8** | **1.81** | **4.19** | **5.51E-05^*^** |
| 2485 | Man | 3.64 | 1.56 | 3.77 | 1.87 | -0.41 | 6.83E-01 |
| 2487 | Musician | 3.97 | 1.53 | 3.74 | 1.79 | 0.75 | 4.56E-01 |
| 2490 | Man | 3.91 | 1.97 | 3.83 | 2.24 | 0.2 | 8.38E-01 |
| 2491 | SickMan | 3.66 | 1.66 | 3.48 | 1.69 | 0.58 | 5.66E-01 |
| 2493 | NeutralMale | 4.03 | 1.73 | 3.13 | 1.94 | 2.64 | 9.38E-03 |
| 2495 | Man | 3.33 | 1.74 | 2.72 | 1.68 | 1.9 | 5.95E-02 |
| 2499 | NeutralMale | 4.3 | 1.95 | 3.18 | 1.78 | 3.18 | 1.89E-03 |
| 2500 | Man | 4.64 | 1.62 | 3.66 | 1.96 | 2.96 | 3.73E-03 |
| **2501** | **Couple** | **4.64** | **1.85** | **2.67** | **2.3** | **5.13** | **1.17E-06^*^** |
| 2510 | ElderlyWoman | 4.28 | 1.57 | 4.15 | 2.03 | 0.39 | 6.97E-01 |
| 2514 | Woman | 3.69 | 1.63 | 3.45 | 1.77 | 0.75 | 4.54E-01 |
| 2515 | Harvest | 3.92 | 1.59 | 3.62 | 2.08 | 0.89 | 3.75E-01 |
| 2516 | ElderlyWoman | 3.86 | 1.77 | 3.59 | 1.98 | 0.78 | 4.39E-01 |
| 2518 | Quilting | 3.78 | 1.81 | 3.21 | 1.91 | 1.65 | 1.03E-01 |
| 2520 | ElderlyMan | 4.05 | 1.4 | 4.19 | 1.88 | -0.46 | 6.45E-01 |
| 2530 | Couple | 4.86 | 1.69 | 4.23 | 2.03 | 1.83 | 6.99E-02 |
| **2540** | **Mother** | **5.95** | **1.81** | **4.23** | **2.47** | **4.35** | **2.98E-05^*^** |
| 2550 | Couple | 5.56 | 2.01 | 4.15 | 2.03 | 3.74 | 2.91E-04 |
| 2560 | Picnic | 4.11 | 1.59 | 3.42 | 2.02 | 2.07 | 4.09E-02 |
| **2570** | **Man** | **4.41** | **1.54** | **2.51** | **1.84** | **6.06** | **1.78E-08^*^** |
| 2575 | Propeller | 3.58 | 1.8 | 4.04 | 2.05 | -1.29 | 1.98E-01 |
| 2579 | Bakers | 4.8 | 2.21 | 3.91 | 1.96 | 2.26 | 2.60E-02 |
| 2580 | Chess | 3.98 | 1.74 | 2.72 | 1.79 | 3.83 | 2.11E-04 |
| 2590 | ElderlyWoman | 4.14 | 1.82 | 4 | 1.98 | 0.4 | 6.93E-01 |
| 2595 | Women | 3.39 | 1.64 | 3.8 | 1.88 | -1.25 | 2.12E-01 |
| 2600 | Beer | 3.92 | 1.73 | 4.37 | 1.59 | -1.44 | 1.52E-01 |
| 2616 | Dancer | 5.61 | 1.88 | 5.06 | 2.15 | 1.47 | 1.44E-01 |
| **2620** | **Woman** | **5.61** | **1.79** | **3.04** | **2.39** | **6.66** | **9.96E-10^*^** |
| **2630** | **Male** | **4.86** | **1.7** | **2.38** | **1.91** | **7.41** | **2.32E-11^*^** |
| 2635 | Cowboy | 4.27 | 1.81 | 4.45 | 2 | -0.52 | 6.03E-01 |
| 2650 | Boy | 5.55 | 1.73 | 4.25 | 2.48 | 3.34 | 1.12E-03 |
| 2655 | Child | 5.5 | 1.84 | 4.15 | 1.99 | 3.79 | 2.43E-04 |
| **2660** | **Baby** | **5.59** | **1.54** | **4.09** | **2.2** | **4.34** | **3.10E-05^*^** |
| 2661 | Baby | 5.95 | 1.82 | 5.18 | 2.09 | 2.12 | 3.58E-02 |
| 2681 | Police | 4.47 | 1.48 | 5.09 | 2.25 | -1.8 | 7.42E-02 |
| 2682 | Police | 4.95 | 1.86 | 4.43 | 2.33 | 1.35 | 1.80E-01 |
| 2683 | War | 5.48 | 2 | 5.99 | 2.07 | -1.35 | 1.78E-01 |
| 2688 | Hunters | 5.25 | 2.87 | 5.96 | 2.13 | -1.47 | 1.43E-01 |
| 2690 | Terrorist | 5.27 | 1.98 | 3.85 | 2 | 3.81 | 2.25E-04 |
| 2691 | Riot | 4.88 | 1.93 | 5.65 | 2.02 | -2.09 | 3.86E-02 |
| 2692 | Bomb | 4.63 | 1.79 | 5.11 | 2.17 | -1.31 | 1.92E-01 |
| 2694 | Police | 4.93 | 1.76 | 4.93 | 2.15 | -0.01 | 9.93E-01 |
| **2695** | **Refugees** | **5.81** | **1.73** | **4.26** | **1.92** | **4.59** | **1.16E-05^*^** |
| 2700 | Woman | 4.66 | 1.54 | 4.52 | 1.97 | 0.43 | 6.67E-01 |
| 2702 | BingeEating | 5.59 | 1.85 | 4.28 | 2.35 | 3.38 | 9.77E-04 |
| 2710 | DrugAddict | 4.88 | 1.56 | 5.29 | 2.33 | -1.14 | 2.57E-01 |
| 2715 | Smoking | 4.46 | 1.85 | 3.99 | 2.12 | 1.27 | 2.07E-01 |
| **2720** | **Urinating** | **5.59** | **2.13** | **3** | **1.9** | **6.81** | **4.71E-10^*^** |
| 2722 | Jail | 4.13 | 1.62 | 3.17 | 1.8 | 3.02 | 3.08E-03 |
| 2730 | NativeBoy | 6.5 | 1.92 | 6.6 | 2.25 | -0.26 | 7.96E-01 |
| 2745.1 | Shopping | 3.44 | 2.05 | 3.21 | 1.97 | 0.61 | 5.43E-01 |
| 2749 | Smoking | 3.31 | 1.66 | 3.7 | 2.07 | -1.12 | 2.64E-01 |
| **2750** | **Bum** | **5.59** | **1.85** | **4.06** | **1.93** | **4.34** | **3.03E-05^*^** |
| 2751 | DrunkDriving | 5.03 | 1.75 | 4.68 | 2.34 | 0.93 | 3.56E-01 |
| 2752 | Alcoholic | 4.84 | 1.71 | 4.11 | 1.98 | 2.13 | 3.49E-02 |
| 2753 | Alcoholic | 4.13 | 1.69 | 3.93 | 2.11 | 0.56 | 5.76E-01 |
| **2780** | **Actor** | **6.73** | **1.83** | **4.7** | **1.86** | **5.89** | **3.84E-08^*^** |
| 2791 | Balloons | 4.7 | 1.81 | 3.58 | 2.15 | 3.05 | 2.80E-03 |
| 2795 | Boy | 4.69 | 1.63 | 4.37 | 1.96 | 0.95 | 3.42E-01 |
| **2800** | **SadChild** | **7.28** | **1.65** | **4.94** | **1.94** | **7.04** | **1.49E-10^*^** |
| 2810 | Boy | 4.78 | 1.73 | 4.33 | 2.01 | 1.3 | 1.97E-01 |
| 2830 | Woman | 5.38 | 1.82 | 3.93 | 2.55 | 3.59 | 4.88E-04 |
| **2840** | **Chess** | **4.05** | **1.5** | **2.31** | **1.88** | **5.57** | **1.72E-07^*^** |
| **2850** | **Tourist** | **4.39** | **1.53** | **2.58** | **1.79** | **5.89** | **3.94E-08^*^** |
| 2870 | Teenager | 3.98 | 1.58 | 2.87 | 1.57 | 3.77 | 2.60E-04 |
| 2880 | Shadow | 3.66 | 1.64 | 2.68 | 2.93 | 2.3 | 2.33E-02 |
| 2890 | Twins | 4.09 | 1.59 | 3.02 | 1.94 | 3.28 | 1.39E-03 |
| 2900 | CryingBoy | 4.38 | 1.55 | 4.76 | 2.09 | -1.13 | 2.61E-01 |
| 2900.1 | FoodBasket | 4.36 | 1.52 | 4.12 | 2.06 | 0.73 | 4.69E-01 |
| 2900.2 | DeerHead | 4.78 | 1.6 | 4.23 | 1.66 | 1.82 | 7.21E-02 |
| 2980 | Mutilation | 4.14 | 1.93 | 3.09 | 1.75 | 3.03 | 2.99E-03 |
| 2981 | Mutilation | 5.59 | 1.92 | 5.54 | 1.97 | 0.15 | 8.83E-01 |
| **3000** | **Mutilation** | **8.48** | **0.98** | **6.92** | **2.44** | **4.75** | **5.91E-06^*^** |
| 3005.1 | OpenGrave | 7.02 | 1.97 | 5.55 | 2.55 | 3.51 | 6.49E-04 |
| 3010 | Mutilation | 7.19 | 2.29 | 6.83 | 2.26 | 0.85 | 3.99E-01 |
| 3015 | Accident | 6.91 | 2.17 | 5.54 | 2.74 | 3.01 | 3.23E-03 |
| 3022 | Scream | 5.11 | 2 | 5.61 | 1.67 | -1.44 | 1.54E-01 |
| 3030 | Mutilation | 6.16 | 1.7 | 6.39 | 2.26 | -0.63 | 5.31E-01 |
| **3051** | **Mutilation** | **7.27** | **1.72** | **5.23** | **2.46** | **5.28** | **6.22E-07^*^** |
| 3053 | BurnVictim | 7.61 | 1.81 | 6.2 | 2.71 | 3.37 | 1.02E-03 |
| 3060 | Mutilation | 7.77 | 1.81 | 6.89 | 2.08 | 2.44 | 1.62E-02 |
| **3061** | **Mutilation** | **8.14** | **1.19** | **5.19** | **2.36** | **8.84** | **1.27E-14^*^** |
| **3062** | **Mutilation** | **7.67** | **2** | **5.7** | **2.6** | **4.63** | **9.55E-06^*^** |
| **3063** | **Mutilation** | **7.8** | **1.85** | **5.44** | **2.78** | **5.5** | **2.28E-07^*^** |
| **3064** | **Mutilation** | **7.95** | **1.5** | **5.44** | **2.7** | **6.4** | **3.41E-09^*^** |
| 3068 | Mutilation | 6.59 | 2.28 | 6.44 | 2.46 | 0.34 | 7.34E-01 |
| 3069 | Mutilation | 7.98 | 1.54 | 6.7 | 2.6 | 3.33 | 1.18E-03 |
| 3071 | Mutilation | 7.55 | 1.82 | 6.61 | 2.13 | 2.57 | 1.15E-02 |
| 3080 | Mutilation | 6.97 | 1.87 | 6.84 | 2.06 | 0.36 | 7.22E-01 |
| 3100 | BurnVictim | 7.27 | 1.89 | 5.88 | 2.34 | 3.55 | 5.54E-04 |
| **3102** | **BurnVictim** | **8** | **1.23** | **5.88** | **2.79** | **5.55** | **1.90E-07^*^** |
| **3110** | **BurnVictim** | **8.11** | **1.3** | **6.43** | **2.26** | **5.07** | **1.55E-06^*^** |
| 3120 | DeadBoy | 7.33 | 1.55 | 6.2 | 2.55 | 2.97 | 3.64E-03 |
| 3130 | Mutilation | 6.84 | 1.95 | 6.56 | 2.11 | 0.74 | 4.60E-01 |
| **3140** | **DeadBoy** | **7.28** | **1.69** | **5.68** | **2.08** | **4.59** | **1.15E-05^*^** |
| **3150** | **Mutilation** | **7.84** | **1.44** | **6.1** | **2.29** | **5.03** | **1.81E-06^*^** |
| **3160** | **EyeDisease** | **7.13** | **1.57** | **5.23** | **1.88** | **5.95** | **2.92E-08^*^** |
| **3168** | **Mutilation** | **8.22** | **1.21** | **5.55** | **2.61** | **7.38** | **2.74E-11^*^** |
| **3170** | **BabyTumor** | **8.05** | **1.06** | **6.79** | **1.93** | **4.51** | **1.55E-05^*^** |
| 3180 | BatteredFem | 4.67 | 1.79 | 5.17 | 2.05 | -1.4 | 1.63E-01 |
| 3181 | BatteredFem | 6.08 | 1.88 | 4.9 | 2.17 | 3.14 | 2.16E-03 |
| 3190 | Scar | 5.45 | 1.44 | 5.17 | 1.79 | 0.94 | 3.51E-01 |
| 3210 | Surgery | 4.83 | 1.78 | 5.27 | 1.82 | -1.32 | 1.90E-01 |
| 3220 | Hospital | 5.8 | 1.84 | 5.18 | 1.89 | 1.77 | 7.89E-02 |
| 3230 | DyingMan | 5.48 | 1.82 | 5 | 2.35 | 1.25 | 2.15E-01 |
| 3250 | OpenChest | 6.34 | 1.99 | 6.1 | 1.75 | 0.68 | 4.99E-01 |
| **3261** | **Tumor** | **7.59** | **1.66** | **5.51** | **2.7** | **5.14** | **1.13E-06^*^** |
| **3266** | **Injury** | **7.41** | **1.54** | **5.85** | **2.21** | **4.5** | **1.64E-05^*^** |
| 3280 | DentalExam | 5.28 | 1.43 | 5.75 | 2.47 | -1.29 | 1.98E-01 |
| 3300 | DisabledChild | 4.83 | 1.59 | 4.14 | 2.07 | 2.04 | 4.37E-02 |
| 3301 | InjuredChild | 6.17 | 1.7 | 4.71 | 2.25 | 4 | 1.13E-04 |
| 3350 | Infant | 5.64 | 1.67 | 5.65 | 2.27 | -0.03 | 9.78E-01 |
| 3400 | SeveredHand | 7.67 | 1.52 | 6.67 | 2.29 | 2.84 | 5.40E-03 |
| **3500** | **Attack** | **4.38** | **2.02** | **6.8** | **2.04** | **-6.38** | **3.78E-09^*^** |
| 3530 | Attack | 6.34 | 1.64 | 6.85 | 2.13 | -1.46 | 1.46E-01 |
| 3550 | Injury | 7 | 1.86 | 5.69 | 2.41 | 3.32 | 1.22E-03 |
| 3550.1 | PlaneCrash | 6.11 | 2.15 | 5.8 | 1.97 | 0.8 | 4.27E-01 |
| 3550.2 | Coach | 4.69 | 1.77 | 4.66 | 2.32 | 0.07 | 9.42E-01 |
| 4000 | Artist | 5.06 | 1.68 | 4.31 | 2.12 | 2.14 | 3.49E-02 |
| 4001 | EroticFemale | 5.77 | 1.77 | 6.81 | 1.9 | -3.05 | 2.87E-03 |
| 4002 | EroticFemale | 5.8 | 1.86 | 7.15 | 1.81 | -3.93 | 1.46E-04 |
| 4003 | EroticFemale | 5.78 | 1.99 | 5.94 | 1.81 | -0.45 | 6.56E-01 |
| **4004** | **EroticFemale** | **4.98** | **1.62** | **2.28** | **2.14** | **7.77** | **3.61E-12^*^** |
| 4005 | EroticFemale | 5.19 | 1.93 | 5.77 | 1.87 | -1.64 | 1.04E-01 |
| 4100 | MaleDancers | 5.17 | 1.71 | 4.25 | 1.43 | 3.09 | 2.54E-03 |
| 4141 | EroticFemale | 6.72 | 1.82 | 6.73 | 1.94 | -0.03 | 9.77E-01 |
| 4142 | EroticFemale | 6.67 | 1.78 | 6.97 | 2.04 | -0.85 | 3.99E-01 |
| 4150 | AttractiveFem | 5.28 | 1.7 | 6.41 | 2.18 | -3.15 | 2.08E-03 |
| 4180 | EroticFemale | 6.89 | 1.51 | 7.43 | 1.97 | -1.68 | 9.59E-02 |
| **4210** | **EroticFemale** | **6.31** | **1.74** | **7.8** | **1.9** | **-4.41** | **2.38E-05^*^** |
| **4220** | **EroticFemale** | **5.73** | **1.71** | **7.17** | **1.76** | **-4.45** | **2.00E-05^*^** |
| 4230 | Prostitute | 6.47 | 1.67 | 5.23 | 2.12 | 3.54 | 5.88E-04 |
| **4232** | **EroticFemale** | **5.45** | **1.91** | **7.52** | **1.51** | **-6.32** | **5.20E-09^*^** |
| 4233 | Prostitute | 5.3 | 1.7 | 4.7 | 2.33 | 1.61 | 1.09E-01 |
| 4235 | EroticFemale | 6.27 | 1.8 | 6.73 | 2.33 | -1.22 | 2.26E-01 |
| 4240 | EroticFemale | 5.73 | 1.96 | 6.54 | 2.12 | -2.12 | 3.57E-02 |
| **4250** | **AttractiveFem** | **5.48** | **1.65** | **7.02** | **2.02** | **-4.54** | **1.42E-05^*^** |
| 4255 | EroticFemale | 5.86 | 1.62 | 6.47 | 2.27 | -1.7 | 9.25E-02 |
| 4274 | AttractiveFem | 5.53 | 1.89 | 5.2 | 2.44 | 0.82 | 4.11E-01 |
| 4275 | AttractiveFem | 6.53 | 1.53 | 6 | 2.16 | 1.55 | 1.23E-01 |
| 4279 | EroticFemale | 5.09 | 1.85 | 6.38 | 2.05 | -3.55 | 5.50E-04 |
| 4290 | EroticFemale | 5.84 | 1.95 | 7.2 | 1.87 | -3.8 | 2.35E-04 |
| 4300 | EroticFemale | 7.49 | 1.68 | 7.23 | 1.98 | 0.77 | 4.41E-01 |
| 4302 | EroticFemale | 6.33 | 1.7 | 6.93 | 2.32 | -1.62 | 1.08E-01 |
| 4310 | EroticFemale | 6.52 | 1.8 | 6.89 | 1.87 | -1.08 | 2.81E-01 |
| 4320 | EroticFemale | 6.72 | 1.41 | 6.37 | 2.09 | 1.08 | 2.82E-01 |
| 4460 | EroticMale | 5.43 | 1.92 | 3.92 | 1.99 | 4.14 | 6.71E-05 |
| **4470** | **EroticMale** | **4.8** | **1.77** | **3.31** | **2.02** | **4.24** | **4.55E-05^*^** |
| **4490** | **EroticMale** | **4.64** | **1.9** | **2.85** | **1.96** | **4.97** | **2.32E-06^*^** |
| **4500** | **AttractiveMan** | **5.16** | **1.84** | **2.33** | **2.89** | **6.45** | **2.72E-09^*^** |
| **4503** | **EroticMale** | **6.2** | **1.94** | **3.9** | **2.14** | **6.07** | **1.69E-08^*^** |
| **4510** | **AttractiveMan** | **4.23** | **1.73** | **1.78** | **1.48** | **8.05** | **8.34E-13^*^** |
| 4520 | EroticMale | 4.28 | 1.96 | 3.71 | 1.93 | 1.57 | 1.20E-01 |
| **4530** | **EroticMale** | **4.84** | **1.52** | **3.15** | **2.06** | **5.11** | **1.31E-06^*^** |
| **4531** | **EroticMale** | **5.03** | **1.83** | **2.73** | **2.14** | **6.25** | **7.06E-09^*^** |
| **4532** | **AttractiveMan** | **4.64** | **1.75** | **2.73** | **1.74** | **5.85** | **4.62E-08^*^** |
| 4533 | AttractiveMan | 5.02 | 1.79 | 4.12 | 2.37 | 2.33 | 2.15E-02 |
| **4534** | **MaleDancers** | **5.19** | **1.49** | **3.11** | **2.06** | **6.34** | **4.63E-09^*^** |
| **4535** | **Weightlifter** | **5.7** | **1.6** | **4.19** | **2.24** | **4.25** | **4.30E-05^*^** |
| **4536** | **AttractiveMan** | **4.97** | **1.81** | **2.96** | **1.92** | **5.79** | **6.13E-08^*^** |
| 4537 | AttractiveMan | 4.48 | 1.7 | 3.32 | 1.9 | 3.47 | 7.30E-04 |
| 4538 | EroticMale | 4.13 | 1.42 | 2.9 | 1.82 | 4.11 | 7.54E-05 |
| **4550** | **EroticMale** | **5.61** | **2.15** | **3.34** | **2.5** | **5.27** | **6.51E-07^*^** |
| **4561** | **EroticMale** | **5.34** | **1.64** | **2.64** | **1.95** | **8.12** | **5.76E-13^*^** |
| **4571** | **AttractiveMan** | **4.84** | **1.65** | **2.31** | **1.85** | **7.8** | **3.12E-12^*^** |
| **4572** | **AttractiveMan** | **4.5** | **1.59** | **2.79** | **2.04** | **5.1** | **1.38E-06^*^** |
| 4598 | Couple | 5.97 | 1.61 | 5.25 | 2.25 | 2.01 | 4.63E-02 |
| 4599 | Romance | 5.75 | 1.51 | 5.73 | 1.93 | 0.06 | 9.50E-01 |
| 4601 | Romance | 6.34 | 1.54 | 5.25 | 2.02 | 3.32 | 1.22E-03 |
| 4603 | Romance | 5.33 | 1.72 | 4.76 | 2.05 | 1.63 | 1.06E-01 |
| 4605 | Couple | 4.31 | 1.5 | 4.38 | 2.42 | -0.19 | 8.48E-01 |
| 4606 | Romance | 5.34 | 1.64 | 5.1 | 2.06 | 0.71 | 4.79E-01 |
| 4607 | EroticCouple | 6.39 | 1.62 | 7.19 | 1.88 | -2.47 | 1.51E-02 |
| 4608 | EroticCouple | 5.83 | 1.54 | 6.84 | 1.63 | -3.43 | 8.49E-04 |
| 4609 | Couple | 5.88 | 1.32 | 5.35 | 1.9 | 1.78 | 7.73E-02 |
| 4610 | Romance | 4.75 | 1.69 | 5.17 | 2.06 | -1.21 | 2.29E-01 |
| 4611 | EroticCouple | 6.05 | 1.61 | 6.5 | 2.02 | -1.34 | 1.83E-01 |
| 4613 | Condom | 5.47 | 1.56 | 4.84 | 2.19 | 1.82 | 7.17E-02 |
| 4614 | Romance | 5.23 | 1.77 | 3.72 | 2.27 | 4.04 | 9.57E-05 |
| 4617 | EroticFemale | 4.88 | 1.89 | 4.88 | 2.29 | -0.01 | 9.90E-01 |
| 4621 | Harassment | 4.48 | 1.37 | 4.49 | 1.97 | -0.03 | 9.74E-01 |
| 4622 | Romance | 4.47 | 1.6 | 4.11 | 2.04 | 1.07 | 2.89E-01 |
| 4623 | Romance | 5.56 | 1.74 | 5.51 | 2.01 | 0.15 | 8.81E-01 |
| 4624 | Couple | 4.6 | 1.76 | 4.8 | 1.86 | -0.58 | 5.61E-01 |
| 4625 | Couple | 4.52 | 1.69 | 4.96 | 1.99 | -1.3 | 1.95E-01 |
| 4626 | Wedding | 5.59 | 1.84 | 5.45 | 2.28 | 0.38 | 7.07E-01 |
| 4631 | BikerCouple | 5.52 | 1.75 | 5.86 | 2.05 | -0.98 | 3.30E-01 |
| 4635 | Prostitute | 4.95 | 1.97 | 4.05 | 2.23 | 2.31 | 2.27E-02 |
| 4640 | Romance | 4.8 | 1.67 | 5.07 | 1.98 | -0.8 | 4.26E-01 |
| 4641 | Romance | 5.48 | 1.59 | 5.53 | 2.1 | -0.15 | 8.84E-01 |
| 4650 | EroticCouple | 5.06 | 1.7 | 5.9 | 2.1 | -2.39 | 1.84E-02 |
| 4651 | EroticCouple | 6.61 | 1.22 | 6.96 | 1.95 | -1.2 | 2.34E-01 |
| 4652 | EroticCouple | 7.41 | 1.67 | 7.25 | 1.64 | 0.5 | 6.15E-01 |
| 4653 | EroticCouple | 5.91 | 1.59 | 5.98 | 1.97 | -0.21 | 8.32E-01 |
| 4656 | EroticCouple | 6.14 | 1.8 | 6.46 | 2.53 | -0.8 | 4.26E-01 |
| 4658 | EroticCouple | 7.27 | 1.61 | 6.89 | 2.06 | 1.11 | 2.71E-01 |
| 4659 | EroticCouple | 6.64 | 1.68 | 7.43 | 1.8 | -2.44 | 1.62E-02 |
| 4660 | EroticCouple | 6.11 | 1.68 | 6.92 | 1.74 | -2.54 | 1.24E-02 |
| 4664 | EroticCouple | 6.89 | 1.61 | 7.72 | 1.45 | -2.88 | 4.79E-03 |
| 4664.1 | Erotic | 6.84 | 1.95 | 7.46 | 1.63 | -1.82 | 7.10E-02 |
| 4664.2 | Attack | 6.3 | 1.96 | 6.29 | 2.02 | 0.03 | 9.79E-01 |
| 4666 | EroticCouple | 5.94 | 1.83 | 6.48 | 2.12 | -1.48 | 1.41E-01 |
| 4669 | EroticCouple | 6.34 | 1.69 | 6.44 | 2.43 | -0.26 | 7.93E-01 |
| 4670 | EroticCouple | 7.06 | 1.62 | 7.17 | 1.93 | -0.33 | 7.43E-01 |
| 4672 | EroticCouple | 6.84 | 1.77 | 6.42 | 2.4 | 1.09 | 2.78E-01 |
| 4676 | EroticCouple | 5.48 | 1.92 | 6.21 | 2.11 | -1.94 | 5.50E-02 |
| 4677 | EroticCouple | 5.09 | 2.01 | 5.97 | 2.12 | -2.28 | 2.44E-02 |
| 4680 | EroticCouple | 6.61 | 1.96 | 5.94 | 2.3 | 1.7 | 9.22E-02 |
| **4681** | **EroticCouple** | **5.7** | **1.97** | **7.13** | **1.33** | **-4.43** | **2.16E-05^*^** |
| 4683 | EroticCouple | 6.08 | 1.85 | 7.28 | 1.62 | -3.66 | 3.86E-04 |
| 4687 | EroticCouple | 5.53 | 2.05 | 6.54 | 2.19 | -2.55 | 1.20E-02 |
| 4689 | EroticCouple | 5.5 | 1.84 | 6.26 | 1.8 | -2.23 | 2.78E-02 |
| 4690 | EroticCouple | 6.22 | 1.81 | 6.46 | 2.22 | -0.64 | 5.21E-01 |
| 4700 | Couple | 4.34 | 1.6 | 4 | 1.89 | 1.05 | 2.95E-01 |
| 4750 | NudeFemale | 7.13 | 2.02 | 5.79 | 2.03 | 3.53 | 6.00E-04 |
| 4770 | FemaleKiss | 6.03 | 1.56 | 6.52 | 1.92 | -1.52 | 1.31E-01 |
| 4800 | EroticCouple | 6.77 | 1.76 | 7.76 | 1.33 | -3.33 | 1.17E-03 |
| 4810 | EroticCouple | 6.61 | 2.05 | 6.89 | 2.23 | -0.7 | 4.83E-01 |
| **5000** | **Flower** | **4.23** | **1.73** | **2.44** | **2.06** | **5.1** | **1.35E-06^*^** |
| 5001 | SunFlower | 4.09 | 1.81 | 3.64 | 2.16 | 1.22 | 2.23E-01 |
| 5010 | Flower | 4.09 | 1.71 | 2.78 | 2.07 | 3.74 | 2.84E-04 |
| 5020 | Flower | 3.92 | 1.66 | 2.58 | 2.29 | 3.67 | 3.68E-04 |
| **5030** | **Flower** | **4.78** | **1.71** | **2.43** | **2** | **6.84** | **4.11E-10^*^** |
| 5120 | PineNeedles | 4.31 | 2.1 | 2.85 | 2.04 | 3.77 | 2.57E-04 |
| **5130** | **Rocks** | **3.94** | **1.84** | **2.33** | **1.5** | **5.06** | **1.62E-06^*^** |
| 5200 | Flowers | 4.48 | 1.94 | 3.46 | 2.06 | 2.74 | 7.13E-03 |
| 5201 | Nature | 4.89 | 2.06 | 3.9 | 2.24 | 2.48 | 1.47E-02 |
| 5220 | Nature | 4.91 | 1.92 | 4.58 | 2.24 | 0.86 | 3.94E-01 |
| 5250 | Nature | 4.78 | 1.86 | 3.92 | 2.18 | 2.3 | 2.34E-02 |
| 5260 | Waterfall | 6.31 | 1.77 | 6 | 2.5 | 0.79 | 4.34E-01 |
| 5270 | Nature | 6.49 | 1.6 | 5.24 | 2.45 | 3.33 | 1.16E-03 |
| 5300 | Galaxy | 6.34 | 1.78 | 4.75 | 2.56 | 3.96 | 1.29E-04 |
| **5390** | **Boat** | **4.69** | **1.82** | **2.95** | **1.83** | **5.1** | **1.33E-06^*^** |
| 5395 | Boat | 4.69 | 1.89 | 4.21 | 2.17 | 1.27 | 2.08E-01 |
| 5410 | Violinist | 4.25 | 1.63 | 3.42 | 1.86 | 2.56 | 1.16E-02 |
| 5450 | Liftoff | 6.67 | 1.62 | 5.72 | 2.36 | 2.58 | 1.12E-02 |
| 5455 | Cockpit | 4.11 | 1.9 | 4.3 | 2.03 | -0.52 | 6.07E-01 |
| 5460 | Astronaut | 4.42 | 1.7 | 6 | 2.45 | -4.12 | 7.24E-05 |
| 5470 | Astronaut | 5.91 | 1.74 | 6.44 | 2.4 | -1.39 | 1.69E-01 |
| 5480 | Fireworks | 5.8 | 1.75 | 5.55 | 2.3 | 0.67 | 5.06E-01 |
| 5500 | Mushroom | 3.89 | 1.9 | 2.82 | 2.58 | 2.58 | 1.10E-02 |
| **5510** | **Mushroom** | **4.45** | **1.89** | **2.78** | **2.29** | **4.32** | **3.30E-05^*^** |
| 5520 | Mushroom | 3.8 | 1.84 | 2.95 | 2.63 | 2.06 | 4.20E-02 |
| 5530 | Mushroom | 3.75 | 1.75 | 2.87 | 2.47 | 2.26 | 2.60E-02 |
| 5531 | Mushroom | 4.33 | 1.94 | 3.6 | 2.15 | 1.92 | 5.71E-02 |
| 5532 | Mushrooms | 3.44 | 1.72 | 4.01 | 2.18 | -1.58 | 1.17E-01 |
| 5533 | Mushrooms | 4.52 | 1.76 | 3.08 | 2.02 | 4.11 | 7.47E-05 |
| 5534 | Mushrooms | 3.64 | 1.99 | 2.88 | 2.18 | 1.96 | 5.22E-02 |
| 5535 | Stilllife | 4.36 | 1.66 | 3.85 | 1.96 | 1.52 | 1.31E-01 |
| 5551 | Clouds | 4.75 | 1.86 | 3.28 | 2.01 | 4.09 | 8.18E-05 |
| 5593 | Sky | 5.2 | 1.95 | 4.45 | 2.23 | 1.94 | 5.54E-02 |
| 5594 | Sky | 5.33 | 2.07 | 4.28 | 2.61 | 2.42 | 1.71E-02 |
| 5600 | Moutains | 6.23 | 1.64 | 5.93 | 2.3 | 0.82 | 4.12E-01 |
| 5611 | Moutains | 4.98 | 1.73 | 4.47 | 2.5 | 1.31 | 1.91E-01 |
| 5621 | SkyDivers | 5.94 | 1.88 | 6.96 | 1.72 | -3.01 | 3.22E-03 |
| 5622 | Shark | 5.5 | 1.75 | 5.38 | 1.89 | 0.35 | 7.24E-01 |
| 5623 | Windsurfers | 5.86 | 1.68 | 5.56 | 2.3 | 0.82 | 4.16E-01 |
| 5626 | HangGlider | 5.73 | 2.09 | 6.23 | 2.29 | -1.23 | 2.21E-01 |
| 5628 | Moutains | 5.17 | 1.96 | 5.54 | 2.13 | -0.97 | 3.32E-01 |
| 5629 | Hiker | 5.69 | 1.94 | 6.59 | 2.2 | -2.34 | 2.08E-02 |
| 5660 | Moutains | 5.64 | 2.02 | 5.25 | 2.51 | 0.93 | 3.54E-01 |
| 5661 | Cave | 4.91 | 2.29 | 4.36 | 2.2 | 1.3 | 1.97E-01 |
| 5700 | Moutains | 5.47 | 1.84 | 5.94 | 2.28 | -1.23 | 2.20E-01 |
| 5711 | Field | 4.47 | 1.89 | 3.47 | 1.67 | 2.97 | 3.58E-03 |
| **5720** | **Farmland** | **4.91** | **2.01** | **2.8** | **2.15** | **5.45** | **2.88E-07^*^** |
| **5731** | **Flowers** | **4.05** | **1.92** | **2.44** | **1.87** | **4.54** | **1.41E-05^*^** |
| 5740 | Plant | 3.75 | 1.85 | 2.36 | 1.77 | 4.09 | 7.88E-05 |
| 5750 | Nature | 4.5 | 2.07 | 3.33 | 2.3 | 2.88 | 4.69E-03 |
| **5760** | **Nature** | **5.31** | **1.82** | **2.77** | **2.16** | **6.89** | **3.13E-10^*^** |
| 5779 | Courtyard | 4.92 | 2.03 | 3.62 | 2.11 | 3.37 | 1.02E-03 |
| 5780 | Nature | 4.94 | 1.93 | 4.13 | 2.6 | 1.93 | 5.55E-02 |
| **5800** | **Leaves** | **4.63** | **1.88** | **2.54** | **2.22** | **5.5** | **2.28E-07^*^** |
| 5811 | Flowers | 4.48 | 1.7 | 3.49 | 1.92 | 2.95 | 3.88E-03 |
| 5820 | Moutains | 5.14 | 2.01 | 4.9 | 2.47 | 0.58 | 5.64E-01 |
| **5830** | **Sunset** | **6.58** | **1.73** | **4.98** | **2.4** | **4.2** | **5.36E-05^*^** |
| 5831 | Seagulls | 5.3 | 1.69 | 3.93 | 2.28 | 3.73 | 2.95E-04 |
| 5849 | Flowers | 4.59 | 2.05 | 4.11 | 2.1 | 1.25 | 2.14E-01 |
| 5870 | Clouds | 4.52 | 1.83 | 3.75 | 2.29 | 2.02 | 4.58E-02 |
| 5875 | Bicyclist | 4.03 | 1.72 | 3.36 | 1.99 | 1.95 | 5.32E-02 |
| 5890 | Earth | 5.66 | 1.9 | 5.17 | 2.37 | 1.24 | 2.17E-01 |
| 5891 | Clouds | 4.63 | 2.08 | 3.46 | 2.55 | 2.73 | 7.30E-03 |
| 5900 | Desert | 4.75 | 1.88 | 4.71 | 2.03 | 0.11 | 9.13E-01 |
| 5910 | Fireworks | 4.64 | 1.91 | 5.37 | 2.32 | -1.86 | 6.47E-02 |
| 5920 | Volcano | 5.89 | 1.76 | 6.25 | 2.25 | -0.97 | 3.34E-01 |
| 5940 | Lava | 5.72 | 1.73 | 6.42 | 1.63 | -2.22 | 2.85E-02 |
| **5950** | **Lightning** | **4.28** | **1.8** | **6.87** | **2.07** | **-7.23** | **5.75E-11^*^** |
| 5970 | Tornado | 5.39 | 1.85 | 4.65 | 2.61 | 1.8 | 7.52E-02 |
| 5971 | Tornado | 5.06 | 1.94 | 6.46 | 2.02 | -3.78 | 2.46E-04 |
| 5972 | Tornado | 4.86 | 1.82 | 6.38 | 2.13 | -4.15 | 6.40E-05 |
| 5982 | Sky | 5.36 | 1.91 | 5.25 | 2.75 | 0.25 | 8.01E-01 |
| 5990 | Sky | 5.81 | 1.88 | 4.28 | 2.35 | 3.92 | 1.53E-04 |
| 5991 | Sky | 4.89 | 2.01 | 4.37 | 2.37 | 1.28 | 2.02E-01 |
| 5994 | Skyline | 4.81 | 1.76 | 4.3 | 2.24 | 1.38 | 1.71E-01 |
| 6000 | Prison | 3.89 | 1.68 | 4.83 | 2.05 | -2.72 | 7.46E-03 |
| 6010 | Jail | 4.75 | 1.56 | 3.79 | 1.81 | 3.07 | 2.64E-03 |
| 6020 | ElectricChair | 5.12 | 1.97 | 5.23 | 2.21 | -0.28 | 7.77E-01 |
| 6150 | Outlet | 3.58 | 2.07 | 3.62 | 2.13 | -0.1 | 9.19E-01 |
| 6190 | AimedGun | 4.78 | 1.61 | 4.83 | 1.81 | -0.15 | 8.77E-01 |
| 6200 | AimedGun | 4.28 | 1.75 | 5.29 | 2.1 | -2.83 | 5.43E-03 |
| 6210 | AimedGun | 4.41 | 2.73 | 5.98 | 2.03 | -3.42 | 8.64E-04 |
| 6211 | Attack | 5.38 | 1.75 | 5.38 | 2.22 | 0 | 1.00E+00 |
| 6212 | Soldier | 5.25 | 2.11 | 5.47 | 2.44 | -0.52 | 6.03E-01 |
| 6213 | Terrorist | 4.56 | 1.7 | 5.25 | 1.93 | -2.05 | 4.27E-02 |
| 6230 | AimedGun | 5.86 | 1.82 | 7.1 | 2.07 | -3.44 | 7.99E-04 |
| 6241 | Gun | 3.84 | 1.88 | 4.13 | 2.36 | -0.74 | 4.61E-01 |
| 6243 | AimedGun | 5.58 | 1.6 | 5.6 | 2.34 | -0.06 | 9.52E-01 |
| 6244 | AimedGun | 6.24 | 1.74 | 5.63 | 2.39 | 1.59 | 1.14E-01 |
| **6250** | **AimedGun** | **4.97** | **1.8** | **6.75** | **2.74** | **-4.24** | **4.61E-05^*^** |
| 6250.2 | IceCream | 5.58 | 1.88 | 6.53 | 2.07 | -2.59 | 1.08E-02 |
| **6260** | **AimedGun** | **5.19** | **1.86** | **7.1** | **1.9** | **-5.44** | **2.98E-07^*^** |
| 6300 | Knife | 5.98 | 2.19 | 6.37 | 1.73 | -1.03 | 3.06E-01 |
| 6311 | DistressedFem | 4.8 | 1.77 | 4.77 | 1.98 | 0.07 | 9.41E-01 |
| 6312 | Abduction | 5.5 | 1.81 | 5.9 | 2.35 | -1.04 | 3.00E-01 |
| 6313 | Attack | 6.13 | 1.71 | 6.54 | 2.11 | -1.17 | 2.43E-01 |
| **6314** | **Attack** | **5.8** | **1.53** | **1.07** | **2.28** | **13.41** | **2.95E-25^*^** |
| 6315 | BeatenFem | 5.73 | 1.8 | 6.04 | 2.16 | -0.83 | 4.06E-01 |
| 6350 | Attack | 5.92 | 1.76 | 7.04 | 1.73 | -3.43 | 8.48E-04 |
| 6360 | Attack | 5.23 | 2.03 | 5.8 | 2.45 | -1.37 | 1.75E-01 |
| 6370 | Attack | 6.3 | 1.88 | 6.28 | 1.97 | 0.06 | 9.56E-01 |
| 6410 | AimedGun | 4.28 | 1.61 | 5.6 | 2.11 | -3.84 | 2.02E-04 |
| 6415 | DeadTiger | 5.91 | 1.72 | 5.86 | 2.27 | 0.12 | 9.01E-01 |
| 6510 | Attack | 6.14 | 1.89 | 6.67 | 2.33 | -1.36 | 1.77E-01 |
| 6530 | Attack | 4.98 | 1.77 | 6.02 | 2.09 | -2.9 | 4.49E-03 |
| 6540 | Attack | 5.58 | 1.68 | 6.51 | 2.27 | -2.55 | 1.20E-02 |
| 6550 | Attack | 6.61 | 1.68 | 6.98 | 2.13 | -1.05 | 2.94E-01 |
| 6555 | Knife | 5.41 | 1.87 | 5.6 | 1.92 | -0.54 | 5.92E-01 |
| 6560 | Attack | 5.88 | 1.66 | 6.17 | 2.28 | -0.81 | 4.20E-01 |
| 6561 | Attack | 5.02 | 1.36 | 4.44 | 2.15 | 1.77 | 7.98E-02 |
| 6570 | Suicide | 5.98 | 2.01 | 6.06 | 2.44 | -0.18 | 8.54E-01 |
| 6570.1 | Suicide | 6.11 | 2.13 | 5.76 | 2.02 | 0.9 | 3.72E-01 |
| 6570.2 | BlowDry | 4.31 | 2.05 | 3.62 | 1.98 | 1.84 | 6.89E-02 |
| 6571 | CarTheft | 5.57 | 1.81 | 5.22 | 2.24 | 0.93 | 3.52E-01 |
| 6610 | Gun | 3.61 | 1.65 | 4.69 | 2.28 | -2.97 | 3.59E-03 |
| 6800 | Gun | 4.14 | 1.74 | 4.85 | 2.45 | -1.83 | 6.94E-02 |
| 6821 | Gang | 5.83 | 1.55 | 5.93 | 2.1 | -0.3 | 7.67E-01 |
| 6830 | Guns | 4.81 | 1.66 | 5.61 | 2.19 | -2.24 | 2.69E-02 |
| 6831 | Police | 5.33 | 1.64 | 5 | 2.15 | 0.94 | 3.51E-01 |
| 6834 | Police | 4.53 | 1.75 | 5.77 | 1.76 | -3.77 | 2.57E-04 |
| 6836 | Police | 4.55 | 1.98 | 4.96 | 2.06 | -1.1 | 2.74E-01 |
| 6838 | Police | 4.67 | 1.75 | 5.38 | 2.26 | -1.91 | 5.83E-02 |
| **6840** | **Police** | **4.45** | **1.8** | **5.96** | **1.74** | **-4.55** | **1.36E-05^*^** |
| 6900 | Aircraft | 5.75 | 1.88 | 6.02 | 2.25 | -0.71 | 4.82E-01 |
| 6910 | Bomber | 6.44 | 1.51 | 6.26 | 2.47 | 0.48 | 6.33E-01 |
| 6930 | Missiles | 4.69 | 1.74 | 5.17 | 1.97 | -1.39 | 1.66E-01 |
| 6940 | Tank | 6.27 | 1.65 | 5.17 | 2.14 | 3.14 | 2.15E-03 |
| 7000 | RollingPin | 3.19 | 1.84 | 2.73 | 1.86 | 1.33 | 1.86E-01 |
| 7002 | Towel | 2.75 | 1.78 | 2.99 | 1.81 | -0.72 | 4.75E-01 |
| 7004 | Spoon | 3.5 | 2.02 | 2.09 | 1.75 | 3.95 | 1.35E-04 |
| 7006 | Bowl | 3.17 | 1.82 | 2.08 | 1.58 | 3.39 | 9.67E-04 |
| 7009 | Mug | 3.34 | 1.94 | 2.69 | 1.95 | 1.79 | 7.63E-02 |
| **7010** | **Basket** | **2.95** | **1.73** | **1.55** | **1.36** | **4.74** | **6.16E-06^*^** |
| **7020** | **Fan** | **3.45** | **1.56** | **2.15** | **1.71** | **4.28** | **3.90E-05^*^** |
| 7025 | Stool | 3.23 | 1.89 | 2.44 | 2.27 | 2.06 | 4.15E-02 |
| 7030 | Iron | 3.3 | 2.14 | 2.76 | 2.13 | 1.35 | 1.79E-01 |
| **7031** | **Shoes** | **3.58** | **1.93** | **1.67** | **1.25** | **6.1** | **1.48E-08^*^** |
| 7034 | Hammer | 3.53 | 2.1 | 3.15 | 1.93 | 1 | 3.18E-01 |
| 7035 | Mug | 3.52 | 1.66 | 2.56 | 1.8 | 2.98 | 3.48E-03 |
| 7036 | Shipyard | 3.52 | 1.7 | 3.47 | 2.09 | 0.14 | 8.87E-01 |
| 7037 | Trains | 3.92 | 1.89 | 3.73 | 1.96 | 0.54 | 5.94E-01 |
| 7038 | Shoes | 3.27 | 1.78 | 2.92 | 1.84 | 1.04 | 3.02E-01 |
| 7039 | Train | 4.7 | 1.85 | 3.32 | 1.95 | 3.89 | 1.68E-04 |
| 7040 | DustPan | 3.08 | 1.81 | 2.46 | 1.86 | 1.81 | 7.27E-02 |
| 7041 | Baskets | 3.45 | 1.96 | 2.68 | 2.76 | 1.77 | 7.90E-02 |
| 7050 | HairDryer | 3.16 | 1.64 | 2.59 | 1.79 | 1.79 | 7.64E-02 |
| 7060 | TrashCan | 3.92 | 1.83 | 2.71 | 1.75 | 3.61 | 4.59E-04 |
| 7080 | Fork | 3 | 1.97 | 1.98 | 1.63 | 2.98 | 3.54E-03 |
| 7090 | Book | 3.08 | 1.83 | 2.3 | 1.9 | 2.24 | 2.68E-02 |
| 7095 | Headlight | 3.67 | 1.89 | 4.45 | 2.36 | -1.98 | 4.99E-02 |
| 7096 | Car | 4.13 | 1.95 | 4.04 | 1.8 | 0.24 | 8.10E-01 |
| 7100 | FireHydrant | 3.33 | 1.82 | 3.08 | 1.67 | 0.76 | 4.51E-01 |
| **7110** | **Hammer** | **3.45** | **1.84** | **1.91** | **1.39** | **4.95** | **2.52E-06^*^** |
| 7130 | Truck | 3.39 | 1.72 | 3.54 | 2.01 | -0.43 | 6.65E-01 |
| 7140 | Bus | 3.53 | 1.76 | 2.67 | 2.33 | 2.28 | 2.47E-02 |
| 7150 | Umbrella | 3.45 | 1.73 | 2.66 | 1.68 | 2.48 | 1.45E-02 |
| **7160** | **Fabric** | **4.75** | **2.23** | **3.06** | **2.08** | **4.17** | **5.88E-05^*^** |
| 7161 | Pole | 3.41 | 1.74 | 2.79 | 2.81 | 1.46 | 1.47E-01 |
| 7170 | LightBulb | 4.14 | 1.84 | 3.15 | 1.85 | 2.87 | 4.85E-03 |
| **7175** | **Lamp** | **3.61** | **1.73** | **1.55** | **0.96** | **7.58** | **9.76E-12^*^** |
| 7179 | Rug | 4 | 1.89 | 2.87 | 2.05 | 3.09 | 2.55E-03 |
| 7180 | NeonBuilding | 4.47 | 1.99 | 3.41 | 2.04 | 2.81 | 5.76E-03 |
| 7182 | Checkerboard | 5.34 | 2.02 | 4.02 | 2.15 | 3.4 | 9.22E-04 |
| 7183 | Checkerboard | 5.36 | 2 | 3.82 | 2.26 | 3.9 | 1.64E-04 |
| 7184 | AbstractArt | 4.34 | 1.99 | 3.44 | 1.93 | 2.46 | 1.55E-02 |
| 7185 | AbstractArt | 3.58 | 1.89 | 2.56 | 2.13 | 2.73 | 7.23E-03 |
| 7186 | AbstractArt | 3.94 | 1.78 | 3.48 | 2.31 | 1.22 | 2.26E-01 |
| 7187 | AbstractArt | 3.84 | 1.81 | 3.16 | 1.63 | 2.11 | 3.74E-02 |
| 7190 | Clock | 4.42 | 1.93 | 3.89 | 1.97 | 1.46 | 1.47E-01 |
| 7195 | Teeth | 4.61 | 1.87 | 4.74 | 1.95 | -0.37 | 7.13E-01 |
| 7200 | Brownie | 5 | 2.09 | 4.9 | 2.67 | 0.23 | 8.21E-01 |
| 7205 | Scarves | 3.52 | 1.69 | 2.87 | 2.29 | 1.77 | 7.98E-02 |
| 7207 | Beads | 4.47 | 1.95 | 3.31 | 2.23 | 2.99 | 3.38E-03 |
| 7211 | Clock | 3.03 | 1.68 | 3.7 | 2.25 | -1.84 | 6.82E-02 |
| 7217 | clothesRack | 3.41 | 1.94 | 2.31 | 1.64 | 3.24 | 1.59E-03 |
| 7220 | Pastry | 5.78 | 1.85 | 4.65 | 2.19 | 3.03 | 3.06E-03 |
| 7224 | FileCabinets | 3.94 | 1.87 | 2.55 | 1.86 | 3.98 | 1.20E-04 |
| 7230 | Turkey | 5.88 | 1.9 | 5.81 | 2.25 | 0.18 | 8.56E-01 |
| 7233 | Plate | 3.59 | 1.87 | 2.51 | 1.74 | 3.18 | 1.88E-03 |
| 7234 | IroningBoard | 3.53 | 1.93 | 2.83 | 1.79 | 2 | 4.75E-02 |
| 7235 | Chair | 3.33 | 1.75 | 2.68 | 1.9 | 1.91 | 5.83E-02 |
| 7236 | Lightbulb | 4.44 | 1.86 | 4.08 | 2.3 | 0.94 | 3.51E-01 |
| 7237 | AbstractArt | 4.61 | 2.11 | 3.7 | 2.17 | 2.28 | 2.45E-02 |
| 7238 | AbstractArt | 5.8 | 1.73 | 4.24 | 2.66 | 3.83 | 2.11E-04 |
| 7250 | Cake | 5.06 | 2.05 | 4.76 | 2.17 | 0.77 | 4.43E-01 |
| 7260 | Torte | 5.03 | 1.52 | 4.88 | 2.16 | 0.44 | 6.60E-01 |
| 7270 | IceCream | 5.11 | 1.89 | 5.66 | 2.33 | -1.41 | 1.62E-01 |
| 7280 | Wines | 4.42 | 1.68 | 4.61 | 2.43 | -0.5 | 6.18E-01 |
| **7281** | **Food** | **5.92** | **1.74** | **4.33** | **2.1** | **4.47** | **1.82E-05^*^** |
| **7282** | **Cake** | **6.16** | **1.77** | **4.44** | **2.05** | **4.87** | **3.66E-06^*^** |
| **7283** | **Fruit** | **5.66** | **1.61** | **3.63** | **1.87** | **6.29** | **5.79E-09^*^** |
| 7284 | Fruit | 4.73 | 1.8 | 4.12 | 2.09 | 1.69 | 9.33E-02 |
| **7285** | **Tomatoes** | **5.17** | **1.74** | **3.52** | **1.74** | **5.07** | **1.53E-06^*^** |
| 7286 | Pancakes | 5.5 | 1.65 | 4.39 | 2.31 | 3.03 | 3.01E-03 |
| 7289 | Food | 5.36 | 1.77 | 5.08 | 2.64 | 0.69 | 4.95E-01 |
| 7291 | Chicken | 5.91 | 2.03 | 5.11 | 2.19 | 2.03 | 4.47E-02 |
| 7320 | Desserts | 4.31 | 1.6 | 4.94 | 1.91 | -1.94 | 5.50E-02 |
| **7325** | **Watermelon** | **5.8** | **1.72** | **3.24** | **2.06** | **7.32** | **3.71E-11^*^** |
| **7330** | **IceCream** | **6.14** | **1.49** | **4.54** | **2.55** | **4.26** | **4.25E-05^*^** |
| 7340 | IceCream | 4.59 | 1.68 | 3.69 | 2.53 | 2.31 | 2.27E-02 |
| 7350 | Pizza | 5.84 | 1.72 | 5.4 | 2.71 | 1.07 | 2.86E-01 |
| 7351 | Pizza | 5.27 | 1.72 | 4.11 | 2.15 | 3.24 | 1.56E-03 |
| 7352 | Pizza | 4.92 | 1.81 | 4.57 | 2.48 | 0.88 | 3.79E-01 |
| 7359 | PieW/bug | 5.48 | 1.92 | 5.07 | 2.09 | 1.11 | 2.68E-01 |
| 7360 | FliesOnPie | 5.77 | 1.61 | 4.52 | 2.17 | 3.58 | 5.07E-04 |
| **7361** | **MeatSlicer** | **7.52** | **1.94** | **4.73** | **2.47** | **6.84** | **3.99E-10^*^** |
| 7380 | RoachOnPizza | 5.5 | 1.86 | 5.53 | 2.56 | -0.07 | 9.42E-01 |
| 7390 | IceCream | 5.11 | 1.92 | 4.39 | 2.24 | 1.87 | 6.43E-02 |
| 7400 | Candy | 4.89 | 1.84 | 4.65 | 2.18 | 0.64 | 5.20E-01 |
| 7402 | Pastry | 5.64 | 2.03 | 4.94 | 2.05 | 1.84 | 6.82E-02 |
| 7410 | Candy | 5.06 | 1.98 | 5.24 | 2.16 | -0.47 | 6.41E-01 |
| 7430 | Candy | 5.78 | 1.72 | 4.45 | 2.35 | 3.54 | 5.87E-04 |
| **7450** | **Cheeseburger** | **6.31** | **1.85** | **4.67** | **2.17** | **4.4** | **2.40E-05^*^** |
| 7460 | FrenchFries | 6.22 | 1.86 | 5.04 | 2.56 | 2.88 | 4.69E-03 |
| 7470 | Pancakes | 5.63 | 1.8 | 4.54 | 2.34 | 2.85 | 5.21E-03 |
| 7472 | Grapes | 5.2 | 1.83 | 4.04 | 2.22 | 3.11 | 2.39E-03 |
| **7475** | **Shrimp** | **5.78** | **1.73** | **4.05** | **2.38** | **4.55** | **1.31E-05^*^** |
| 7480 | Pasta | 5.59 | 1.66 | 5.08 | 2.21 | 1.43 | 1.57E-01 |
| 7481 | Food | 5.84 | 2.01 | 4.72 | 1.96 | 3.02 | 3.08E-03 |
| 7490 | Window | 3.64 | 1.74 | 2.71 | 2.58 | 2.33 | 2.17E-02 |
| 7491 | Building | 3.22 | 1.55 | 2.6 | 1.95 | 1.92 | 5.79E-02 |
| 7493 | Man | 3.8 | 1.63 | 3.41 | 1.8 | 1.22 | 2.23E-01 |
| 7495 | Store | 4.13 | 1.8 | 3.98 | 2.35 | 0.38 | 7.06E-01 |
| 7496 | Street | 3.75 | 1.71 | 4.74 | 1.89 | -2.96 | 3.71E-03 |
| 7500 | Building | 4.33 | 1.75 | 3.46 | 2.23 | 2.37 | 1.96E-02 |
| **7501** | **City** | **4** | **1.6** | **5.85** | **2.09** | **-5.42** | **3.26E-07^*^** |
| 7502 | Castle | 5.7 | 1.88 | 5.74 | 1.97 | -0.11 | 9.11E-01 |
| 7503 | CardDealer | 4.55 | 1.67 | 4.82 | 2.53 | -0.69 | 4.89E-01 |
| 7504 | Stairs | 3.94 | 1.71 | 3.96 | 2.21 | -0.06 | 9.56E-01 |
| 7510 | Skyscraper | 4.55 | 2.11 | 4.93 | 2.11 | -0.96 | 3.37E-01 |
| 7545 | Ocean | 4.25 | 1.94 | 3.34 | 2.17 | 2.39 | 1.87E-02 |
| 7550 | Office | 3.69 | 1.75 | 4.48 | 1.98 | -2.28 | 2.43E-02 |
| 7560 | Freeway | 3.94 | 1.7 | 5 | 2.15 | -2.98 | 3.54E-03 |
| 7570 | Skyline | 5.22 | 1.79 | 6.27 | 1.98 | -3 | 3.32E-03 |
| 7580 | Desert | 5.89 | 1.99 | 5.08 | 2.62 | 1.9 | 5.95E-02 |
| 7590 | Traffic | 4.4 | 1.66 | 3.91 | 2.21 | 1.37 | 1.73E-01 |
| 7595 | Traffic | 3.52 | 1.81 | 3.25 | 2.25 | 0.71 | 4.81E-01 |
| 7600 | Dragon | 5.23 | 1.65 | 5.36 | 1.95 | -0.38 | 7.07E-01 |
| 7620 | Jet | 4.03 | 1.69 | 4.79 | 2.18 | -2.12 | 3.58E-02 |
| 7640 | Skyscraper | 4.63 | 2.03 | 6.21 | 2.54 | -3.74 | 2.90E-04 |
| 7700 | Office | 3.83 | 1.73 | 2.85 | 2.23 | 2.68 | 8.54E-03 |
| 7705 | Cabinet | 3.67 | 1.87 | 2.4 | 1.78 | 3.72 | 3.15E-04 |
| 7710 | Bed | 3.72 | 1.81 | 3.5 | 2.17 | 0.6 | 5.52E-01 |
| 7820 | Agate | 4.48 | 1.76 | 4.52 | 2.09 | -0.11 | 9.11E-01 |
| **7830** | **Agate** | **4.77** | **2.05** | **7.32** | **2.16** | **-6.51** | **2.10E-09^*^** |
| 7900 | Violin | 4.08 | 1.59 | 2.94 | 2.17 | 3.28 | 1.37E-03 |
| 7920 | CarCrash | 4.16 | 1.49 | 4.15 | 2.16 | 0.03 | 9.76E-01 |
| 7950 | Tissue | 3.63 | 1.74 | 2.3 | 1.89 | 3.93 | 1.47E-04 |
| 8010 | Runner | 4.05 | 1.55 | 4.12 | 2.13 | -0.21 | 8.37E-01 |
| 8021 | Skier | 5.3 | 1.69 | 5.67 | 2.42 | -0.97 | 3.32E-01 |
| **8030** | **Skier** | **4.66** | **1.79** | **7.32** | **2.16** | **-7.28** | **4.54E-11^*^** |
| 8031 | Skier | 6.22 | 1.19 | 5.6 | 2.28 | 1.91 | 5.91E-02 |
| **8032** | **IceSkater** | **6.03** | **1.4** | **3.62** | **1.73** | **8.32** | **2.00E-13^*^** |
| 8033 | IceSkater | 5.39 | 1.61 | 4.34 | 1.98 | 3.16 | 2.01E-03 |
| 8034 | Skier | 4.81 | 1.69 | 6.2 | 2.24 | -3.83 | 2.11E-04 |
| 8040 | Diver | 5.53 | 1.62 | 5.73 | 2 | -0.6 | 5.52E-01 |
| 8041 | Diver | 4.89 | 1.71 | 4.92 | 2.26 | -0.08 | 9.35E-01 |
| 8050 | Rower | 4.51 | 1.5 | 3.73 | 1.74 | 2.6 | 1.06E-02 |
| 8060 | Boxer | 6.44 | 1.39 | 5.9 | 2.07 | 1.68 | 9.62E-02 |
| **8080** | **Sailing** | **4.97** | **1.75** | **7.12** | **1.95** | **-6.26** | **6.85E-09^*^** |
| 8090 | Gymnast | 5.44 | 1.49 | 5.71 | 1.93 | -0.85 | 3.95E-01 |
| 8116 | Football | 4.42 | 1.79 | 5.69 | 2.42 | -3.26 | 1.47E-03 |
| 8117 | Hockey | 5.16 | 1.61 | 5.4 | 2.46 | -0.64 | 5.25E-01 |
| 8120 | Athlete | 4.9 | 1.72 | 5.15 | 2.09 | -0.71 | 4.80E-01 |
| 8130 | PoleVaulter | 5.11 | 1.91 | 5.32 | 2.06 | -0.57 | 5.71E-01 |
| **8160** | **RockClimber** | **5.44** | **1.73** | **6.9** | **1.62** | **-4.64** | **9.33E-06^*^** |
| 8161 | HangGlider | 4.55 | 1.81 | 5.93 | 2.27 | -3.66 | 3.85E-04 |
| 8162 | HotAirBalloon | 5.63 | 1.86 | 4.84 | 2.16 | 2.12 | 3.63E-02 |
| 8170 | Sailboat | 5.45 | 1.84 | 6.57 | 1.94 | -3.18 | 1.88E-03 |
| 8178 | Cliffdiver | 6.3 | 1.92 | 6.71 | 2.06 | -1.11 | 2.70E-01 |
| 8179 | Bungee | 5.23 | 2.24 | 6.86 | 2.26 | -3.86 | 1.85E-04 |
| 8180 | CliffDivers | 6.52 | 1.89 | 6.54 | 2.14 | -0.05 | 9.57E-01 |
| **8185** | **SkyDivers** | **5.45** | **1.88** | **7.06** | **2.09** | **-4.36** | **2.80E-05^*^** |
| **8186** | **Skysurfer** | **5.09** | **1.77** | **6.98** | **2.05** | **-5.33** | **5.01E-07^*^** |
| 8190 | Skier | 6.69 | 1.82 | 6.41 | 2.6 | 0.68 | 4.99E-01 |
| 8191 | IceClimber | 6.06 | 1.88 | 6.38 | 2.24 | -0.83 | 4.08E-01 |
| 8192 | VolcanoSkier | 5.14 | 1.95 | 6.46 | 1.9 | -3.66 | 3.83E-04 |
| 8193 | Skier | 5.23 | 1.62 | 5.64 | 2.15 | -1.16 | 2.47E-01 |
| 8200 | WaterSkier | 5.3 | 1.87 | 6.33 | 2.05 | -2.83 | 5.52E-03 |
| 8210 | Boat | 5.55 | 1.53 | 5.87 | 2.09 | -0.96 | 3.41E-01 |
| 8211 | Sailboat | 5.06 | 1.76 | 5.84 | 2.2 | -2.12 | 3.60E-02 |
| 8220 | Runners | 5.33 | 1.58 | 4.85 | 1.9 | 1.48 | 1.41E-01 |
| 8230 | Boxer | 6.98 | 1.86 | 5.75 | 2.16 | 3.31 | 1.26E-03 |
| 8231 | Boxer | 5.36 | 1.91 | 5.17 | 1.74 | 0.55 | 5.82E-01 |
| 8232 | Boxer | 4.47 | 1.68 | 5.29 | 2.49 | -2.13 | 3.54E-02 |
| 8250 | Motorcyclist | 4.55 | 1.85 | 5.5 | 2.56 | -2.34 | 2.11E-02 |
| 8251 | Motorcycle | 5.19 | 1.69 | 6.2 | 2.26 | -2.77 | 6.46E-03 |
| **8260** | **Motorcyclist** | **5.06** | **1.74** | **6.69** | **2.11** | **-4.58** | **1.21E-05^*^** |
| *8280* | *Diver* | *4.55* | *1.98* | *5.08* | *2.07* | *-1.4* | *1.63E-01* |
| 8300 | Pilot | 4.7 | 1.69 | 6 | 2.28 | -3.54 | 5.72E-04 |
| 8311 | Golfer | 4.59 | 1.82 | 4.02 | 2.69 | 1.37 | 1.73E-01 |
| 8320 | CarRacer | 4.05 | 1.84 | 4.46 | 2.42 | -1.04 | 3.00E-01 |
| 8330 | Winner | 4.92 | 1.82 | 3.87 | 2 | 2.96 | 3.75E-03 |
| **8340** | **Plane** | **4.3** | **1.72** | **6.22** | **2.44** | **-4.99** | **2.14E-06^*^** |
| 8341 | Wingwalker | 5.09 | 1.87 | 6.33 | 2.24 | -3.25 | 1.54E-03 |
| 8350 | TennisPlayer | 4.48 | 1.91 | 4.94 | 2 | -1.25 | 2.14E-01 |
| **8370** | **Rafting** | **4.81** | **1.94** | **6.46** | **2.22** | **-4.27** | **4.07E-05^*^** |
| 8380 | Athletes | 4.66 | 1.59 | 6.02 | 2.34 | -3.76 | 2.74E-04 |
| **8400** | **Rafters** | **4.53** | **1.75** | **7** | **1.56** | **-7.91** | **1.70E-12^*^** |
| 8420 | Tubing | 4.97 | 1.81 | 5.71 | 2.42 | -1.9 | 6.03E-02 |
| 8460 | Runner | 4.77 | 1.78 | 3.52 | 2.25 | 3.35 | 1.08E-03 |
| 8461 | HappyTeens | 5.39 | 1.66 | 4.85 | 2.22 | 1.51 | 1.35E-01 |
| **8465** | **Runner** | **4.91** | **1.69** | **2.82** | **1.93** | **6.23** | **8.04E-09^*^** |
| **8470** | **Gymnast** | **4.55** | **1.69** | **6.31** | **2.19** | **-4.91** | **3.07E-06^*^** |
| 8475 | Biking/train | 5.38 | 2.16 | 6.49 | 1.94 | -2.88 | 4.74E-03 |
| 8480 | BikerOnFire | 7.37 | 1.87 | 5.83 | 2.16 | 4.11 | 7.44E-05 |
| 8485 | Fire | 6.25 | 1.71 | 6.63 | 1.97 | -1.11 | 2.67E-01 |
| 8490 | RollerCoaster | 4.97 | 1.53 | 6.25 | 1.96 | -3.97 | 1.26E-04 |
| 8496 | WaterSlide | 6.06 | 1.57 | 5 | 2.21 | 3.03 | 3.00E-03 |
| 8497 | CarnivalRide | 4.33 | 1.5 | 3.89 | 1.92 | 1.39 | 1.67E-01 |
| 8500 | Gold | 7 | 2.14 | 5.71 | 2.55 | 2.97 | 3.60E-03 |
| 8501 | Money | 7.38 | 2.03 | 6.86 | 2 | 1.37 | 1.74E-01 |
| 8502 | Money | 6.52 | 2.19 | 5.48 | 2.41 | 2.43 | 1.65E-02 |
| 8503 | Money | 5.77 | 1.92 | 5.33 | 2.53 | 1.06 | 2.92E-01 |
| 8510 | SportCar | 6.08 | 1.88 | 5.44 | 2.58 | 1.55 | 1.24E-01 |
| 8531 | SportCar | 5.52 | 1.93 | 5.58 | 2.01 | -0.16 | 8.70E-01 |
| 8540 | Athletes | 4.98 | 1.76 | 4.96 | 2.34 | 0.05 | 9.58E-01 |
| 8600 | Mascot | 4.83 | 1.57 | 4.18 | 2.27 | 1.83 | 6.98E-02 |
| 8620 | Woman | 5.48 | 1.52 | 4.47 | 2.02 | 3.1 | 2.44E-03 |
| 9000 | Cemetery | 4.59 | 2.09 | 3.9 | 2.12 | 1.77 | 8.02E-02 |
| 9001 | Cemetery | 4.48 | 1.97 | 3.74 | 2.35 | 1.86 | 6.51E-02 |
| 9005 | HIVTattoo | 6.14 | 1.51 | 5.08 | 2.08 | 3.19 | 1.81E-03 |
| 9006 | HIVTattoo | 5.16 | 1.75 | 5.29 | 2.41 | -0.34 | 7.36E-01 |
| **9007** | **Needles** | **6.6** | **2.07** | **4.52** | **2.27** | **5.17** | **9.97E-07^*^** |
| **9008** | **Needle** | **6.56** | **1.93** | **4.16** | **2.05** | **6.48** | **2.38E-09^*^** |
| 9010 | BarbedWire | 3.94 | 2.16 | 4.32 | 1.89 | -1 | 3.20E-01 |
| **9040** | **StarvingChild** | **7.31** | **1.92** | **5.1** | **2.11** | **5.91** | **3.51E-08^*^** |
| 9041 | ScareChild | 4.22 | 1.58 | 4.3 | 2.21 | -0.23 | 8.15E-01 |
| **9042** | **StickThruLip** | **7.16** | **1.72** | **5.13** | **2.39** | **5.34** | **4.66E-07^*^** |
| **9045** | **NativeFem** | **5.83** | **1.87** | **3.37** | **1.97** | **6.87** | **3.41E-10^*^** |
| 9046 | Family | 4.55 | 2 | 3.85 | 1.55 | 2.05 | 4.29E-02 |
| 9050 | PlaneCrash | 5.13 | 2.15 | 6.05 | 2.01 | -2.37 | 1.96E-02 |
| 9070 | Boy | 4.33 | 1.83 | 3.51 | 2.17 | 2.21 | 2.88E-02 |
| 9080 | Wires | 4.67 | 1.88 | 4.1 | 2.13 | 1.53 | 1.28E-01 |
| 9090 | Exhaust | 4.7 | 1.87 | 4.22 | 2.07 | 1.31 | 1.92E-01 |
| 9101 | Cocaine | 3.64 | 1.88 | 4.17 | 2.42 | -1.33 | 1.85E-01 |
| 9102 | Heroin | 4.69 | 1.73 | 4.47 | 2.42 | 0.57 | 5.73E-01 |
| 9110 | Puddle | 3.52 | 1.74 | 3.9 | 2.18 | -1.06 | 2.91E-01 |
| 9120 | OilFires | 5.09 | 1.88 | 5.63 | 2.11 | -1.46 | 1.47E-01 |
| 9140 | Cow | 5.72 | 2.08 | 4.9 | 2.29 | 2.02 | 4.57E-02 |
| 9156 | Plane | 4.81 | 1.89 | 6.04 | 2.52 | -3.02 | 3.13E-03 |
| 9160 | Soldier | 4.39 | 1.87 | 5.66 | 1.96 | -3.56 | 5.40E-04 |
| 9171 | Fisher | 5.27 | 1.6 | 4.75 | 2.11 | 1.52 | 1.32E-01 |
| 9180 | Seal | 4.52 | 2.01 | 5.07 | 2.26 | -1.4 | 1.64E-01 |
| 9181 | DeadCows | 5.39 | 1.79 | 4.67 | 2.42 | 1.85 | 6.68E-02 |
| 9182 | Horses | 5.34 | 1.94 | 4.53 | 1.77 | 2.33 | 2.15E-02 |
| 9190 | Woman | 4.59 | 1.81 | 3.71 | 1.75 | 2.64 | 9.48E-03 |
| 9210 | Rain | 3.63 | 1.77 | 2.89 | 2.05 | 2.09 | 3.88E-02 |
| 9220 | Cemetery | 4.06 | 1.55 | 3.83 | 2.33 | 0.65 | 5.19E-01 |
| 9230 | OilFire | 5.55 | 1.97 | 5.67 | 2.37 | -0.3 | 7.66E-01 |
| 9250 | WarVictim | 5.61 | 1.82 | 6.5 | 1.66 | -2.72 | 7.63E-03 |
| 9252 | DeadBody | 5.94 | 1.93 | 6.27 | 2.3 | -0.84 | 4.01E-01 |
| **9253** | **Mutilation** | **6.98** | **1.89** | **5.38** | **2.16** | **4.26** | **4.19E-05^*^** |
| **9265** | **HungMan** | **5.94** | **1.92** | **4.15** | **2.12** | **4.77** | **5.43E-06^*^** |
| 9270 | ToxicWaste | 4.42 | 1.84 | 5.62 | 1.97 | -3.38 | 9.94E-04 |
| 9280 | Smoke | 4.77 | 2.14 | 4.55 | 2.54 | 0.51 | 6.13E-01 |
| 9290 | Garbage | 3.78 | 1.68 | 4.33 | 2.27 | -1.5 | 1.35E-01 |
| 9300 | Dirty | 5.92 | 2.37 | 5.69 | 2.54 | 0.5 | 6.16E-01 |
| 9301 | Toilet | 6.11 | 2.3 | 4.84 | 2.34 | 2.93 | 4.05E-03 |
| 9320 | Vomit | 5.98 | 2.32 | 4.5 | 2.52 | 3.29 | 1.33E-03 |
| 9330 | Garbage | 3.88 | 1.86 | 4.26 | 2.05 | -1.06 | 2.92E-01 |
| 9331 | HomelessMan | 3.75 | 1.83 | 3.42 | 1.67 | 1 | 3.18E-01 |
| 9340 | Garbage | 3.83 | 1.8 | 4.76 | 2.44 | -2.38 | 1.89E-02 |
| 9341 | Pollution | 4.72 | 1.95 | 4.26 | 1.99 | 1.25 | 2.13E-01 |
| 9342 | Pollution | 4.52 | 1.85 | 4.4 | 1.9 | 0.34 | 7.32E-01 |
| **9360** | **EmptyPool** | **3.98** | **1.72** | **2.49** | **1.83** | **4.51** | **1.59E-05^*^** |
| 9373 | Garbage | 4.72 | 1.85 | 4.35 | 1.88 | 1.06 | 2.90E-01 |
| 9390 | Dishes | 3.77 | 1.84 | 4.26 | 2.05 | -1.36 | 1.77E-01 |
| 9400 | Soldier | 5.56 | 2.08 | 5.5 | 2.18 | 0.15 | 8.80E-01 |
| 9401 | Knives | 4.06 | 1.76 | 3.67 | 1.99 | 1.12 | 2.65E-01 |
| 9402 | Mob | 5.02 | 1.99 | 4.94 | 2.24 | 0.2 | 8.39E-01 |
| 9404 | Soldiers | 4.92 | 1.77 | 4.6 | 2.11 | 0.89 | 3.75E-01 |
| 9405 | SlicedHand | 6.73 | 1.95 | 5.31 | 2.38 | 3.56 | 5.42E-04 |
| 9409 | MenW/guns | 4.89 | 1.98 | 5.52 | 1.95 | -1.71 | 8.93E-02 |
| 9410 | Soldier | 6.58 | 1.74 | 6.38 | 2.26 | 0.54 | 5.94E-01 |
| 9411 | Boy | 5.44 | 1.62 | 5.61 | 1.86 | -0.54 | 5.93E-01 |
| 9415 | Handicapped | 5.16 | 1.84 | 4.5 | 2.29 | 1.73 | 8.67E-02 |
| 9417 | Ticket | 3.66 | 1.68 | 4.37 | 2.12 | -2.02 | 4.58E-02 |
| **9420** | **Soldier** | **6.67** | **1.81** | **5.1** | **2.02** | **4.42** | **2.22E-05^*^** |
| 9421 | Soldier | 4.53 | 1.83 | 4.86 | 2.19 | -0.89 | 3.77E-01 |
| 9430 | Burial | 5.25 | 1.73 | 4.81 | 2.54 | 1.11 | 2.68E-01 |
| 9432 | Mastactomy | 5.09 | 1.91 | 4.12 | 2.13 | 2.6 | 1.07E-02 |
| 9433 | DeadMan | 6 | 1.99 | 5 | 2.65 | 2.33 | 2.14E-02 |
| 9435 | Accident | 4.59 | 1.9 | 4.9 | 1.83 | -0.88 | 3.82E-01 |
| 9440 | Skulls | 5.96 | 2.17 | 4.71 | 1.97 | 3.2 | 1.75E-03 |
| 9452 | Gun | 5.7 | 1.56 | 4.5 | 2.23 | 3.43 | 8.30E-04 |
| 9470 | Ruins | 4.77 | 1.93 | 4.53 | 1.68 | 0.7 | 4.84E-01 |
| 9471 | BurntBldg | 4.48 | 2.07 | 4.43 | 2.03 | 0.14 | 8.88E-01 |
| 9472 | Bridge | 3.95 | 2.04 | 4.31 | 1.99 | -0.95 | 3.47E-01 |
| 9480 | Skull | 5.16 | 1.99 | 5.15 | 1.69 | 0.03 | 9.77E-01 |
| 9490 | Corpse | 5.95 | 2.08 | 5.41 | 2.26 | 1.34 | 1.83E-01 |
| 9495 | Fire | 5.11 | 1.57 | 5.71 | 1.89 | -1.87 | 6.37E-02 |
| 9500 | Porpoises | 5.06 | 2.06 | 5.65 | 2.45 | -1.41 | 1.60E-01 |
| 9520 | Kids | 4.36 | 1.58 | 5.45 | 2.54 | -2.86 | 5.10E-03 |
| 9530 | Boys | 5.05 | 1.86 | 4.47 | 2.36 | 1.49 | 1.40E-01 |
| 9560 | DuckInOil | 4.44 | 1.91 | 5.46 | 2.6 | -2.45 | 1.59E-02 |
| 9561 | SickKitty | 5.09 | 1.83 | 4.18 | 2.19 | 2.45 | 1.60E-02 |
| 9570 | Dog | 5.97 | 2.38 | 5.84 | 2.41 | 0.29 | 7.74E-01 |
| 9571 | Cat | 5.59 | 2.12 | 4.68 | 2.35 | 2.19 | 3.04E-02 |
| 9582 | DentalExam | 4.22 | 1.57 | 5.15 | 1.99 | -2.83 | 5.49E-03 |
| 9584 | DentalExam | 4.31 | 1.95 | 4.65 | 2.32 | -0.85 | 3.95E-01 |
| 9592 | Injection | 4.17 | 1.8 | 5.11 | 2.07 | -2.61 | 1.02E-02 |
| 9594 | Injection | 4.3 | 1.94 | 4.55 | 2.17 | -0.66 | 5.14E-01 |
| 9600 | Ship | 6.03 | 1.96 | 6.2 | 2.13 | -0.45 | 6.56E-01 |
| 9611 | PlaneCrash | 4.81 | 1.71 | 5.5 | 2.5 | -1.77 | 7.90E-02 |
| 9620 | Shipwreck | 5.45 | 1.58 | 5.88 | 2.1 | -1.26 | 2.09E-01 |
| 9621 | Ship | 4.84 | 1.92 | 5.64 | 2.02 | -2.18 | 3.13E-02 |
| 9622 | Jet | 6.53 | 1.74 | 6.15 | 2.15 | 1.06 | 2.93E-01 |
| 9630 | Bomb | 5.91 | 1.85 | 6.29 | 2.38 | -0.97 | 3.33E-01 |
| 9635.1 | ManOnFire | 5.42 | 1.84 | 6.29 | 2.06 | -2.4 | 1.81E-02 |
| 9700 | Trash | 4.09 | 1.65 | 3.11 | 1.98 | 2.92 | 4.26E-03 |
| 9800 | Skinhead | 4.59 | 1.51 | 5.96 | 2.66 | -3.53 | 6.08E-04 |
| 9810 | KKKrally | 5.78 | 1.88 | 6.74 | 2.33 | -2.47 | 1.52E-02 |
| 9830 | Cigarettes | 4.25 | 1.92 | 4.8 | 2.72 | -1.28 | 2.02E-01 |
| 9910 | CarAccident | 5.27 | 2.03 | 6 | 2.1 | -1.9 | 6.05E-02 |
| 9911 | CarAccident | 4.81 | 1.73 | 5.15 | 1.99 | -0.99 | 3.26E-01 |
| 9912 | Firefigher | 4.84 | 1.96 | 4.67 | 2.31 | 0.43 | 6.68E-01 |
| 9913 | Truck | 4.27 | 1.51 | 4.45 | 2.06 | -0.56 | 5.78E-01 |
| 9920 | CarAccident | 4.61 | 1.91 | 5.67 | 2.13 | -2.83 | 5.49E-03 |
| 9921 | Fire | 5.55 | 1.6 | 6.09 | 1.88 | -1.68 | 9.66E-02 |

^#^ The data about American participants were collected by Lang.

*P<6×10^-5^
